# In vitro metabolism study of ADB‐P‐5Br‐INACA and ADB‐4en‐P‐5Br‐INACA using human hepatocytes, liver microsomes, and in‐house synthesized references

**DOI:** 10.1002/dta.3773

**Published:** 2024-07-23

**Authors:** Tobias Rautio, Robin Obrist, Lucas Krebs, Therése Klingstedt, Johan Dahlén, Xiongyu Wu, Henrik Gréen

**Affiliations:** ^1^ Department of Physics, Chemistry and Biology Linköping University Linköping Sweden; ^2^ University of Applied Sciences Northwestern Windisch Switzerland; ^3^ Department of Forensic Genetics and Forensic Toxicology, National Board of Forensic Medicine Linköping Sweden; ^4^ Division of Drug Research, Department of Biomedical and Clinical Sciences Linköping University Linköping Sweden

**Keywords:** ADB‐4en‐P‐5Br‐INACA, ADB‐P‐5Br‐INACA, LC‐QTOF‐MS, metabolism, synthesized references

## Abstract

Synthetic cannabinoids (SCs) remain a major public health concern, as they continuously are linked to severe intoxications and drug‐related deaths worldwide. As new SCs continue to emerge on the illicit drug market, an understanding of SC metabolism is needed to identify formed metabolites that may serve as biomarkers in forensic toxicology screening and for understanding the pharmacokinetics of the drugs. In this work, the metabolism of ADB‐4en‐P‐5Br‐INACA and ADB‐P‐5Br‐INACA ((*S*)‐*N*‐(1‐amino‐3,3‐dimethyl‐1‐oxobutan‐2‐yl)‐5‐bromo‐1‐(pent‐4‐en‐1‐yl)‐1*H*‐indazole‐3‐carboxamide, (*S*)‐*N*‐(1‐amino‐3,3‐dimethyl‐1‐oxobutan‐2‐yl)‐5‐bromo‐1‐pentyl‐1*H*‐indazole‐3‐carboxamide respectively) were investigated using human hepatocytes in vitro and in‐house synthesized references. Both SCs were incubated with pooled human hepatocytes over 3 h, with the aim to identify unique and abundant metabolites using liquid chromatography–quadrupole time‐of‐flight mass spectrometry (LC‐QTOF‐MS). In total nine metabolites were identified for ADB‐4en‐P‐5Br‐INACA and 10 metabolites for ADB‐P‐5Br‐INACA. The observed biotransformations included dihydrodiol formation, terminal amide hydrolysis, hydroxylation, dehydrogenation, carbonyl formation, glucuronidation, and combinations thereof. The major metabolites were confirmed by in‐house synthesized references. Recommended biomarkers for ADB‐P‐5Br‐INACA and ADB‐4en‐P‐5Br‐INACA are the terminal hydroxy and dihydrodiol metabolite respectively.

## Graphical abstract

Analyzing hepatocytes and liver microsomes samples along with synthesized references. Complete structure elucidation of major metabolites was possible, and biomarkers for both ADB‐P‐5Br‐INACA and ADB‐4en‐P‐5Br‐INACA were suggested.

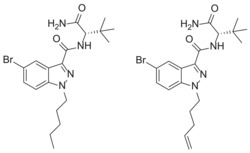

## 1 INTRODUCTION

New psychoactive substances (NPSs), also known as legal highs or designer drugs, refer to substances that have psychoactive effects that are similar to those of traditional drugs of abuse.^1^ One of the largest groups of NPS is synthetic cannabinoids (SCs), also known as synthetic cannabinoid receptor agonists (SCRAs). SCs are designed to mimic the effects of Δ9‐THC, as they act as cannabinoid receptor (CB1 and/or CB2) agonists. However, many SCs have shown to have a greater potency compared to Δ9‐THC.^2^ The first SCs appeared in 2004 as herbal mixtures called “spice” all over Europe and gained popularity around 2008 on the illicit drug market.^2^, ^3^ Since the appearance of SCs, numerous negative effects including seizures, reduced consciousness, anxiety, aggression, tachycardia, heart attack, cardiac arrest, respiratory depression, and death have been linked to SC intake.^4^ As of 2022, the total number of reported SCs that are being monitored in Europe is 245.^1^

To ensure safe identification of SCs and detection of their abuse via analysis of biomarkers, forensic laboratories require access to reference material of the parent substance and its metabolites. Normally, SCs are rapidly metabolized, and it is therefore often difficult to detect the parent substance in urine samples. However, the metabolites can serve as urinary biomarkers to reveal the abused SC.^5^

Urine is a common matrix for drug‐testing. This is due to the high metabolite concentration in urine, a longer detection window as compared to blood or oral fluid and the noninvasive collection of urine.^5^, ^6^, ^7^ Finding authentic human urine samples containing SC metabolites is often difficult. In the absence of urine samples, in vitro studies using human hepatocytes (HHep) have proven to be successful for metabolism studies of SCs, but also NPS in general. Several studies have shown that HHep can produce the same metabolites that are found in authentic urine samples. Hydroxylated metabolites are some of the metabolites that are often found in both urine and HHep samples.^7^, ^8^, ^9^, ^10^, ^11^, ^12^, ^13^, ^14^ Metabolites that are found in these HHep experiments therefore have the potential to serve as biomarkers in forensic toxicology screening. HHep experiments in combination with synthesis of potential metabolites make it possible to establish the full identity of the metabolites. It is virtually impossible to fully distinguish between different monohydroxylated metabolites without the use of synthesized reference substances.^15^

In 2021 China implemented stricter regulations of SCs that involved the regulation of indole and indazole core SCs.^16^ As an effect of this, new types of halogenated SCs have entered the recreational drug market. ADB‐P‐5Br‐INACA was reported in Portugal 2022.^17^ To the author's knowledge, there is not much information about these compounds. However, substitution of bromide onto the indazole core may greatly increase their potency.^18^

In this study, the metabolism of ADB‐P‐5Br‐INACA and its analogue ADB‐4en‐P‐5Br‐INACA was studied using HHep. The samples were analyzed after incubation using ultrahigh performance liquid chromatography–quadrupole time‐of‐flight mass spectrometry (UHPLC‐QTOF‐MS). To fully identify the exact chemical structures of the formed metabolites, potential metabolites were synthesized and analyzed with the same UHPLC‐QTOF‐MS method and obtained retention times, and mass spectral data were compared to the corresponding data of the incubation samples. The aim was to fully characterize key metabolites that have the potential to serve as biomarkers in forensic toxicology screening. The study did also aim at improving the understanding of SC metabolism that could enable prediction of the metabolic pathways of future SCs with a halogenated indazole core.

## 2 METHODOLOGY

### 2.1 Chemicals and reagents

Reference substances of ADB‐P‐5Br‐INACA and ADB‐4en‐P‐5Br‐INACA were synthesized in‐house. Williams E medium, L‐glutamine, HEPES buffer, and trypan blue were purchased from Thermo Fisher Scientific (Gothenburg, Sweden). Human liver microsomes (HLM), InVitroGro HT thawing medium and mixed gender primary HHep were obtained from BioIVT (West Sussex, UK). NADPH Regenerating System Solutions A and B were purchased from Corning Discovery Labware (Arizona, USA). 96‐well plates for incubation and subsequent analysis were purchased from Agilent Technologies (Sundbyberg, Sweden). Sodium hydride (NaH), triethylamine (EtN), alkylating agents (bromides), and solvents were obtained from Merck (Stockholm, Sweden). *L*‐*tert*‐leucine methyl ester hydrochloride, *L*‐*tert*‐leucinamide hydrochloride, and 2‐(1*H*‐Benzotriazole‐1‐yl)‐1,1,3,3‐tetramethylaminium tetrafluoroborate (TBTU) were obtained from Flurochem (Hadfield, UK), while 5‐bromo‐1*H*‐indazole‐3‐carboxylic acid was from VWR (Karlskoga, Sweden). Acetonitrile LC–MS grade was purchased from VWR (Stockholm, Sweden), and formic acid for LC–MS was obtained from Fisher Scientific (Gothenburg, Sweden).

### 2.2 Instrumentation

LC‐QTOF analyses were made on an Agilent 1290 Infinity UHPLC system coupled to an Agilent 6550 iFunnel QTOF mass spectrometer with an AJS Jet Stream Technology ion source, all from Agilent Technologies (Sundbyberg, Sweden). The chromatographic separation was performed on an ACQUITY Premier HSS T3 column (1.8 μm, 2.1 × 150 mm) equipped with an ACQUITY Premier HSS T3 pre‐column (1.8 μm, 2.1 × 5 mm), obtained from Waters (Solna, Sweden) that was maintained at 60°C. The mobile phase (0.5 mL/min) consisted of water with 0.1% formic acid (solvent A) and acetonitrile with 0.1% formic acid (solvent B). The gradient profile was as follows: 1% B (0–0.6 min), 1%–20% (0.6–0.7 min), 20%–75% (0.7–13.0 min), 75%–95% (13–15 min), 95% (15–18.0 min), 95%–1% (18–18.1 min), and 1% (18.1–19.0 min).

The QTOF was operated in positive electrospray mode (gas temperature: 150°C; gas flow: 18 L/min; nebulizer: 50 psi; sheath gas temperature: 375°C; sheath gas flow: 11 L/min). MS data were obtained using Data Dependent Auto MSMS (scan rate: 6 spectra/s (MS) and 10 spectra/s (MSMS); scan range: *m/z* 100–950 (MS) and *m/z* 50–950 (MSMS); precursor intensity threshold: 5000 counts; precursor number per cycle: 5, within *m/z* 200–800; fragmentor voltage: 380 V; collision energy: 3 eV at *m/z* 0 ramped up by 8 eV per *m/z* 100).

Details of NMR and single quadrupole LC–MS analysis are shown in the Supporting Information.

### 2.3 HHep incubation

Hepatocyte incubations, to investigate the metabolism of the compounds, were performed according to previously published protocols with minor modifications.^19^, ^20^ Briefly, HHep were thawed at 37°C, diluted in 48 mL thawing medium and pelleted by centrifugation at 100 ×*g*. The pellet was washed with 50 mL incubation medium and subsequently resuspended again in 2 mL incubation medium (Williams E medium supplemented with 2mM L‐glutamine and 20mM HEPES). The cell concentration was then adjusted to 2 × 10^6^ viable cells per milliliter, after determination with the trypan blue exclusion method.^21^ Working solutions of the SCs were prepared by dissolving the compound in methanol, followed by dilution using the incubation medium to a concentration of 10 μM. In a 96‐well plate, 50 μL of the working solution was mixed with 50 μL of the cell suspension in duplicate, resulting in a final substrate concentration of 5 μM and 1 × 10^6^ cells per milliliter. The cells were incubated for 0, 0.5, 1, and 3 h at 37°C. Incubation was terminated by the addition of 100 μL ice‐cold acetonitrile, and the samples were placed in a freezer for at least 10 min. For the 0 h samples, ice‐cold acetonitrile was added prior to the cell suspension. Diclofenac at a concentration of 10 μM was used as a positive control. Negative samples (without drug) and degradation samples (without cells) were incubated for 3 h. All samples were centrifuged at 1100 ×*g* for 15 min at 4°C. A volume of 120 μL was transferred to an injection plate and stored at −20°C until LC‐QTOF analysis.

### 2.4 Human liver microsomes incubation

ADB‐4en‐P‐5Br‐INACA and ADB‐P‐5Br‐INACA were also incubated with human liver microsomes for conformation analysis using synthesized reference standards. Stock solutions of ADB‐4en‐P‐5Br‐INACA and ADB‐P‐5Br‐INACA were prepared at a concentration of 500 μM in DMSO. ADB‐4en‐P‐5Br‐INACA (4 μL) or ADB‐P‐5Br‐INACA (4 μL) was then added to a solution containing 80 μL 0.5 M potassium phosphate buffer pH 7.4, 20 μL NADPH System Solution A, 4 μL NADPH System Solution B, and 282 μL water. After incubating the tubes at 37°C for 5 min, 10 μL human liver microsomes or 10 μL water was added resulting in a total reaction volume of 400 μL. The solution was mixed by inverting the tubes twice. The tubes were incubated at 37°C for 1 h before stopping the reaction by adding 400 μL acetonitrile and putting the tubes on ice. After a short centrifugation step (12,000 ×*g*, 3 min), the supernatants were collected and analyzed by LC‐QTOF.

### 2.5 Data analysis

Data analysis was performed using MassHunter software for Qualitative Analysis (version B.07.00). The search for metabolites was performed using a library containing the known biotransformations together with the find by formula algorithm that is utilized by the MassHunter Qualitative Analysis software. The database library contained known biotransformations for SCs, including hydroxylations, dihydrodiol formation, (de)methylation, dehydrogenation, *N*‐dealkylation, amide hydrolysis, glucuronidation, defluorination, sulfation, and combinations thereof. Only peaks with mass error <5 ppm were considered unless the peak was saturated. Compounds detected in the negative and the degradation controls were not evaluated as metabolites.

### 2.6 References synthesis

**FIGURE 1:**
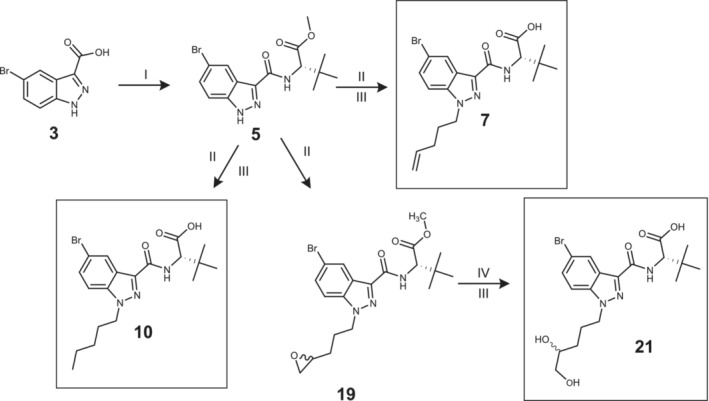
Synthetic route of reference material **7**, **10** and **21**. I: L‐*tert*‐leucine methyl ester hydrochloride (**4**), 2‐(1*H*‐Benzotriazole‐1‐yl)‐1,1,3,3‐tetramethylaminium tetrafluoroborate (TBTU), acetonitrile/dimethylformamide (ACN/DMF), TEA, 60°C ➔ rt, 23 h. II: 5‐Bromopent‐1‐ene (**1**), 2‐(3‐bromopropyl)oxirane (**2**) or 1‐Bromopentane (**8**), potassium carbonate (K_2_CO_3_), DMF, rt 21 h. III: 1 M aq. NaOH, rt, 3–21 h. IV: Triflic acid, THF, 120°C, 40 min. (numbering according to Supporting Information).

**FIGURE 2:**
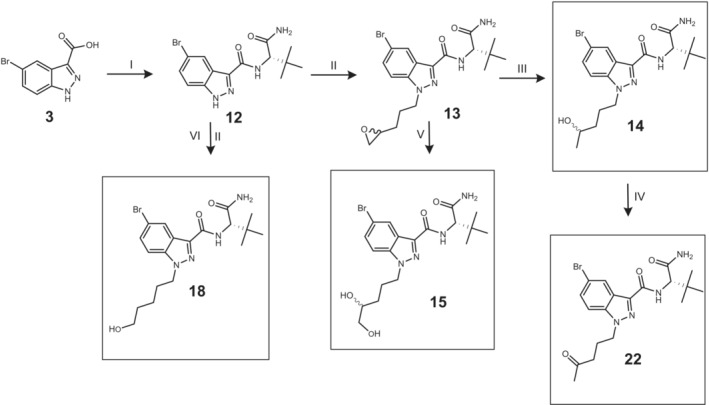
Synthetic route of reference material **14**, **15**, **18** and **22**. I: (2S)‐2‐amino‐3,3‐dimethylbutanamide (**11**), 2‐(1*H*‐Benzotriazole‐1‐yl)‐1,1,3,3‐tetramethylaminium tetrafluoroborate (TBTU), acetonitrile/dimethylformamide (ACN/DMF), TEA, 60°C, 5 h. II: 2‐(3‐bromopropyl)oxirane (**2**) or 1‐Bromopentyl acetate (**16**), potassium carbonate (K_2_CO_3_), DMF, rt, 21 h. III: NaBH_4_, 2‐propanol, 60°C ➔ rt, 23 h. IV: Dess–Martin, DCM, rt, 72 h. V: Triflic acid, water, THF, 120°C, 2 h. VI:1 M aq. NaOH, rt, 4 h. (numbering according to Supporting Information).

**FIGURE 3:**
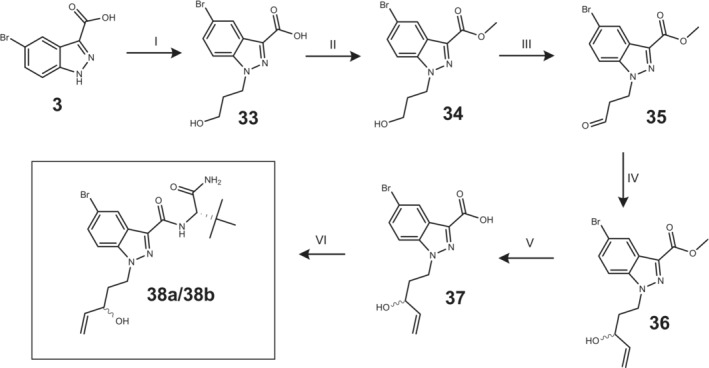
Synthetic route of reference material **38**. I: 1‐Bromopropanol, sodium hydride (NaH), dimethylformamide (DMF), 0°C ➔ rt, 6 h. II: 2 M Trimethyl silyl diazomethane, MeOH and toluene, rt, 40 min. III: Dess–Martin, DCM, rt, 21 h. IV: Vinylmagnesium bromide, THF, rt, 1 h. V: 1 M aq. NaOH, MeOH, THF, rt, 96 h. VI: (2S)‐2‐amino‐3,3‐dimethylbutanamide (**11**), 2‐(1*H*‐Benzotriazole‐1‐yl)‐1,1,3,3‐tetramethylaminium tetrafluoroborate (TBTU), acetonitrile/dimethylformamide (ACN/DMF), TEA, rt, 21 h. (numbering according to Supporting Information).

Reference substances of potential metabolites were synthesized to confirm the exact chemical structures of the metabolites formed in the HHep and HLM experiments. A total of eight potential metabolites were synthesized (Figures 1, 2, 3), four metabolites for each of the SCs ADB‐P‐5Br‐INACA (**10**, **14**, **18**, and **22**) and ADB‐4en‐P‐5Br‐INACA (**7**, **15**, **21**, and **38a**/**38b**). The synthesis are similar to previously presented method but with some modifications.^22^, ^23^ In general (except compound **38**), each synthetic procedure started with an amide coupling of compound **3** (Figures 1, 2) using either L‐*tert*‐Leucine methyl ester hydrochloride or (2S)‐2‐amino‐3,3‐dimethylbutanamide hydrochloride, together with TBTU, TEA and acetonitrile/dimethylformamide (ACN/DMF). This was followed by an alkylation procedure using an organobromide and potassium carbonate (K_2_CO_3_). A more detailed description of each synthetic procedure can be found in the Supporting Information.

Compound **38** started with alkylation of compound **3** using 1‐bromopropanol together with NaH in DMF (Figure 3). Compound **33** was then converted into its corresponding methyl ester compound **34**, followed by oxidation with Dess–Martin reagent giving compound **35** (Figure 3). Grignard reaction giving **36**, which was followed by basic hydrolysis giving compound **37** (Figure 3). Compound **38** was then obtained as a diastereomeric pair **38a**/**38b** via amide coupling. A more detailed description of the synthetic procedure of each of the specific compounds can be found in the Supporting Information.

### 2.7 Strategy for metabolite identification

In this study, both HHep and HLM studies were conducted. First, HHep incubations were made to tentatively identify the formed metabolites. Thereafter, a number of reference substances were selected for synthesis based upon the HHep results. These references were synthesized (**7**, **10**, **14**, **15**, **18**, **21**, **22**, and **38a**/**38**) and were subsequently analyzed by LC‐QTOF along with HLM incubated samples to elucidate the exact structure of the metabolites. The structures of all synthesized references were confirmed using NMR (Supporting Information). All synthesized reference standards were diluted to 2.5 μM methanolic solutions that were analyzed with the same LC‐QTOF method as the HHep samples and HLM incubations (Section 2.2). Hence, MSMS spectra and retention times of the references and the potential metabolites formed in the HLM and HHep experiments could be compared to enable metabolite identification.

## 3 RESULT AND DISCUSSION

### 3.1 ADB‐P‐5Br‐INACA metabolites formed in HLM and HHep incubations

The metabolite 4‐OH‐diclofenac was identified in the positive control (data not shown), displaying active HHep cells and a successful metabolism. The HHep incubation of ADB‐P‐5Br‐INACA resulted in 10 phase I metabolites (Table 1) that fulfilled the criteria defined in Section 2.5. The metabolites eluted between 6.12 and 13.05 min and the parent drug eluted at 12.09 min. The metabolites were assigned metabolite IDs according to their retention time. All relevant data such as metabolite ID rank according to total peak area, biotransformation, chemical formula, retention time, *m/z* of the protonated molecule, minimum and maximum mass error (ppm), identifying fragments, and relative abundance (peak area) in comparison to the most abundant metabolite as well as the total peak area of all metabolites are presented in Table 1. All analytical data such as extracted ion chromatograms (EIC) and MSMS spectra can be found in the Supporting Information.

**TABLE 1:** Results of the metabolism study of ADB‐P‐5Br‐INACA after hepatocyte incubation.

Metabolite ID	Metabolite rank	Biotransformation	Chemical formula	Average RT (min)	Average [M + H]^+^	Mass error (ppm)		Peak area (×10^3^)							Identifying fragments [M + H]^+^	% of highest metabolite*	% sof all metabolites
					m/z	Min	Max	0.5h_1	0.5h_2	1h_1	1h_2	3h_1	3h_2	Total	*m/z*		
P‐A	N/A (Parent)	n/a (Parent)	C19 H27 Br N4 O2	12.09	423.1395	0.9	3.6	7552	7250	3122	3170	1911	2943	25,948	293.0, 222.9	n/a	n/a
A1	5	Dihydroxylation (H + T)	C19 H27 Br N4 O4	6.12	455.1290	−0.2	0.72	59	65	98	113	135	171	640	309.0, 291.0, 222.9, 69.0	51.3	9.9
A2	9	Dehydrogenation (H) + dihydroxylation (H + T)	C19 H25 Br N4 O4	6.30	453.1134	−0.2	1.6	20	n.d.	43	46	101	113	324	309.0, 291.0, 222.9, 69.0	25.9	5.0
A3	7	Dehydrogenation (T) + dihydroxylation (H + T)	C19 H25 Br N4 O4	6.50	453.1134	0.1	0.7	27	52	83	88	70	138	458	307.0, 222.9, 85.0	36.6	7.1
A4	8	Dihydroxylation (H + T)	C19 H27 Br N4 O4	6.56	455.1282	−2.0	0.42	45	51	79	59	74	95	404	309.0, 291.0, 222.9, 69.0	32.3	6.3
A5*	3	Mono‐hydroxylation (T, 5OH)	C19 H27 Br N4 O3	7.97	439.1341	0.3	0.6	177	201	159	194	136	178	1046	309.0, 291.0, 263.0, 222.9, 69.0	83.7	16.2
A6	10	Amide hydrolysis (H) + mono‐hydroxylation (T)	C19 H26 Br N3 O4	9.06	440.1181	−0.9	1.0	n.d.	n.d.	27	29	54	64	174	291.0, 263.0, 222.9, 85.1	13.9	2.7
A7	11	Amide hydrolysis (H) + mono‐hydroxylation (T)	C19 H26 Br N3 O4	9.54	440.1170	−3.31	0.89	n.d.	n.d.	28	30	54	51	164	309.0, 291.0, 222.9, 69.0	13.1	2.5
A8	2	Mono‐hydroxylation (H)	C19 H27 Br N4 O3	10.01	439.1338	−0.3	1.2	231	242	166	202	133	178	1152	293.0, 222.9, 71.0	92.2	17.9
A9	4	Dehydrogenation (H) + mono‐hydroxylation (H)	C19 H25 Br N4 O3	10.09	437.1180	−0.9	−0.6	77	72	100	130	200	249	828	293.0, 222.9, 85.0	66.2	12.9
A10*	1	Amide hydrolysis (H)	C19 H26 Br N3 O3	13.05	424.1231	0.0	1.0	131	120	170	197	292	341	1250	293.0, 222.9	100	19.4

*Note*: Liquid chromatography–quadrupole time‐of‐flight mass spectrometry (LC‐QTOF‐MS) analysis and data selection including the following data: metabolite ID, metabolite rank, biotransformation, chemical formula, average retention time (min), average m/z of [M + H] + ions, min and max mass errors (ppm), peak areas (×10^3^) of the 0.5, 1, and 3 h samples, as well as the total area, the identifying fragments (m/z), and the relative abundance (%) that is given in relation to the total area of the highest metabolite (A11) as well as the total area of all metabolites. The location of a particular biotransformation is indicated by H = head‐group or T = tail‐group. Asterisk = identified using reference substance. More detailed structural information is provided in Figure 4.

Abbreviations: n/a, not applicable; n.d., not detected.

**FIGURE 4:**
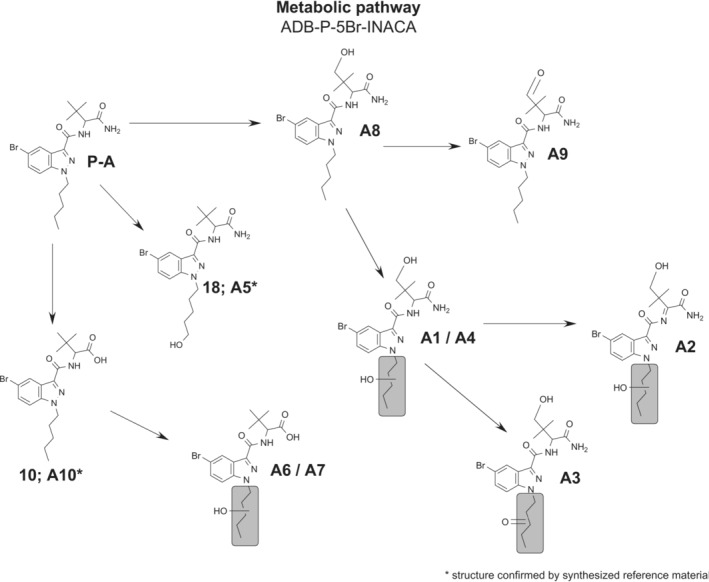
Proposed metabolic pathway of ADB‐P‐5Br‐INACA. Gray areas mark potential positions for hydroxylation/dehydrogenation.

**FIGURE 5:**
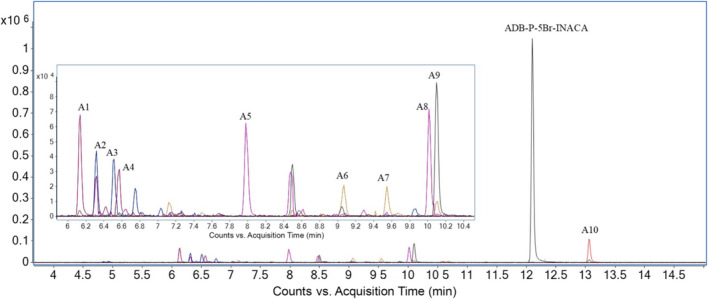
Overlayed extracted ion chromatograms (EIC) form human hepatocytes (HHep) of ADB‐P‐5Br‐INACA and all its identified metabolites marked with the corresponding metabolite IDs.

The observed biotransformations included amide hydrolysis, hydroxylation, carbonyl formation, dehydrogenation, and combinations thereof (Figure 4). The most abundant metabolite was A10 that was formed through amide hydrolysis. The second most abundant metabolite was the monohydroxy metabolite A8, where the hydroxy group was located at the *tert*‐butyl moiety. The third most abundant metabolite was A5, a monohydroxy metabolite with the hydroxy group at the terminal position of the pentyl side chain that was confirmed by analysis of reference.^18^ The metabolites, A10, A8, and A5, accounted for 53.5% of the total peak area of all metabolites. The proposed metabolic pathway is presented in Figure 4, as well as the overlayed EIC of all metabolites found in the HHep experiment is presented in Figure 5.

Hydroxylation was the most common biotransformation, as all metabolites except A11 included at least one addition of oxygen (+15.996 Da). The location of the hydroxy group was mainly identified through the *m/z* 309 pentyl indazole fragment (indazole core and tail) and the corresponding *m/z* 291 fragment that presented the neutral loss of water. With the additional fragment of *m/z* 222 that represents the indazole core without any modifications, it was possible to determine if the hydroxy group was located at the pentyl side chain (A1, A2, A4, A5, A6, A7). If fragment *m/z* 293 was detected, hydroxylation of the pentyl side chain could be excluded, and the hydroxy group was then most likely located at the *tert*‐butyl moiety (A8).

A combination of hydroxylation and dehydrogenation mainly presents a carbonyl formation, as seen in A9 and A3. The structures were identified through the *m/z* 85 fragment that represents a carbonyl formation either at the pentyl chain or at the *tert*‐butyl group (as both structures have the same mass). If the carbonyl was located at the pentyl side chain or not, it could be determined by the presence of fragment *m/z* 307 (A3) or 293 (A9). Looking at the MSMS spectrum for metabolite A2, there is no fragment indicating carbonyl formation clearly. However, there is a possibility of dehydrogenation of the carboxamide bond, which is also a common biotransformation for related structures.^24^ This is indicated by the double bond (A2) shown in Figure 4.

**FIGURE 6:**
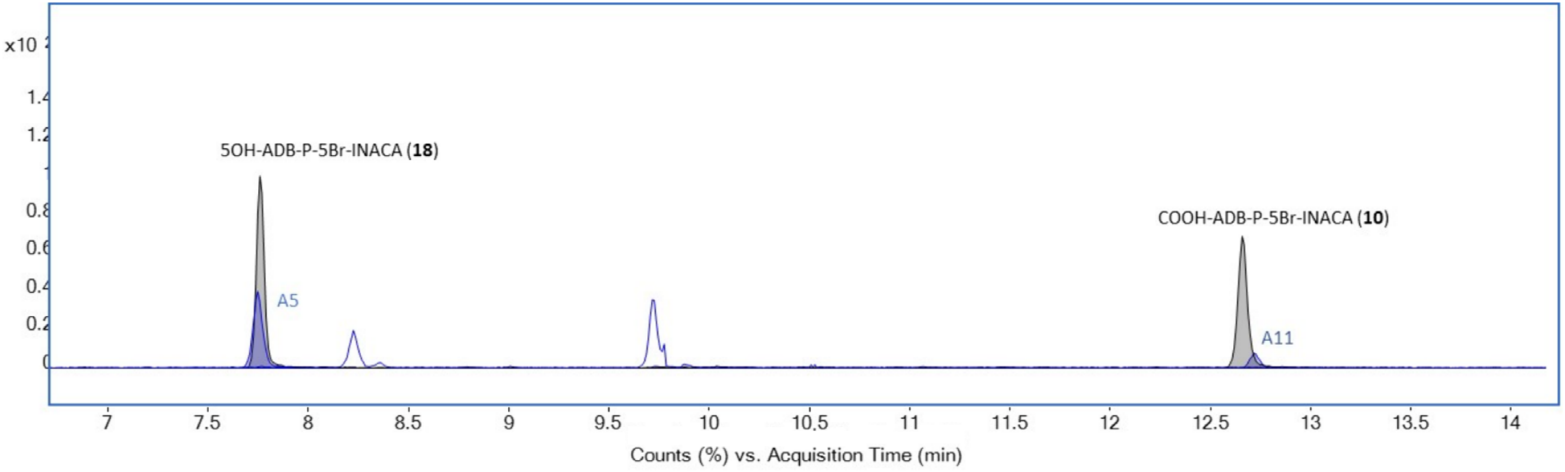
Overlayed extracted ion chromatograms (EIC) from HLM experiment of ADB‐P‐5Br‐INACA and reference substances, showing metabolites in blue and references in black.

The amide hydrolysis of the terminal amide in the head moiety (A10) was the most prevalent metabolite after 3 h, with a proportion of 19.4% of the total metabolite peak area. There was a clear conformity between the MSMS spectra obtained for A11 and in previous works^10^ (*m/z*: 222 and 293), also metabolite A10 had an apparent retention time match with Presley et al.^10^ (Figure 6). The amide hydrolysis metabolite (A10) also had a longer retention time compared to the parent, as the carboxylic acid is protonated by the acidic mobile phase, thereby the longer retention time. The same was true for metabolite A5, where the exact position of the hydroxy group could be disclosed by use of Buchler et al.^18^ Figure 6 shows the similarity in retention time between A5 and Buchler et al.^18^ The MSMS spectra of metabolite A5 obtained in the HHep and HLM experiments was identical to the spectrum of **18** (Supporting Information).

Amide hydrolysis also occurred together with mono‐hydroxylation, as seen in A6 and A7. The allocation of the hydroxy group was assigned using the same fragments as described before.

HHep experiments conducted on similar SCs, such as ADB‐PINACA, have been shown to produce similar metabolites as those presented for the SCs studied in this work. A study by *Carlier* and coworkers^9^ showed that ADB‐PINACA metabolites were formed via mono‐hydroxylation, dihydroxylation, and carbonyl formation, which are in agreement with the findings in the current study. However, mono‐hydroxylation on the indazole core as well has glucuronidation and carboxylation at the pentyl side chain that was observed for ADB‐PINACA^9^ was not observed in the current study.

### 3.2 ADB‐4en‐P‐5Br‐INACA metabolites formed in HLM and HHep incubations

Nine metabolites of ADB‐4en‐P‐5Br‐INACA were identified in the HHep samples, of which eight showed only phase I metabolism, and one was a phase II metabolite (Table 2). The metabolites eluted between 4.71 and 12.24 min, while the parent drug eluted at 11.23 min. Metabolite IDs and ranks of the metabolites were given in the same way as for ADP‐P‐5Br‐INACA. All relevant data such as metabolite ID rank according to total peak area, biotransformation, chemical formula, retention time, *m/z* of the protonated molecule, minimum and maximum mass error (ppm), identifying fragments, and relative abundance (peak area) compared to the most abundant metabolite as well as the total peak area of all metabolites are available in Table 2. All analytical data such as EIC and MSMS spectra can be found in the Supporting Information.

**TABLE 2:** Results of the metabolism study of ADB‐4en‐P‐5Br‐INACAafter hepatocyte incubation.

Metabolite ID	Metabolite rank	Biotransformation	Chemical formula	Average RT (min)	Average [M + H]^+^	Mass error (ppm)		Peak area (×10^3^)							Identifying fragments [M + H]^+^	% of highest metabolite*	% of all metabolites
					m/z	Min	Max	0.5h_1	0.5h_2	1h_1	1h_2	3h_1	3h_2	Total	m/z		
P‐B	n/a	n/a	C19 H25 Br N4 O2	11.23	421.1234	−1.3	0.9	4,674	4782	2528	2252	2190	1952	18378	291.0, 222.9	n/a	n/a
B1	5	Dihydrodiol (T) + mono‐hydroxylation (H)	C19 H27 Br N4 O5	4.71	471.1232	−2.4	0.6	66	72	121	134	193	182	769	325.0, 307.0, 279.0, 222.9, 85.0, 67.0	33.0	8.2
B2	9	Trihydroxylation (2H + 1 T)	C19 H25 Br N4 O5	4.90	469.1074	−2.7	0.0	45	42	74	83	92	97	433	307.0, 279.0, 196.9, 85.0	18.6	4.6
B3	3	Dehydrogenation (H) + trihydroxylation (2H + 1 T)	C19 H23 Br N4 O5	5.86	467.0922	−2.1	−0.1	97	91	140	193	232	220	974	307.0, 279.0, 222.9, 85.0, 67.0	41.9	10.3
B4	10	Mono‐hydroxylation (H) + glucuronidation	C25 H33 Br N4 O9	6.14	613.1497	−1.8	−0.9	n.d.	n.d.	48	51	100	91	291	307.0, 67.0	12.5	3.1
B5*	1	Dihydrodiol (T)	C19 H27 Br N4 O4	6.31	455.1286	−2.3	0.0	272	271	390	403	512	477	2,326	325.0, 307.0, 279.0, 222.9, 85.0, 67.0	100	24.7
B6*	6	Amide hydrolysis (H) + dihydrodiol (T)	C19 H26 Br N3 O5	7.31	456.1120	−4.3	0.2	22	21	76	83	179	183	563	325.0, 307.0, 279.0, 222.9, 85.0, 67.0	24.2	6.0
B7a*	7	Mono‐hydroxylation (T)	C19 H25 Br N4 O3	8.12	437.1180	−1.7	−0.1	105	102	88	98	75	76	543	307.0, 222.9	23.4	5.8
B7b*	8	Mono‐hydroxylation (T)	C19 H25 Br N4 O3	8.25	437.1176	−2.7	−0.7	97	91	86	88	73	80	516	307.0, 222.9	22.2	5.5
B8	4	Mono‐hydroxylation (H)	C19 H25 Br N4 O3	9.15	437.1176	−1.8	0.0	116	127	108	135	147	136	769	291.0, 222.9, 69.0	33.1	8.2
B9*	2	Amide hydrolysis (H)	C19 H24 Br N3 O3	12.24	422.1074	−2.7	0.3	179	180	333	346	624	581	2242	291.0, 222.9, 69.0	96.4	23.8

*Note*: Liquid chromatography–quadrupole time‐of‐flight mass spectrometry (LC‐QTOF‐MS) analysis and data selection including the following data: metabolite ID, metabolite rank, biotransformation, chemical formula, average retention time (min), average m/z of [M + H]^+^ ions, min and max mass errors (ppm), peak areas (×10^3^) of the 0.5, 1, and 3 h samples, as well as the total area, the identifying fragments (m/z), and the relative abundance (%) that is given in relation to the total area of the highest metabolite (B5) as well as the total area of all metabolites. B7 is split into a and b because the additional enantiomeric center creates a diastereomer that is separated by the chromatographic system. The location of a particular biotransformation is indicated by H = head‐group or T = tail‐group. Asterisk = identified using reference substance. More detailed structure information is provided in Figure 7.

Abbreviations: n/a, not applicable; n.d. not detected.

**FIGURE 7:**
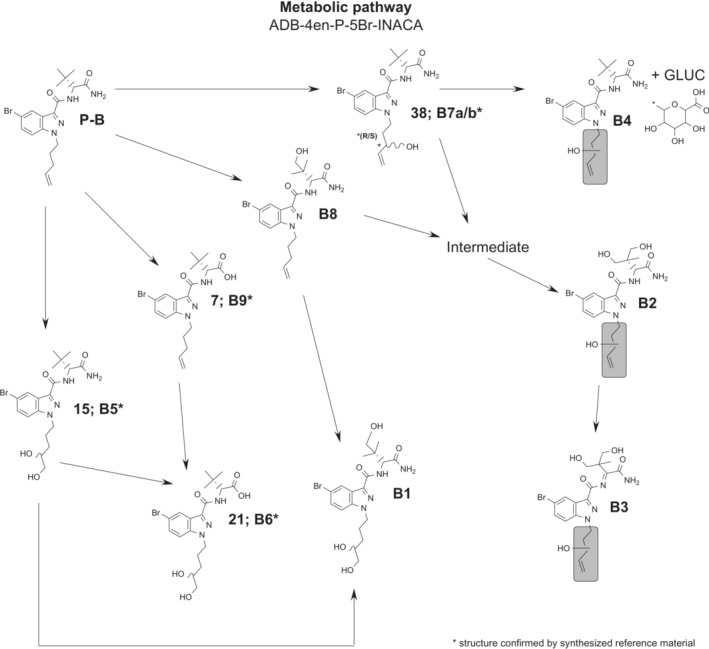
Proposed metabolic pathway of ADB‐4en‐P‐5Br‐INACA. **B7** is named “a/b” because the metabolite is split into two peaks that was confirmed by the analysis of the reference substance that showed an identical peak splitting, caused by the additional chiral centre formed by the hydroxy group of the metabolite at the 3′‐position of the pentenyl chain, creating a diastereomer (enantiomeric centers are marked with *). The “intermediate” placeholder before B2 in the pathway is most likely a dihydroxylation but was not found in the analysis or was excluded during data selection due to missing MSMS fragmentation spectra. Gray areas mark potential positions for hydroxylation.

**FIGURE 8:**
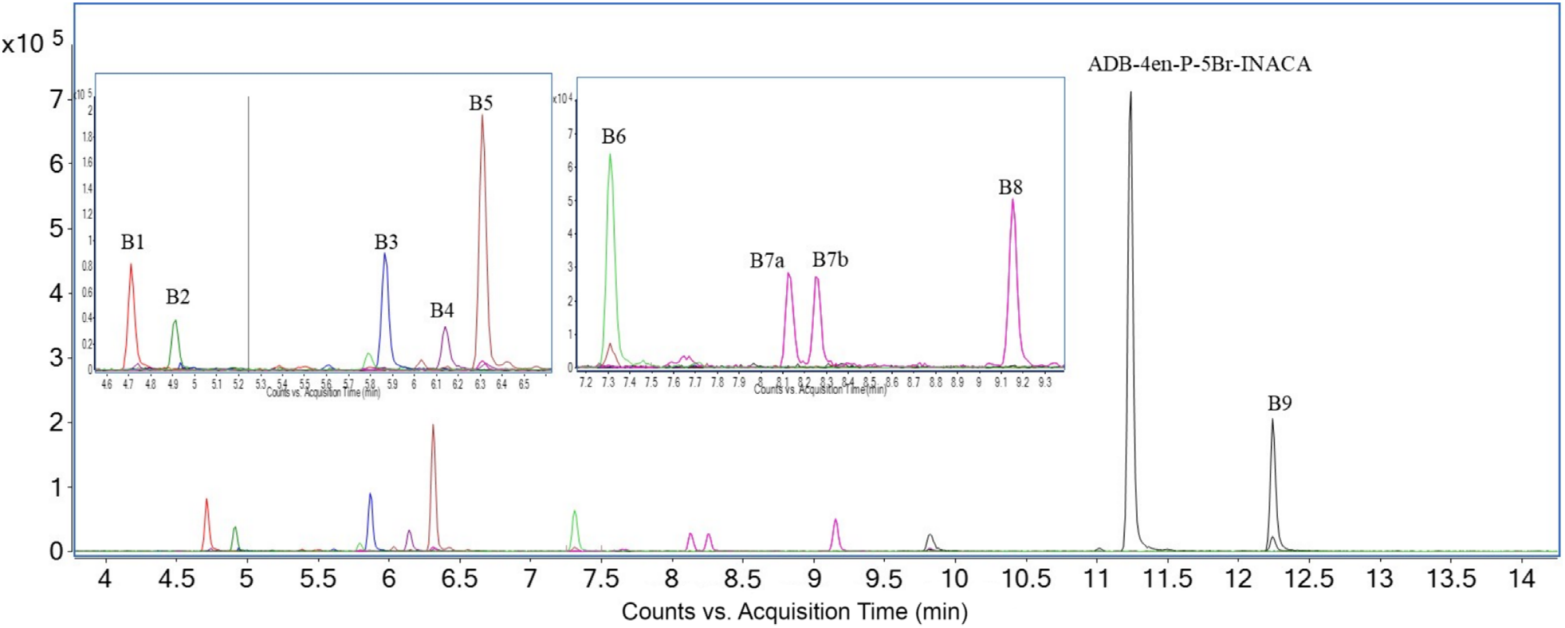
Overlaid extracted ion chromatograms (EIC) from human hepatocytes (HHep) of ADB‐4en‐P‐5Br‐INACA and all its identified metabolites marked with the corresponding metabolite IDs.

The observed biotransformations included phase I reactions of dihydrodiol formation, amide hydrolysis, hydroxylation, dehydrogenation, and combinations thereof. Glucuronidation as a phase II reaction, giving one metabolite, was also observed (Table 2). The most abundant metabolite was a dihydrodiol, that is, metabolite B5. Analysis of Rautio et al.^15^ could reveal that the dihydrodiol were located at the terminal positions of the pentenyl side chain. Amide hydrolysis gave rise to the second most abundant metabolite (B9), which structure could be confirmed through analysis of Carlier et al.^7^ Trihydroxylation followed by dehydrogenation gave rise to the third most abundant metabolite (B3). In total, metabolites B5, B9, and B3 accounted for 58.8% of the total EIC peak area of all metabolites. The proposed metabolic pathway is shown in Figure 7, and overlayed EICs of the metabolites identified in the HHep experiments are shown in Figure 8.

**FIGURE 9:**
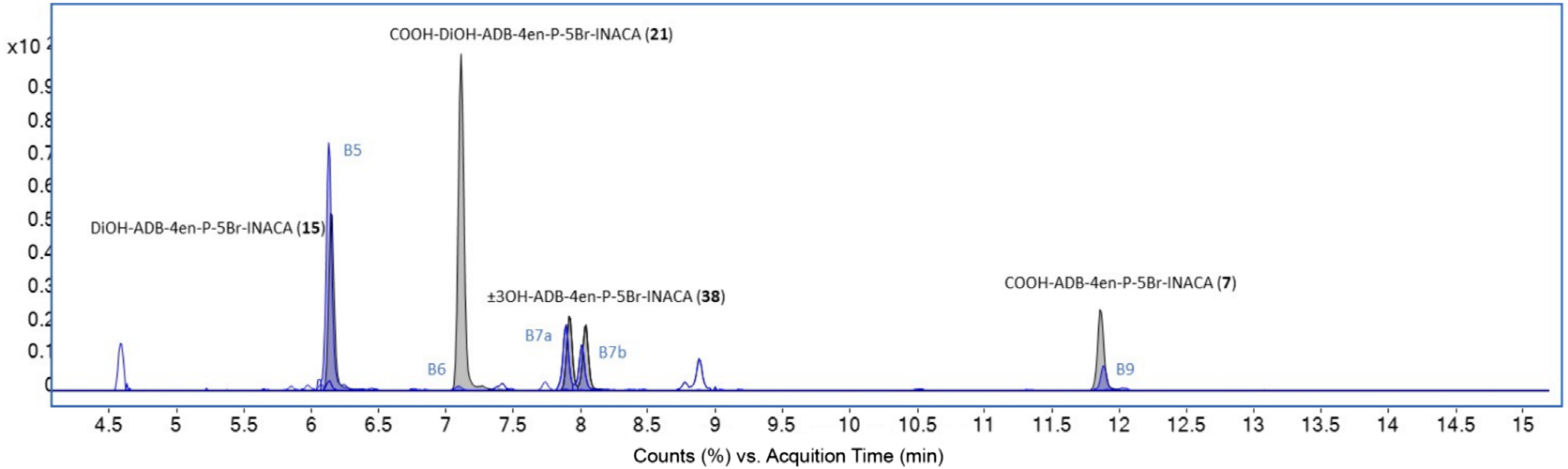
Overlayed extracted ion chromatograms (EIC) from HLM experiment of ADB‐4en‐P‐5Br‐INACA and reference substances. Metabolites are in blue and references in black.

Dihydrodiol formation was observed in three of the nine identified metabolites (B1, B5, and B6). Identification was mainly made by the *m/z* 325 and 307 fragments that represent the pentenyl indazole fragment with dihydrodiol and the same fragment after neutral water loss, respectively. The presence of fragment *m/z* 223 indicated that the dihydrodiol was not located on the indazole core. These fragments were formed for all three metabolites. The structures of metabolites B5 and B6 were confirmed through analysis of Rautio et al.^15^, ^21^, respectively; both MSMS spectra (Supporting Information) and retention times (Figure 9) were matching. In theory, Rautio et al.^15^, ^21^ should be obtained as pairs of diastereomers. However, NMR and QTOF analysis could not confirm that this was the case for these substances (Supporting Information).

Amide hydrolysis occurred for two metabolites, that is, B9 and the previously discussed B6. Similar to the results of ADB‐P‐5Br‐INACA, there were no fragments that could reveal amide hydrolysis. However, considering the observed *m/z* of M + H for B6 and B9, the amide hydrolysis can only have taken place at the terminal position of the head moiety, as it agrees with the theoretical M + H for B6 and B9. Amide hydrolysis of the secondary amide would lead to lower observed *m/z* of M + H. Just like B6, the structure of metabolite B9 could be confirmed through QTOF analysis that resulted in matching MSMS spectra (Supporting Information) and retention times (Figure 9) with Carlier et al.^7^

Six out of nine identified metabolites were identified as hydroxylated metabolites (B1, B2, B3, B4, B7a, B7b, and B8). B7a and B7b could be identified as a diastereomeric pair by comparing their retention time (Figure 9) and MSMS spectra (Supporting Information) to those of reference **38**. Note that reference **38** was also a diastereomeric pair (Figure 3; **38a** and **38b**) that was a result of the Grignard reaction used in the synthesis. The identifying fragments for mono‐hydroxylation on the pentenyl side chain were *m/z* 307 and 85. Hydroxy groups located on the *tert*‐butyl moiety were identified through the presence of fragment *m/z* 291 that indicates the absence of hydroxy groups in this fragment. Metabolite B3 was likely formed through trihydroxylation followed by dehydrogenation. However, the exact locations of the hydroxy group and the double bond could not be determined due to the absence of reference substance, though the presence of fragments *m/z* 307 and 85 suggest that there is a mono‐hydroxylation on the tail and that the dehydrogenation had occurred at the *tert*‐butyl moiety. However, there is a possibility that metabolite B3 has a carboxylic acid located at the *tert*‐butyl moiety together with a hydroxylated tail, instead of being a dehydrogenated tri‐hydroxy metabolite. However, this could not be resolved by the MSMS spectrum of B3, and it is believed that B3 originates from metabolite B2 for this reason, metabolite B3 was identified as a dehydrogenated tri‐hydroxy metabolite. Metabolite B4 was the only phase II metabolite observed in this study. B4 was a monohydroxy glucuronide metabolite with the hydroxy group located at the pentenyl side chain that could be identified by the *m/z* 307 and 85 fragments.

The metabolism of ADB‐4en‐PINACA has previously been studied with HHep.^19^ ADB‐4en‐PINACA is structurally similar to ADB‐4en‐P‐5Br‐INACA, and their metabolic pathways are therefore relevant for comparison; the only difference is the brominated indazole core in ADB‐4en‐P‐5Br‐INACA. The two SCs did both produce metabolites through dihydrodiol formation, amide hydrolysis, and mono‐hydroxylation. However, mono‐hydroxylation on the indazole core was observed for ADB‐4en‐PINACA^19^ that was not observed for ADB‐4en‐P‐5Br‐INACA in the current work. This was potentially due to the presence of bromide that may introduce steric hindrance or electron withdrawing effects that prevent mono‐hydroxylation.

## 4 LIMITATIONS

There are some limitations of the LC‐QTOF‐MS method that were applied in this metabolic study. Although LC‐QTOF‐MS is a sensitive and selective technique, the different ionization efficiencies of various metabolites mean that also the response factors of these metabolites will vary. In practice, this means that a large peak area does not necessarily mean that a metabolite is more abundant than another metabolite with smaller peak area. Hence, ranking of metabolites according to their abundance based on their peak area might not be fully correct. Moreover, the metabolites identified in the HHep and HLM incubations have not yet been confirmed through the analysis of authentic urine samples that would strengthen the in vitro data. Nonetheless, the data corresponds well with previously conducted metabolic studies on similar compounds, that is, ADB‐PINACA and ADB‐4en‐PINACA.

## 5 CONCLUSION

Ten metabolites of ADB‐P‐5Br‐INACA and nine metabolites of ADB‐4en‐P‐5Br‐INACA were detected in metabolism studies using HHep. Metabolic pathways for the two substances have been proposed. These new SCs showed similar metabolic pathways as other previously studied SCs, and common biotransformations were hydroxylation, dihydrodiol formation, carbonyl formation, terminal amide hydrolysis, dehydrogenation, and glucuronidation. The locations of hydroxy groups were spread between tail and head moieties. Due to their structural uniqueness and high abundance, the recommended biomarkers for ADB‐P‐5Br‐INACA and ADB‐4en‐P‐5Br‐INACA would be Buchler et al.^18^ (A5) and Rautio et al.^15^ (B5), respectively.

## CONFLICT OF INTEREST STATEMENT

Authors have no conflict of interest to report.

## Supporting information


**Data S1.** Supporting Information.

**Data S2.** Supporting Information.

## Data Availability

The data for this article is available in the article and the online Supporting Information.
