# Supplementary material for: In vitro metabolism study of ADB‐P‐5Br‐INACA and ADB‐4en‐P‐5Br‐INACA using human hepatocytes, liver microsomes, and in‐house synthesized references
Source: Drug Test Anal. 2024 Jul 23;17(5):701–12. doi: 10.1002/dta.3773 (PMC12012409; doi:10.1002/dta.3773)

Supporting information

Synthesis

General information

HPLC-MS was performed on a Waters system with SQ detector 2, 2 x 515 HPLC pump, 2998 photodiode array detector, 2424 ELD detector, a C18 column (XBridge 3.5 µm, 4.6x50 mm, pore size 130 Å). Mobile phase: water phase A: acetonitrile:water 5:95, with 10 mM NH_4_OAc; organic phase B: acetonitrile:water 90:10, with 10 mM NH_4_OAc. A 4 min gradient A:B, 80:20 🡪 0:100, injection volume 20 µL, 1.5 mL/min. ^1^H, and ^13^C-NMR spectra were recorded on a Bruker 500/126 MHz instrument (25 °C, CDCl_3_, (CD_3_)*_2S_*O or CD_3_OD).

Experimental Section

2-(3-bromopropyl)oxirane (**2**)

In a 50 mL round bottomed flask, 5-Bromo-1-pentene (**1**) (0.397 mL, 0.034 mol, 1 eq.) was dissolved in DCM (12 mL) and mCPBA (meta-chloroperoxybenzoic acid) (3.474 g, 0.05 mol, 1.5 eq.) was added portion wise. The white suspension was stirred at rt for 21 h, then quenched with saturated NaHSO_3_ solution (10 mL). The white solid was filtered off, the liquid phase was basified with saturated NaHCO_3_ solution (30 mL) and the aqueous layer was extracted with DCM (5 x 10 mL). The organic phases were combined, washed with brine (15 mL), dried over MgSO_4_, filtered and concentrated giving 2-(3-bromopropyl)oxirane (**2**) as a light brown oil (0.310 g, 56 %). ^1^H-NMR (CDCl_3_, 500 MHz): δ 3.40-3.50 (m, 2H), 2.94-2.95 (m, 1H), 2.77 (t, *J* = 4.4 Hz, 1H), 2.50-2.52 (m, 1H), 1.97-2.09 (m, 2H), 1.56-1.53 (m, 2H).

Methyl (*2S*)-2-[(5-bromo-1*H*-indazole-3-carbonyl)amino]-3,3-dimethylbutanoate (**5**)

In a 100 mL round bottomed flask, 5-bromo-1*H*-indazole-3-carboxylic acid (**3**) (0.250 g, 1 mmol, 1 eq.) was dissolved in acetonitrile (10 mL). Methyl (*2S*)-2-amino-3,3-dimethyl-butanoate (**4**) (0.226 g, 1.2 mmol, 1.2 eq.), TBTU (O-(Benzotriazol-1-yl)-N,N,N’,N’-tetramethyluronium tetrafluoro borate) (0.366 g, 1.1 mmol, 1.1 eq.) and Et_3_N (triethylamine) (0.431 mL, 3.1 mmol, 3 eq.) were added. Due to poor solubility, acetonitrile (10 mL) followed by DMF (3 mL) were added and the resulting light-yellow suspension was stirred at 60 °C for 3 h then at rt 21 h. LC/MS showed still starting material left so TBTU (100 mg, 0.3 mmol, 0.3 eq.) was added and the reaction was stirred at 60 °C for 2 h. LC/MS showed full conversion. The reaction mixture was evaporated, resuspended in approx. 5 mL of a 1:1 EtOAc:heptane solution, filtered and the filter cake was washed. The solvents was removed by in vacuo and the crude was purified by SiO_2_-column (EtOAc:heptane 1:1) to give methyl (*2S*)-2-[(5-bromo-1*H*-indazole-3-carbonyl)amino]-3,3-dimethylbutanoate (**5**) as a white solid (225 mg, 59 % yield). ^1^H-NMR (CDCl_3_, 500 MHz): δ 10.46 (br s, 1H), 8.56 (s, 1H), 7.69 (d, *J* = 9.0 Hz, 1H), 7.52 (d, *J* = 8.8 Hz 1H), 7.40 (d, *J* = 8.8 Hz, 1H), 4.72 (d, *J* = 9.6 Hz, 1H), 3.79 (s, 3H), 1.10 (s, 9H)

(*2S*)-2-[(5-bromo-1-pent-4-enyl-indazole-3-carbonyl)amino]-3,3-dimethyl-butanoic acid (**7**)

In a 5 mL microwave vial, methyl (*2S*)-2-[(5-bromo-1H-indazole-3-carbonyl)amino]-3,3-dimethylbutanoate (**5**) (0.040 g, 0.11 mmol, 1 eq.) was dissolved in DMF (2 mL). K_2_CO_3_ (0.030 g, 0.22 mmol, 2 eq.) was added and the resulting white suspension was stirred at rt for 15 min. 5-Bromopent-1-ene (**1**) (0.017 mL, 0.14 mmol, 1.3 eq.) was added and the resulting white suspension was stirred at rt for 21 h. To the above reaction mixture, 1 M aqueous NaOH solution (0.55 mL, 0.55 mmol, 5 eq.) was added, the resulting mixture was stirred at rt for 5 h. The reaction mixture was poured into 1 M aqueous HCl (5 mL) and the aqueous layer was extracted with DCM (3x8 mL). The combined organic phases were evaporated and the crude oil was dissolved in methanol. The resulting solution was purified by preparative HPLC giving (*2S*)-2-[(5-bromo-1-pent-4-enyl-indazole-3-carbonyl)amino]-3,3-dimethylbutanoic acid (**7**) as an orange oil (41 mg, 89 % yield). ^1^H-NMR (CDCl_3_, 500 MHz): δ 8.48 (s, 1H), 7.49 (d, *J* = 8.7 Hz, 1H), 7.42 (d, *J* = 8.9 Hz, 1H), 7.23 (d, *J* = 8.9 Hz, 1H), 5.77-5.82 (m, 1H), 5.04 (d, *J* = 5.4, 1H), 5.02 (s, 1H), 4.59 (d, *J* = 7.7 Hz, 1H), 4.36 (t, *J* = 6.7 Hz, 2H), 2.02-2.08 (m, 4H), 1.11 (s, 9H). ^13^C-NMR (CDCl_3_, 126 MHz): δ 175.6, 162.2, 139.7, 137.0, 136.2, 130.0, 125.3, 124.4, 116.24, 116.21, 110.8, 60.8, 49.0, 34.8, 30.8, 28.8, 26.9. HRMS [M+H]: *m/z* 422.1083, 1.94 ppm.

(*2S*)-2-[(5-bromo-1-pentyl-indazole-3-carbonyl)amino]-3,3-dimethyl-butanoic acid (**10**)

In a 5 mL round bottomed flask, methyl (*2S*)-2-[(5-bromo-1H-indazole-3-carbonyl)amino]-3,3-dimethylbutanoate (**5**) (0.040 g, 0.11 mmol, 1 eq.) was dissolved in DMF (2 mL). K_2_CO_3_ (0.030 g, 0.22 mmol, 2 eq.) was added and the resulting white suspension was stirred at rt for 15 min. 1-Bromopentane (**8**) (0.018 mL, 0.14 mmol, 1.3 eq.) was added and the resulting light white suspension was stirred at rt 21 h. To the above reaction mixture, 1 M aqueous NaOH solution (0.55 mL, 0.55 mmol, 5 eq.) was added. The resulting mixture was stirred at rt for 21 h. The reaction mixture was then poured into 1 M aqueous HCl (5 mL) and the aqueous layer was extracted with DCM (3x 8 mL). The combined organic phases were evaporated and the crude oil was dissolved in methanol. The resulting solution was purified by preparative HPLC giving (*2S*)-2-[(5-bromo-1-pentyl-indazole-3-carbonyl)amino]-3,3-dimethyl-butanoic acid (**10**) as a white foam (29 mg, 63 % yield) ^1^H-NMR (CDCl_3_, 500 MHz): δ 8.49 (s, 1H), 7.49 (d, 1H, *J* = 9.1 Hz), 7.43 (d, 1H, *J* = 8.9 Hz), 7.25 (d, 1H, *J* = 8.5 Hz), 4.61 (d, 1H, *J* = 9.05 Hz), 4.35 (t, 2H, *J* = 7.2 Hz), 1.92 (quin, 2H, *J* = 7.3 Hz), 1.25-1.38 (m, 4H), 1.11 (s, 9H), 0.88 (t, 3H, *J* = 7.1 Hz). ^13^C-NMR (CDCl_3_, 126 MHz): δ 175.5, 162.3, 139.6, 136.1, 130.0, 125.4, 124.4, 116.2, 110.8, 60.3, 49.8, 34.8, 29.5, 29.0, 26.9, 22.3, 14.0. HRMS [M+H]: *m/z* 424.1228, -0.57 ppm.

5-bromo-*N*-[(*1S*)-1-carbamoyl-2,2-dimethyl-propyl]-1*H*-indazole-3-carboxamide (**12**)

In a 100 mL round bottomed flask, 5-bromo-1H-indazole-3-carboxylic acid (**3**) (0.300 g, 1.2 mmol, 1 eq.) was dissolved in acetonitrile (20 mL) and DMF (4 mL). (*2S*)-2-amino-3,3-dimethylbutanamide (**11**) (0.249 g, 1.5 mmol, 1.2 eq.), TBTU (0.520 g, 1.6 mmol, 1.3 eq.) and Et3N (0.518 mL, 3.7 mmol, 3 eq.) were added. The resulting light-yellow suspension was stirred at 60 °C for 4 h. LC-MS showed still starting material left, thus TBTU (120 mg, 0.4 mmol, 0.3 eq.) was added and the reaction was stirred at 60 °C for 1 h. The solvent was evaporated and the obtained crude was purified with a SiO2-column (EtOAc:heptane 4:1) giving 5-bromo-*N*-[(*1S*)-1-carbamoyl-2,2-dimethyl-propyl]-1*H*-indazole-3-carboxamide **12** (300 mg, 68 % yield). ^1^H-NMR (Acetone-d6, 500 MHz): δ 12.9 (s, 1H), 8.47 (d, J = 1.2 Hz, 1H), 7.72 (d, J = 8.9 Hz, 1H), 7.66 (d, *J* = 8.6 Hz, 1H), 7.54-7.56 (dd, *J* = 8.9, 1.7 Hz, 1H), 7.20 (br s, 1H), 6.62 (br s, 1H), 4.58 (d, 1H, J=9.5 Hz), 1.09 (s, 9H).

5-bromo-*N*-[(*1S*)-1-carbamoyl-2,2-dimethyl-propyl]-1-[3-(oxiran-2-yl)propyl]indazole-3-carboxamide (**13**)

In a 5 mL microwave vial, 5-bromo-*N*-[(*1S*)-1-carbamoyl-2,2-dimethyl-propyl]-1*H*-indazole-3-carboxamide (**12**) (0.074 g, 0.21 mmol, 1 eq.) was dissolved in DMF (3.5 mL). K_2_CO_3_ (0.058 g, 0.42 mmol, 2 eq.) was added and the resulting suspension was stirred at rt for 15 min. 2-(3-bromopropyl)oxirane (**2**) (0.045 mg, 0.27 mmol, 1.3 eq.) was added and the resulting suspension was stirred at rt for 21 h. The reaction mixture was poured into water (10 mL) and the aqueous layer was extracted with DCM (3x 10 mL). The combined organic phases were evaporated and the obtained crude oil was dissolved in methanol. The resulting solution was purified by preparative HPLC giving 5-bromo-*N*-[(*1S*)-1-carbamoyl-2,2-dimethyl-propyl]-1-[3-(oxiran-2-yl)propyl]indazole-3-carboxamide (**13**) as colorless oil (24.9 mg, 27 % yield). ^1^H-NMR (CDCl_3_, 500 MHz): δ 8.47 (s, 1H), 7.64 (d, *J* = 9.4 Hz, 1H), 7.48 (d, *J* = 8.8 Hz, 1H), 7.33 (d, *J* = 8.9 Hz, 1H,), 6.26 (br s, 1H), 5.69 (br s, 1H), 4.59 (d, J = 9.5 Hz, 1H), 4.42-4.46 (m, 2H), 2.94-2.97 (m, 1H), 2.75-2.76 (m, 1H), 2.45-2.48 (m, 1H), 2.09-2.15 (m, 2H), 1.68-1.73 (m, 1H), 1.40-1.41 (m, 1H), 1.14 (s, 9H)

5-bromo-*N*-[(*1S*)-1-carbamoyl-2,2-dimethyl-propyl]-1-(4-hydroxypentyl)indazole-3-carboxamide (**14**)

In a 5 mL microwave vial, 5-bromo-*N*-[(*1S*)-1-carbamoyl-2,2-dimethyl-propyl]-1-[3-(oxiran-2-yl)propyl]indazole-3-carboxamide (**13**) (0.024 g, 0.05 mmol, 1 eq.) was dissolved in 2-propanol (1.4 mL). NaBH_4_ (0.0042 g, 0.11 mmol, 2 eq.) was added and the resulting suspension was stirred at 60°C. After 4 h LC-MS still showed that starting material was present, so more NaBH_4_ (0.0010 g, 0.03 mmol, 0.5 eq.) was added and the mixture was stirred at 60 °C for another 2 h, then at rt for 21 h. The reaction mixture was acidified with 1 M aqueous HCl (2 mL) then extracted with DCM (3x10 mL). The combined organic layers were evaporated, dissolved in methanol and purified by preparative HPLC to give 5-bromo-*N*-[(*1S*)-1-carbamoyl-2,2-dimethyl-propyl]-1-(4-hydroxypentyl)indazole-3-carboxamide (**14**) as colorless oil (14.7 mg, 61 % yield). ^1^H-NMR (CDCl_3_, 500 MHz): δ 8.47 (d, 1H, *J* = 1.2 Hz), 7.64 (d, *J* = 9.4 Haz, 1H), 7.48 (dd, *J* = 1.7, 8.9 Hz, 1H), 7.31 (d, *J* = 8.9 Hz, 1H), 6.19 (br s, 1H), 5.75 (br s, 1H), 4.57 (d, *J* = 9.5 Hz, 1H), 4.40 (t, *J* = 7.2 Hz, 2H), 3.82-3.88 (m, 1H), 2.01-2.11 (m, 3H), 1.44-1.49 (m, 2H), 1.19 (dd, *J* = 2.4, 6.1 Hz), 1.14 (s, 9H). ^13^C-NMR (CDCl_3_, 126 MHz): δ 172.9, 162.3, 139.7, 136.2, 130.2, 125.3, 124.4, 116.3, 110.9, 67.7, 59.9, 49.7, 36.1, 34.8, 26.9, 26.0, 23.9. HRMS [M+H]: *m/z* 439.1349, -2.27 ppm.

5-bromo-N-[(*1S*)-1-carbamoyl-2,2-dimethyl-propyl]-1-(4,5-dihydroxypentyl)indazole-3-carboxamide (**15**)

In a 0.2-0.5 mL microwave vial 5-bromo-N-[(*1S*)-1-carbamoyl-2,2-dimethyl-propyl]-1-[3-(oxiran-2-yl)propyl]indazole-3-carboxamide (**13**) (5 mg, 0.011 mmol, 1 eq.) was dissolved in THF (tetrahydrofuran) (190 µl), a solution of TFA (trifluoroacetic acid) in water (0.1708 M, 17 µl, 0.0029 mmol, 0.26 eq.) was added. The microwave vial was sealed and left to stir for 40 min at 120 °C with normal absorption. LC-MS showed that starting material was still present, so the reaction was stirred at 120 °C for another 30 min. LC-MS showed no increase in conversion thus, more of triflic acid solution (34 µl, 0.5 eq.) was added and the microwave vial was sealed again and left to stir for 40 min at 120 °C with normal absorption. The crude was purified by preparative HPLC to give a diastereomeric mixture of 5-bromo-N-[(*1S*)-1-carbamoyl-2,2-dimethyl-propyl]-1-(4,5-dihydroxypentyl)indazole-3-carboxamide (**15**) as colorless oil (4 mg, 80 % yield). ^1^H-NMR (CDCl_3_, 500 MHz): δ 8.43-8.44 (m, 1H), 7.69 (t, *J =* Hz, 1H), 7.46 (td, *J* = 1.5, 9.0 Hz, 1H), 7.29 (dd, *J* = 2.7, 8.9 Hz, 1H), 6.32-6.34 (m, 1H), 5.97-6.00 (m, 1H), 4.55 (d, *J* = 9.5 Hz 1H), 4.41 (q, 2H, *J* = 6.9 Hz), 3.74-3.81 (m, 1H), 3.57-3.63 (m, 1H), 3.37-3.43 (m, 1H), 2.90 (br s, 1H), 1.99-2.17 (m, 2H), 1.68 (br s, 1H), 1.39-1.47 (m, 1H), 1.28-1.24 (m, 1H), 1.12 (s, 9H), 0.83-0.99 (m, 1H). ^13^C-NMR (CDCl_3_, 126 MHz): δ 173.2, 162.2, 139.6, 136.1, 130.2, 125.2, 124.3, 116.3, 110.9, 71.6, 66.8, 59.9, 49.4, 34.9, 29.9, 26.8, 25.6. HRMS [M+H]: *m/z* 455.1397, -1.9 ppm.

5-bromo-*N*-[(*1S*)-1-carbamoyl-2,2-dimethyl-propyl]-1-(5-hydroxypentyl)indazole-3-carboxamide (**18**)

In a 5 mL microwave vial, 5-bromo-*N*-[(*1S*)-1-carbamoyl-2,2-dimethyl-propyl]-1*H*-indazole-3-carboxamide (**12**) (0.040 g, 0.11 mmol, 1 eq.) was dissolved in DMF (2 mL). K_2_CO_3_ (0.031 g, 0.23 mmol, 2 eq.) was added and the resulting suspension was stirred at rt for 15 min. Methyl-5-bromo-pentylacetate (**16**) (0.025 mL, 0.15 mmol, 1.3 eq.) was added and the resulting suspension was stirred at rt 21 h. To the reaction mixture 1 M aqueous NaOH solution (0.57 mL, 0.57 mmol, 5 eq.) was added and stirred at rt for 4 h. The reaction mixture was poured into water (5 mL) and the aqueous layer was extracted with DCM (3x 8 mL). The combined organic phases were evaporated and the crude oil was dissolved in methanol. The resulting solution was purified by preparative HPLC giving 5-bromo-*N*-[(*1S*)-1-carbamoyl-2,2-dimethyl-propyl]-1-(5-hydroxypentyl)indazole-3-carboxamide (**18**) as a colorless oil (21.7 mg, 40 % yield). ^1^H-NMR (CDCl_3_, 500 MHz): δ 8.50 (s, 1H), 7.71 (d, *J* = 9.5 Hz, 1H), 7.50 (d, *J* = 9.0 Hz, 1H), 7.32 (d, *J* = 9.0 Hz, 1H), 6.19 (br s, 1H), 5.75 (br s, 1H), 4.59 (d, *J* = 9.5 Hz, 1H), 4.41 (t, *J* = 6.9 Hz, 2H), 3.69-3.63 (m, 2H), 2.00 (quin, *J* = 7.3 Hz, 2H), 1.66-1.63 (m, 2H), 1.46-1.40 (m, 2H), 1.16 (s, 9H). ^13^C-NMR (CDCl_3_, 126 MHz): δ 172.8, 162.2, 139.5, 136.0, 130.0, 125.1, 124.3, 116.2, 110.7, 62.4, 59.7, 49.4, 34.7, 31.1, 29.2, 26.7, 22.9. HRMS [M+H]: *m/z* 439.1339, -2.03 ppm.

5-bromo-*N*-[(*1S*)-1-carbamoyl-2,2-dimethyl-propyl]-1-[3-(oxiran-2-yl)propyl]indazole-3-carboxamide (**19**)

In a 25 mL round bottomed flask, methyl (*2S*)-2-[(5-bromo-1*H*-indazole-3-carbonyl)amino]-3,3-dimethylbutanoate (**5**) (0.080 g, 0.22 mmol, 1 eq.) was dissolved in DMF (1.5 mL). K_2_CO_3_ (0.060 g, 0.43 mmol, 2 eq.) was added and the resulting suspension was stirred at rt for 15 min. 2-(3-bromopropyl)oxirane (**2**) (0.047 g, 0.28 mmol, 1.3 eq.) was added and the resulting suspension was stirred at rt 21 h. The reaction mixture was filtered through a syringe filter and purified by preparative HPLC giving 5-bromo-*N*-[(*1S*)-1-carbamoyl-2,2-dimethyl-propyl]-1-[3-(oxiran-2-yl)propyl]indazole-3-carboxamide as colorless oil (**19**) (29 mg, 30 % yield). ^1^H-NMR (CDCl_3_, 500 MHz): δ 8.53 (s, 1H), 7.48 (d, *J* = 9.8 Hz, 2H), 7.32 (d, *J* = 8.9 Hz, 1H), 4.70 (d, *J* = 9.6 Hz, 1H), 4.43-4.48 (m, 2H), 3.76 (s, 3H), 2.94-2.97 (m, 1H), 2.76 (t, *J* = 4.4 Hz, 1H), 2.46-2.49 (m, 1H), 2.10-2.17 (m, 2H), 1.70-1.73 (m, 1H), 1.44-1.49 (m, 1H), 1.08 (s, 9H)

(*2S*)-2-[[5-bromo-1-(4,5-dihydroxypentyl)indazole-3-carbonyl]amino]-3,3-dimethylbutanoic acid (**21**)

In a 5 mL microwave vial, methyl 5-bromo-*N*-[(*1S*)-1-carbamoyl-2,2-dimethyl-propyl]-1-[3-(oxiran-2-yl)propyl]indazole-3-carboxamide (**19**) (0.029 g, 0.064 mmol, 1 eq.) was dissolved in THF (0.5 mL). A solution of triflic acid in water (0.149 M, 162 µl, 0.5 eq.) was added and the microwave vial was sealed and left to stir for 40 min at 120 °C with normal absorption. To the reaction mixture containing the intermediate product **20**, 1M aqueous NaOH solution (268 µl, 5.5 eq.) was added and the resulting mixture was stirred at rt for 3 h. The reaction mixture was poured on 1 M aqueous HCl (5 mL) and the aqueous layer was extracted with DCM (3x 10 mL), the combined organic layers were evaporated. The obtained crude was purified by preparative HPLC giving (*2S*)-2-[[5-bromo-1-(4,5-dihydroxypentyl)indazole-3-carbonyl]amino]-3,3-dimethyl-butanoic acid as a white solid (**21**) (8.6 mg, 28.4 % yield). ^1^H-NMR (Methanol-d4, 500 MHz): δ 8.36 (d, *J* = 1.2 Hz, 1H), 7.62 (d, *J* = 9.0 Hz, 1H), 7.54 (dd, J=1.8, 8.9 Hz, 1H), 4.52-4.55 (m, 3H), 3.59-3.63 (m, 1H), 3.41-3.43 (m, 2H), 2.11-2.18 (m, 1H), 1.99-2.07 (m, 1H), 1.51-1.58 (m, 1H), 1.36-1.43 (m, 1H), 1.10 (s, 9H). ^13^C-NMR (Methanol-d4, 126 MHz): δ 163.6, 141.2, 137.1, 131.1, 125.4, 125.2, 117.1, 113.0, 72.7, 67.2, 50.6, 35.7, 31.4, 27.3, 27.1. HRMS [M+H]: *m/z* 456.1129, -1.00 ppm.

5-bromo-*N*-[(*1S*)-1-carbamoyl-2,2-dimethyl-propyl]-1-(4-oxopentyl)indazole-3-carboxamide (**22**)

In a 5 mL microwave vial, 5-bromo-N-[(*1S*)-1-carbamoyl-2,2-dimethyl-propyl]-1-(4-hydroxypentyl)indazole-3-carboxamide (**14**) (0.0105 g, 0.024 mmol, 1 eq.) was dissolved in DCM (1 mL). Dess-Martin periodinane (0.013 g, 0.030 mmol, 1.25 eq.) was added and the resulting suspension was stirred at rt. After 1 h LC-MS showed that starting material was still present, more Dess-Martin periodinane (0.020 g, 0.048 mmol, 2 eq.) was added. The reaction was stirred at rt for 72 h. The reaction was poured on water (15 mL) and the aqueous layer was extracted with DCM (3x10 mL). The combined organic layers was evaporated and purified by preparative HPLC giving 5-bromo-*N*-[(*1S*)-1-carbamoyl-2,2-dimethyl-propyl]-1-(4-oxopentyl)indazole-3-carboxamide (**22**) as colorless oil (4.2 mg, 40% yield). ^1^H-NMR (CDCl_3_, 500 MHz): δ 8.49 (d, *J* = 1.2 Hz, 1H), 7.64 (d, *J* = 9.3 Hz, 1H), 7.50 (dd, *J* = 1.8, 8.9 Hz, 1H), 7.34 (d, *J* =9.0 Hz, 1H), 6.03 (br s, 1H), 5.56 (br s, 1H), 4.54 (d, *J* = 9.5 Hz, 1H), 4.40-4.44 (m, 2H), 2.39-2.48 (m, 2H), 2.14-2.22 (m, 2H), 2.12 (s, 3H), 1.14 (s, 9H). ^13^C-NMR (CDCl_3_, 126 MHz): δ 207.6, 172.6, 162.2, 139.8, 136.4, 130.3, 125.3, 124.4, 116.4, 110.9, 59.8, 48.5, 39.7, 34.8, 30.2, 26.8, 23.6. HRMS [M+H]: *m/z* 438.1220, -1.81 ppm.

5-bromo-1-(3-hydroxypropyl)indazole-3-carboxylic acid (**33**)

In a 10 mL round bottomed flask, 5-bromo-1*H*-indazole-3-carboxylate (**3**) (0.250 g, 1.04 mmol, 1 eq.) was dissolved in dry DMF (4 mL). NaH (0.103 g, 2.59 mmol, 2.5 eq.) was added at 0 °C and the resulting white suspension was stirred at 0 °C for 15 min. 1-Bromopropanol (0.135 mL, 1.56 mmol, 1.5 eq.) was added and the resulting white suspension was stirred at 0 °C. After 3 h LC-MS showed that starting material was still present. Thus, NaH (0.040 g, 1.04 mmol, 1 eq.) was added and the resulting suspension was stirred at 0 °C for an additional 3 h. LC-MS showed no increase in conversion, thus the reaction mixture was partitioned between 1 M HCl (15 mL) and EtOAc (15 mL). The phases were separated and the aqueous phase was extracted with EtOAc (2x15 mL). The combined organic layers were evaporated and purified by preparative HPLC giving 5-bromo-1-(3-hydroxypropyl)indazole-3-carboxylic acid (**33**) as a grey solid, containing dialyklated and trialkylated product as well as impurities (0.214 g). ^1^H-NMR (MeOH-d4, 500 MHz): δ 8.44 (s, 1H), 7.56-7.52 (m, 1H), 7.51-7.49 (m, 1H), 4.57-4.52 (m, 2H), 3.53 (t, *J* = 6.0 Hz, 2H), 2.34-2.10 (m, 2H).

methyl 5-bromo-1-(3-oxopropyl)indazole-3-carboxylate (**35**)

In a 25 mL round bottomed flask, 5-bromo-1-(3-hydroxypropyl)indazole-3-carboxylic acid (**33**) (0.040 g, 0.13 mmol, 1 eq.) was dissolved in MeOH (0.8 mL) and toluene (2 mL). Trimethyl silyl diazomethane 2 M in hexane (0.10 mL, 0.20 mmol, 1.5 eq.) was added and the resulting yellow solution was stirred at rt for 40 min. The reaction mixture was evaporated to give methyl 5-bromo-1-(3-hydroxypropyl)indazole-3-carboxylate (**34**) as crude material. The crude was redissolved in DCM and Dess-Martin periodinane (0.068 g, 0.16 mmol, 1.2 eq.) was added, the resulting reaction mixture was stirred at rt for 21 h. The reaction was poured on water (10 mL) and the aqueous layer was extracted with DCM (3x 10 mL). The combined organic layers was evaporated and purified by SiO_2_-coloumn (EtOAc:heptane 8:2) giving methyl 5-bromo-1-(3-oxopropyl)indazole-3-carboxylate (**35**) as an orange oil (11.6 mg, 28 % yield), the oil was not pure but used directly in the next step, without further purification.

methyl 5-bromo-1-(3-hydroxypent-4-enyl)indazole-3-carboxylate (**36**)

In a 25 mL round bottomed flask, methyl 5-bromo-1-(3-oxopropyl)indazole-3-carboxylate (**35**) (0.012 g, 0.037 mmol, 1 eq.) was dissolved in dry THF (1 mL). 1 M Vinylmagnesium bromide in THF (0.028 mL, 0.056 mmol, 1.5 eq.) was added and the resulting yellow solution was stirred at rt for 1h. Saturated NH_4_Cl solution (5 mL) was added and the resulting suspension was extracted with EtOAc (3x 5mL). The combined organic layers were evaporated to giving methyl 5-bromo-1-(3-hydroxypent-4-enyl)indazole-3-carboxylate (**36**) (quantitative, 40 mg) as white solid. The crude product was used without further purification in next step.

5-bromo-1-(3-hydroxypent-4-enyl)indazole-3-carboxylic acid (**37**)

In a 25 mL round bottomed flask methyl 5-bromo-1-(3-hydroxypent-4-enyl)indazole-3-carboxylate (**36**) (0.040 g, 0.12 mmol, 1 eq.) was dissolved in THF (1 mL) and MeOH (1 mL). 1 M aqueous NaOH (0.74 mL, 0.74 mmol, 6 eq.) was added and the resulting solution was stirred at rt for 96 h. The reaction mixture was evaporated and suspended in EtOAc (5 mL), the suspension was poured into 1 M HCl (5 mL) and the phases were separated, the aqueous layer was extracted with EtOAc (2x 5 mL) and the combined organic layers were evaporated to give 5-bromo-1-(3-hydroxypent-4-enyl)indazole-3-carboxylic acid (**37**) as orange solid (5 mg, 13% yield). The product was used in next step without further purification.

5-bromo-N-[(*1S*)-1-carbamoyl-2,2-dimethyl-propyl]-1-(3-hydroxypent-4-enyl)indazole-3-carboxamide (**38**)

In a 10 mL round bottomed flask, 5-bromo-1-(3-hydroxypent-4-enyl)indazole-3-carboxylic acid (**37**) (0.005 g, 0.02 mmol, 1 eq.) was dissolved in acetonitrile (1 mL) and DMF (0.22 mL). (*2S*)-2-amino-3,3-dimethyl-butanamide (**11**) (0.003 g, 0.02 mmol, 1.3 eq.) was added, followed by TBTU (0.0064 g, 0.02 mmol, 1.3 eq.) and Et_3_N (0.01 mL, 0.07 mmol, 4.5 eq.). The resulting light-yellow suspension was stirred at rt for 21 h. The reaction mixture was partitioned between EtOAc (5 mL) and H_2_O (5 mL), the phases were separated and the aqueous layer was extracted with EtOAc (2x 5 mL). The combined organic layers were evaporated. The obtained crude product was purified by preparative HPLC giving 5-bromo-*N*-[(*1S*)-1-carbamoyl-2,2-dimethylpropyl]-1-(3-hydroxypent-4-enyl)indazole-3-carboxamide (**38**) as white solid (1.8 mg, 27 %). ^1^H-NMR (CDCl_3_, 500 MHz): δ 8.53 (s, 1H), 7.64 (d, *J* = 9.0 Hz, 1H), 7.50-7.52 (m, 1H), 7.41 (t, *J*  = 8.0 Hz, 1H), 5.87-5.93 (m, 2H), 5.50 (br s, 1H), 5.27 (d, *J* = 17.2 Hz, 1H), 5.16 (d, *J* = 10.4 1H), 4.56-4.62 (m, 1H), 4.50-4.54 (m, 2H), 4.05-4.11 (m, 1H), 2.21-2.28 (m, 1H), 2.07-2.12 (m, 1H), 1.16 (s, 9H). ^13^C-NMR (CDCl_3_, 126 MHz): δ 172.6, 162.2, 140.1, 136.5, 131.0 130.2, 125.2, 124.3, 116.4, 115.7, 111.1, 70.1, 59.6, 45.9, 36.5, 34.8, 26.8. 38a HRMS [M+H]: *m/z* 437.1183, 0.00 ppm. 38b HRMS [M+H]: *m/z* 437.1190, -1.74 ppm.

(*2S*)-2-[(5-bromo-1-pent-4-enyl-in2-(3-bromopropyl)oxirane (**2**)


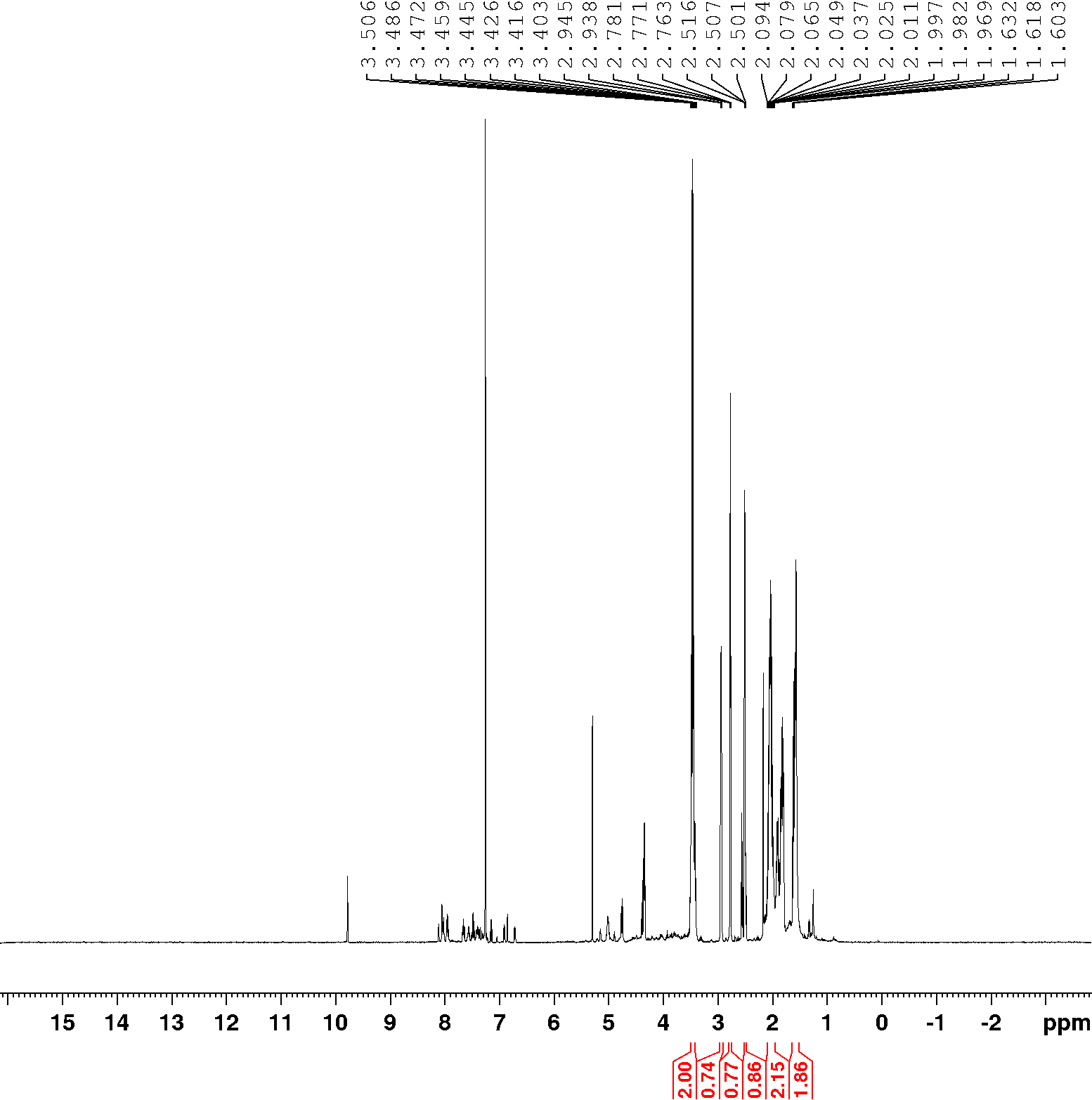


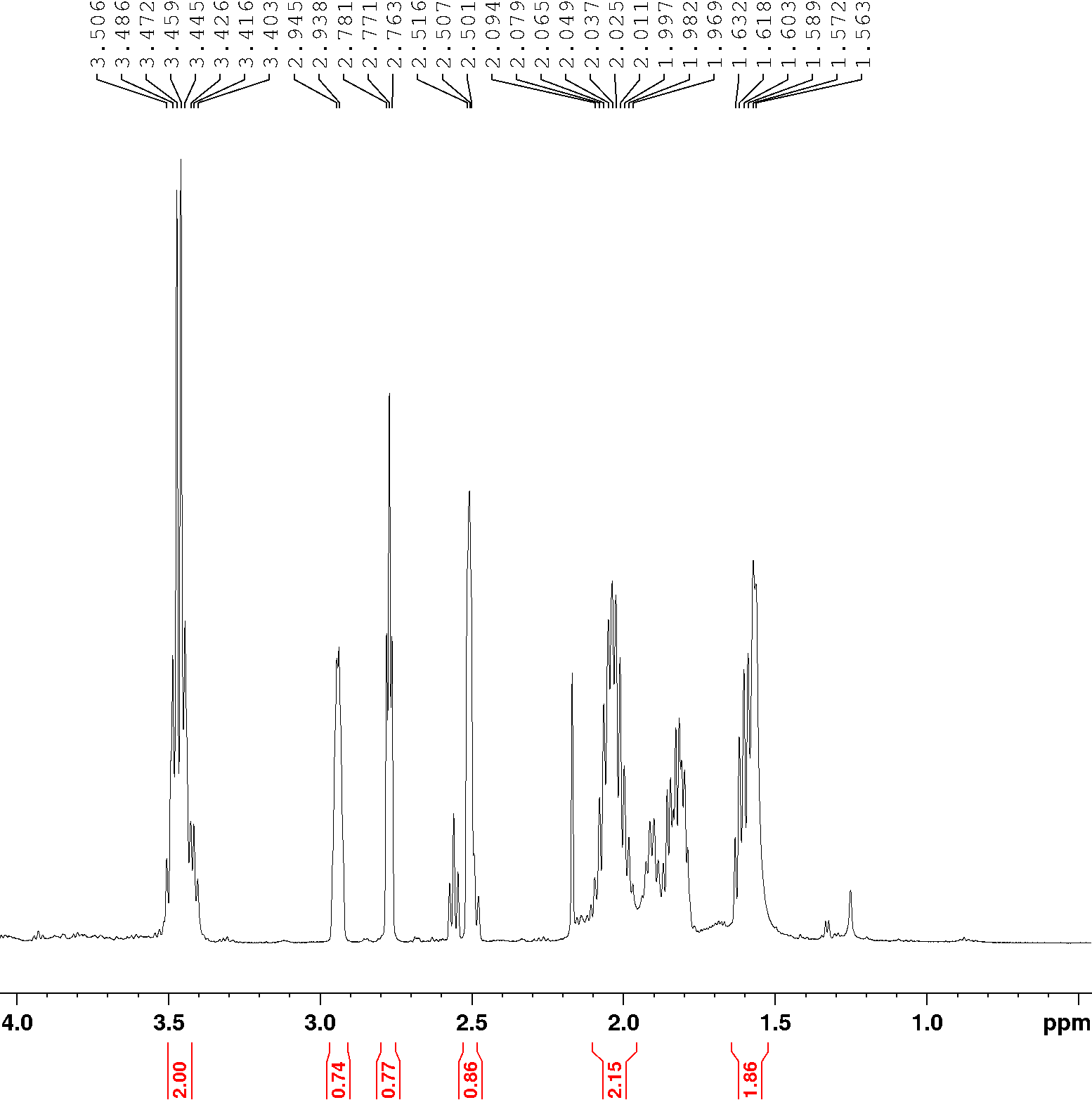


Methyl (*2S*)-2-[(5-bromo-1H-indazole-3-carbonyl)amino]-3,3-dimethylbutanoate (**5**)


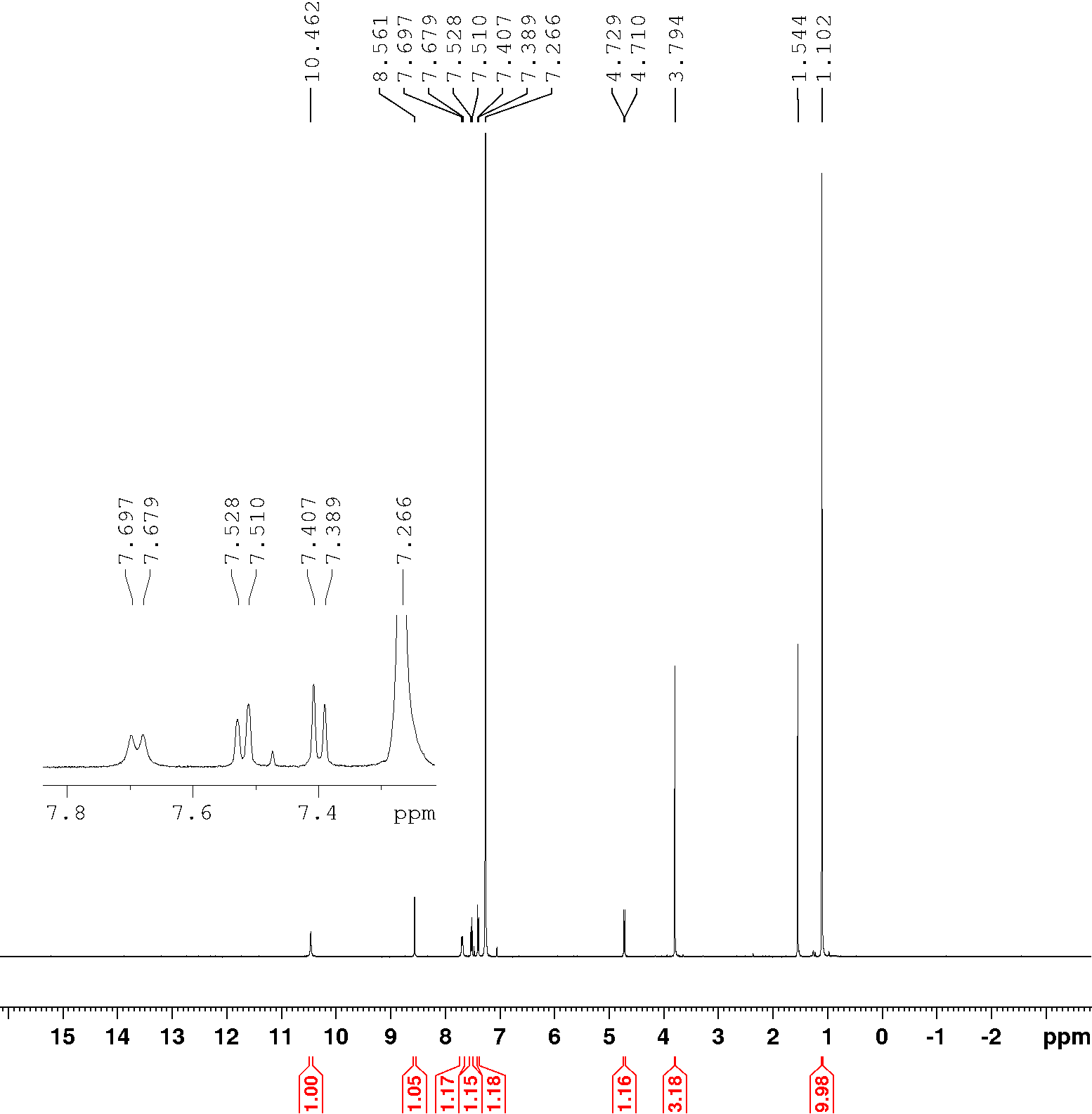


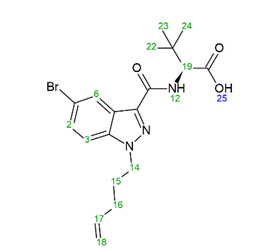
(*2S*)-2-[(5-bromo-1-pent-4-enyl-indazole-3-carbonyl)amino]-3,3-dimethyl-butanoic acid (**7**)


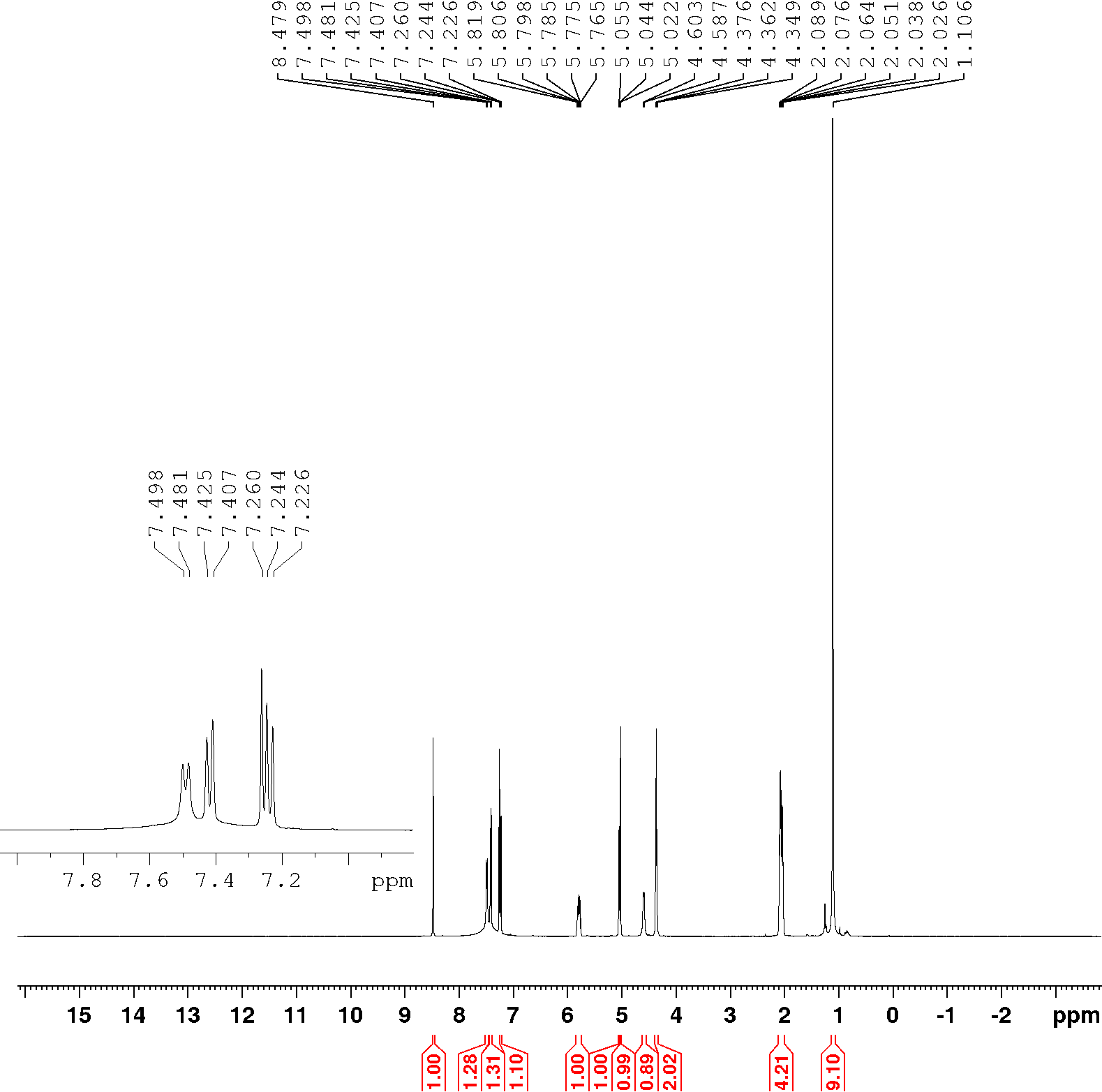


6

12

2

3

17

18

19

14

15, 16

22, 23, 24


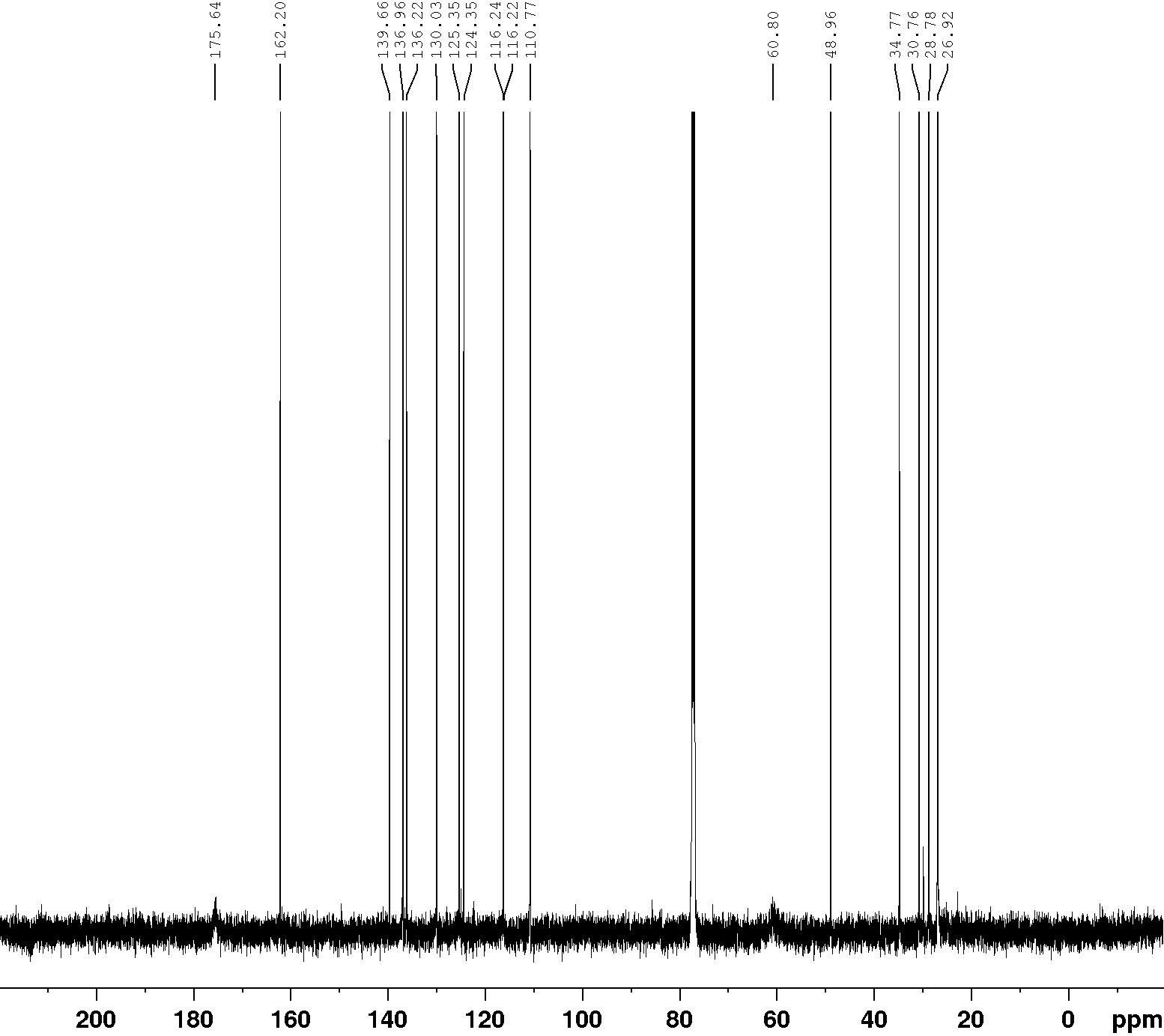


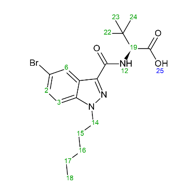
(*2S*)-2-[(5-bromo-1-pentyl-indazole-3-carbonyl)amino]-3,3-dimethyl-butanoic acid (**10**)


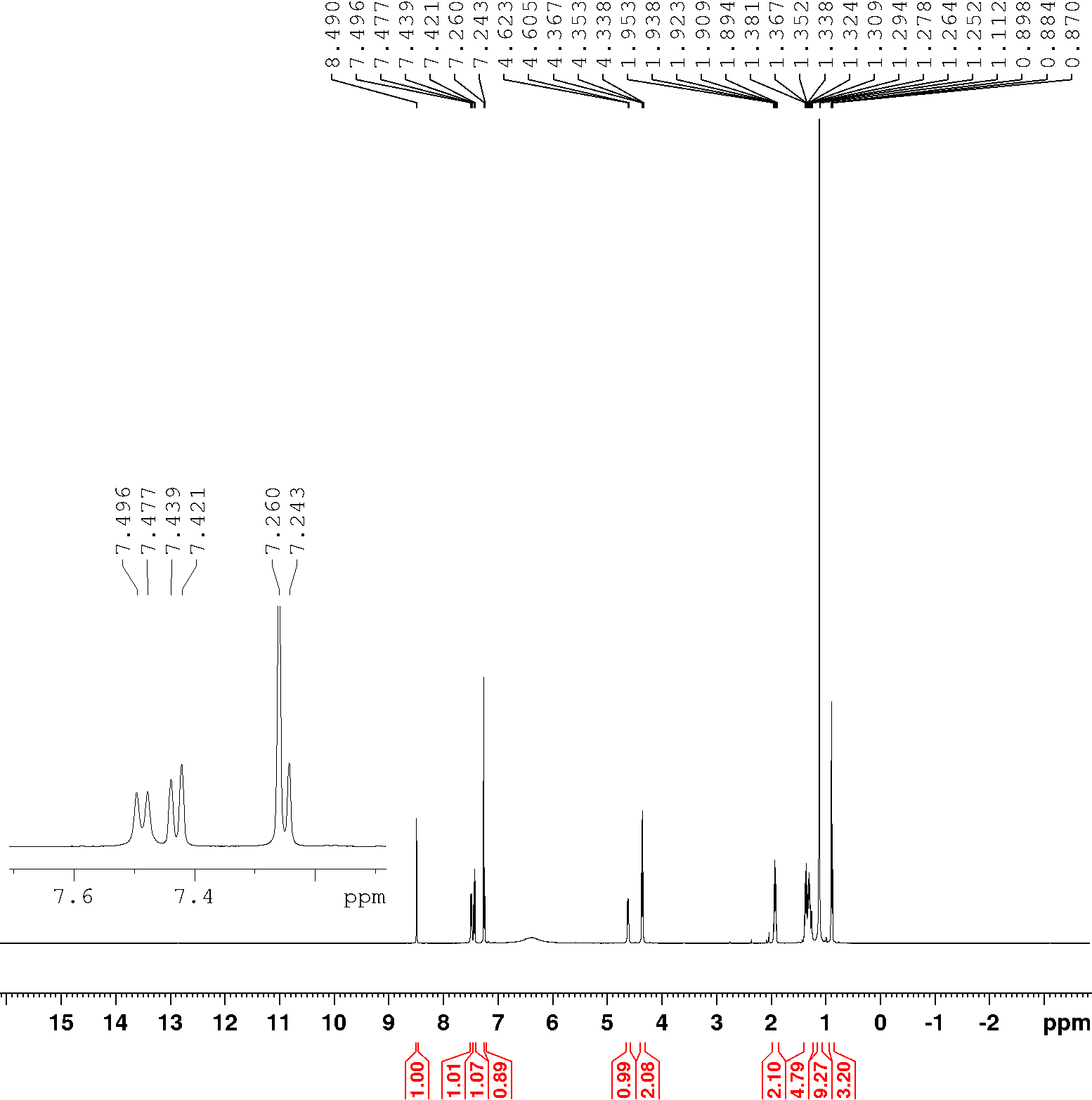


6

12

2

3

16, 17

18

19

14

15

22, 23, 24


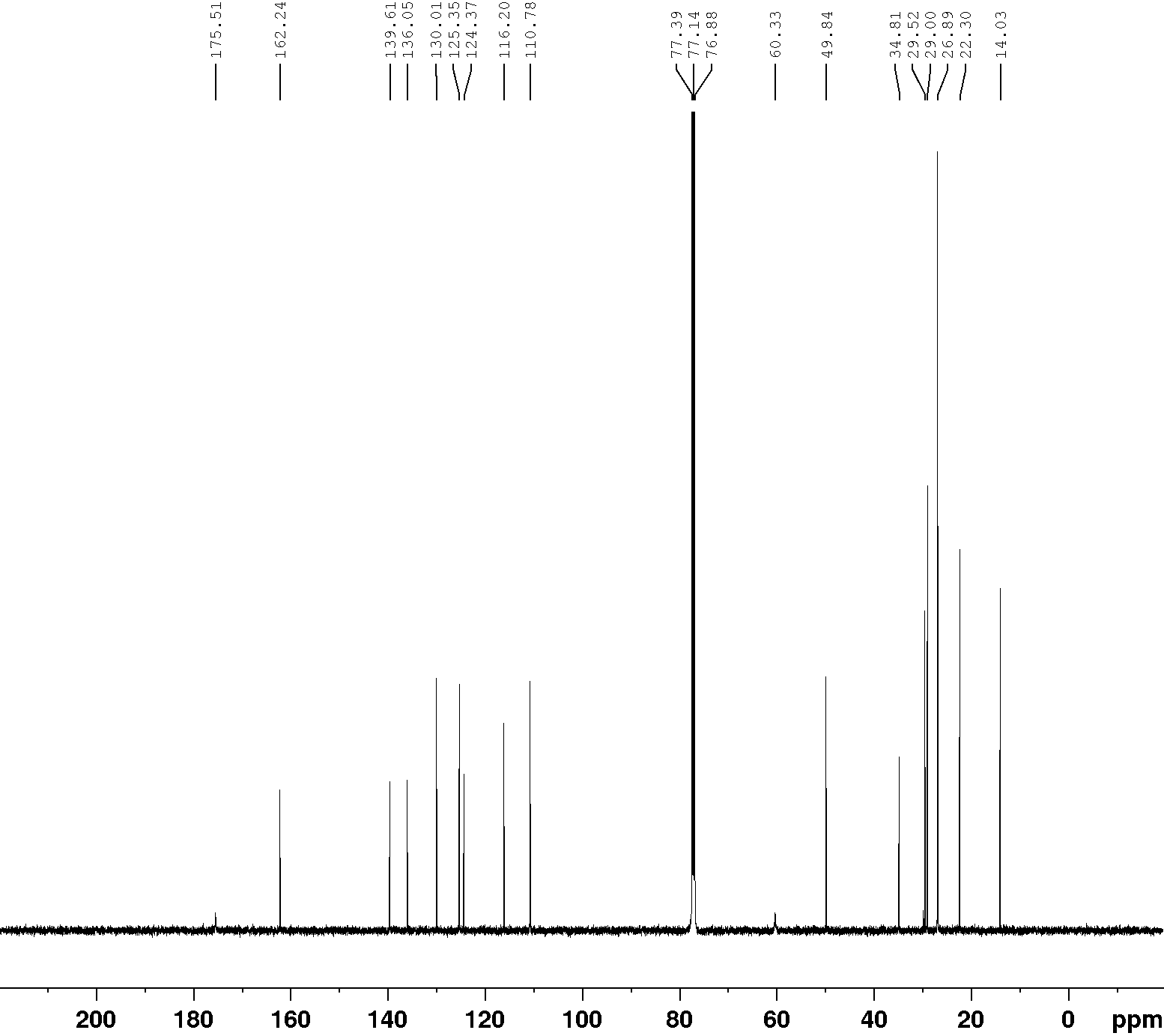


5-bromo-N-[(*1S*)-1-carbamoyl-2,2-dimethyl-propyl]-1H-indazole-3-carboxamide (**12**)


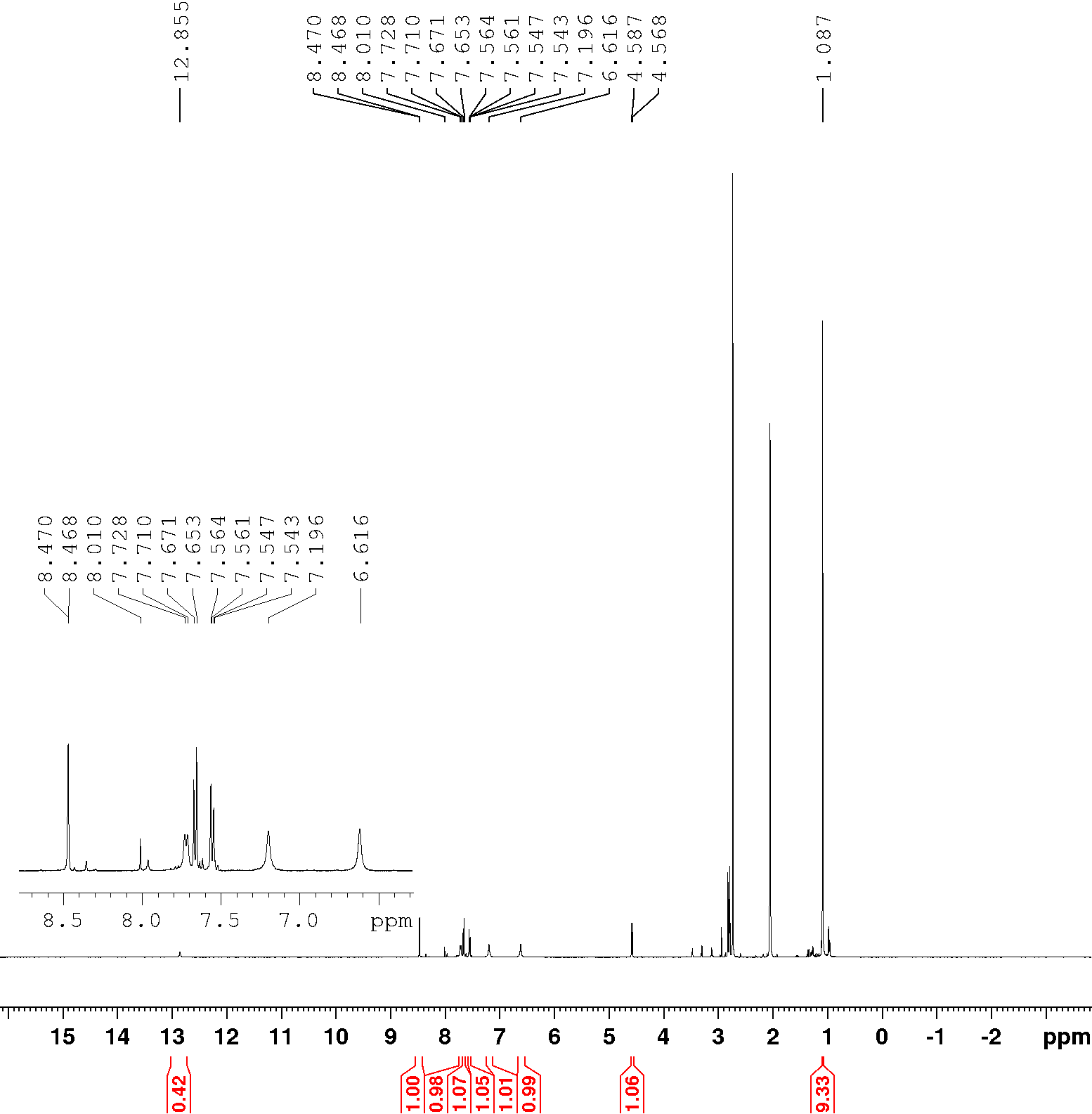


5-bromo-N-[(*1S*)-1-carbamoyl-2,2-dimethyl-propyl]-1-[3-(oxiran-2-yl)propyl]indazole-3-carboxamide (**13**)


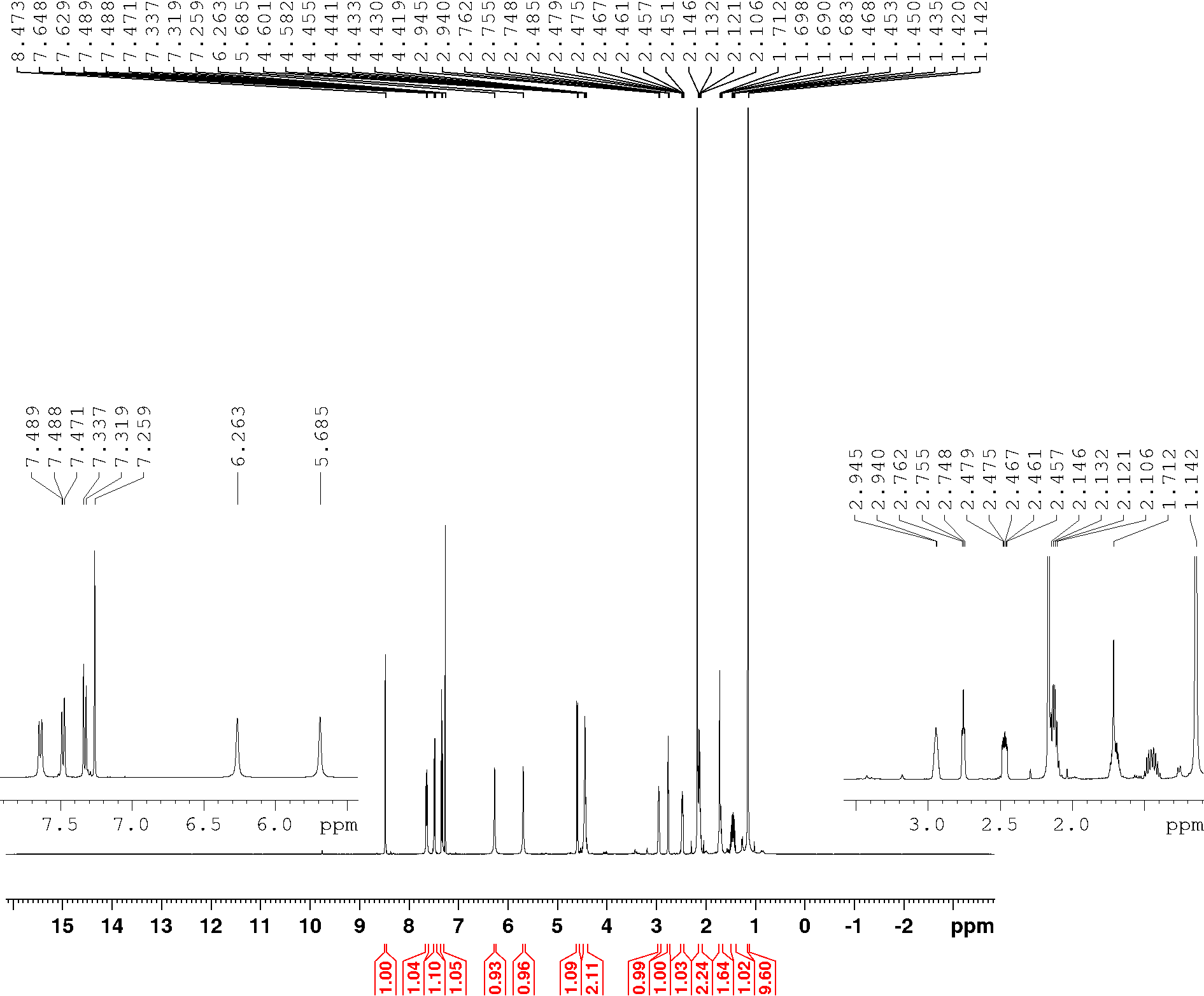


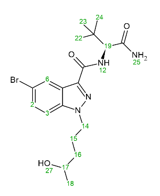
5-bromo-N-[(*1S*)-1-carbamoyl-2,2-dimethyl-propyl]-1-(4-hydroxypentyl)indazole-3-carboxamide (**14**)


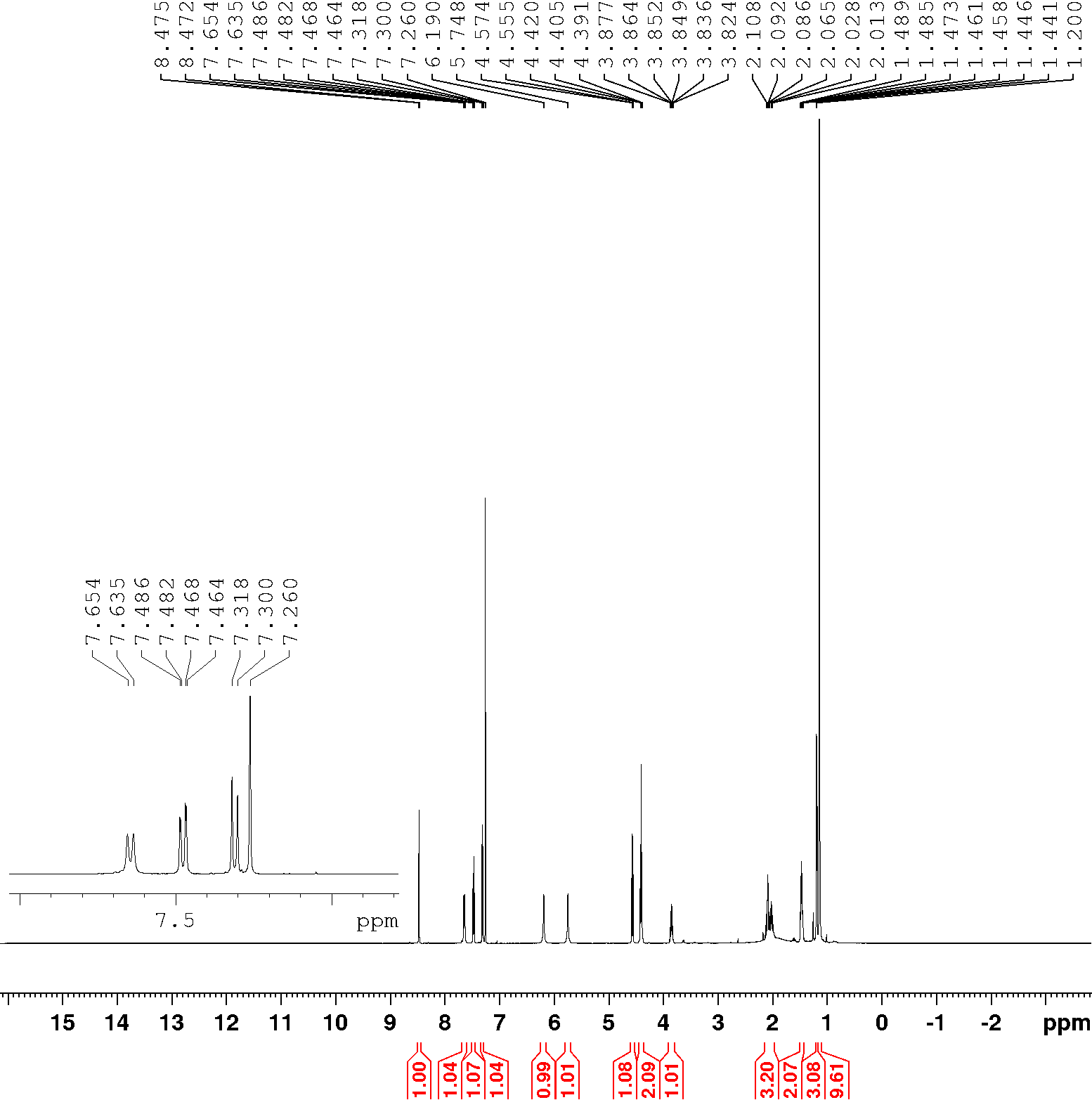


6

12

2

3

16

18

19

14

15,27

22, 23, 24

25

25

17


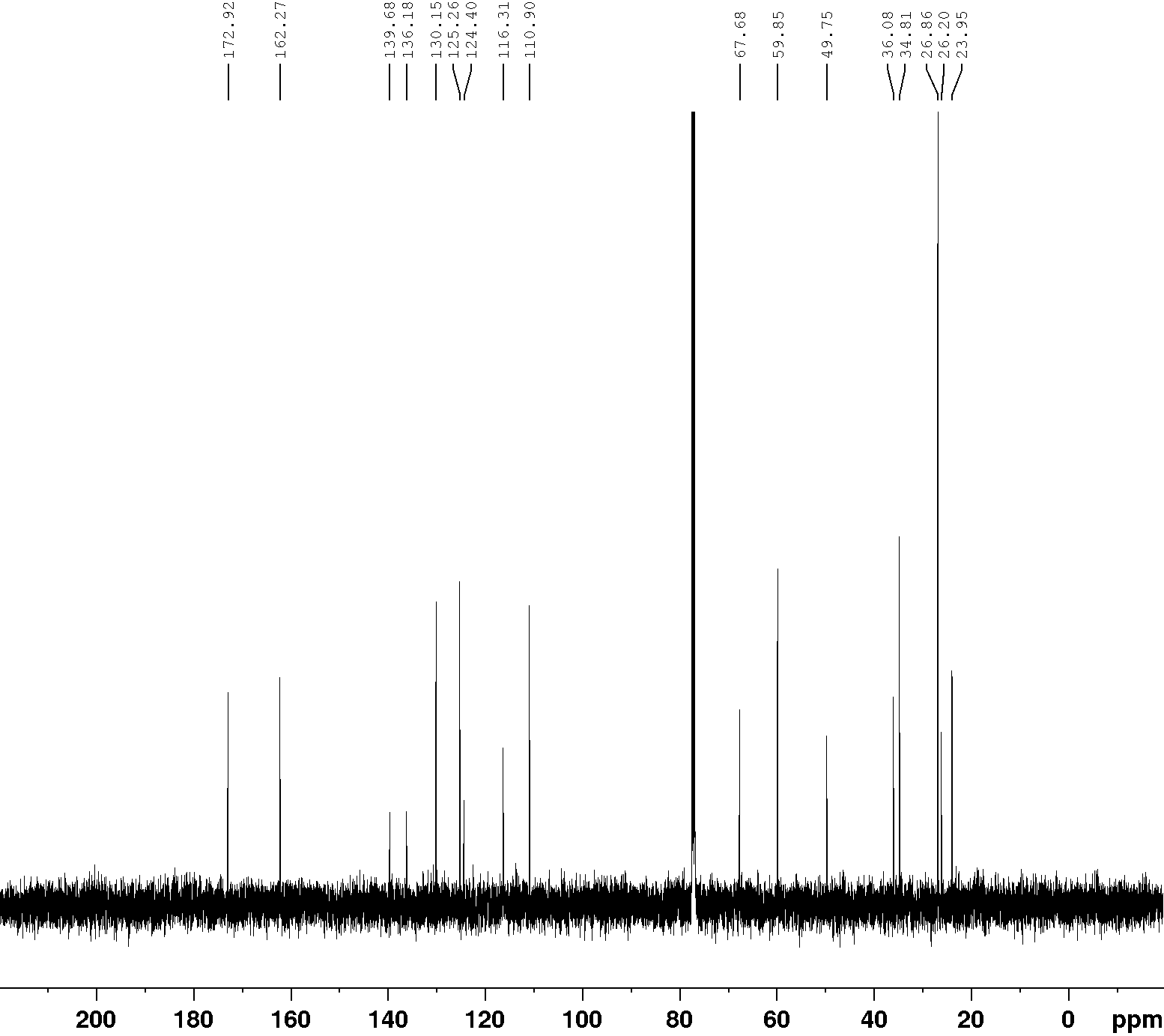


5-bromo-N-[(*1S*)-1-carbamoyl-2,2-dimethyl-propyl]-1-(4,5-dihydroxypentyl)indazole-3-carboxamide (**15**)


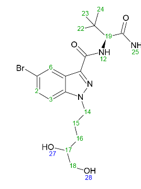

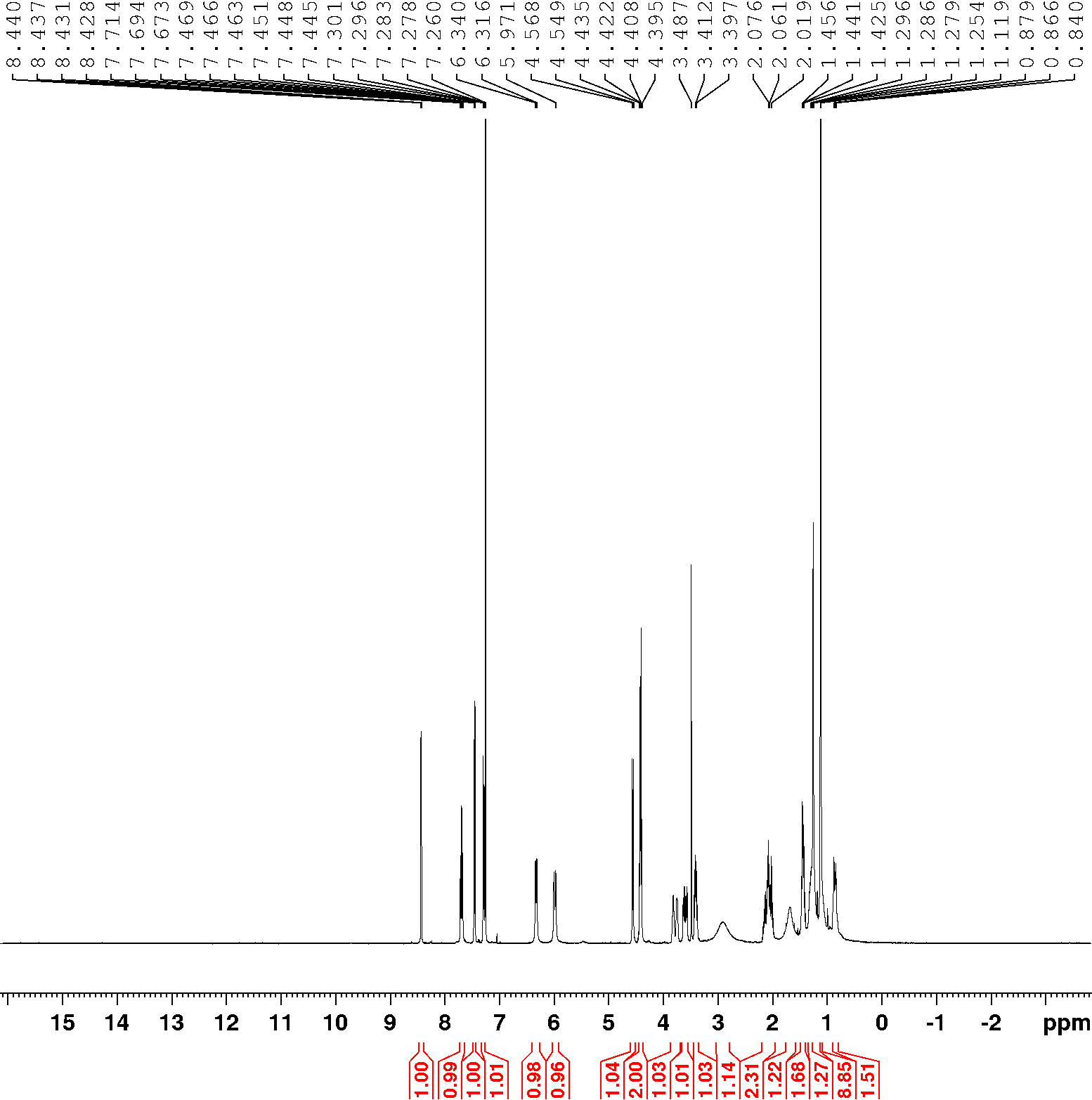


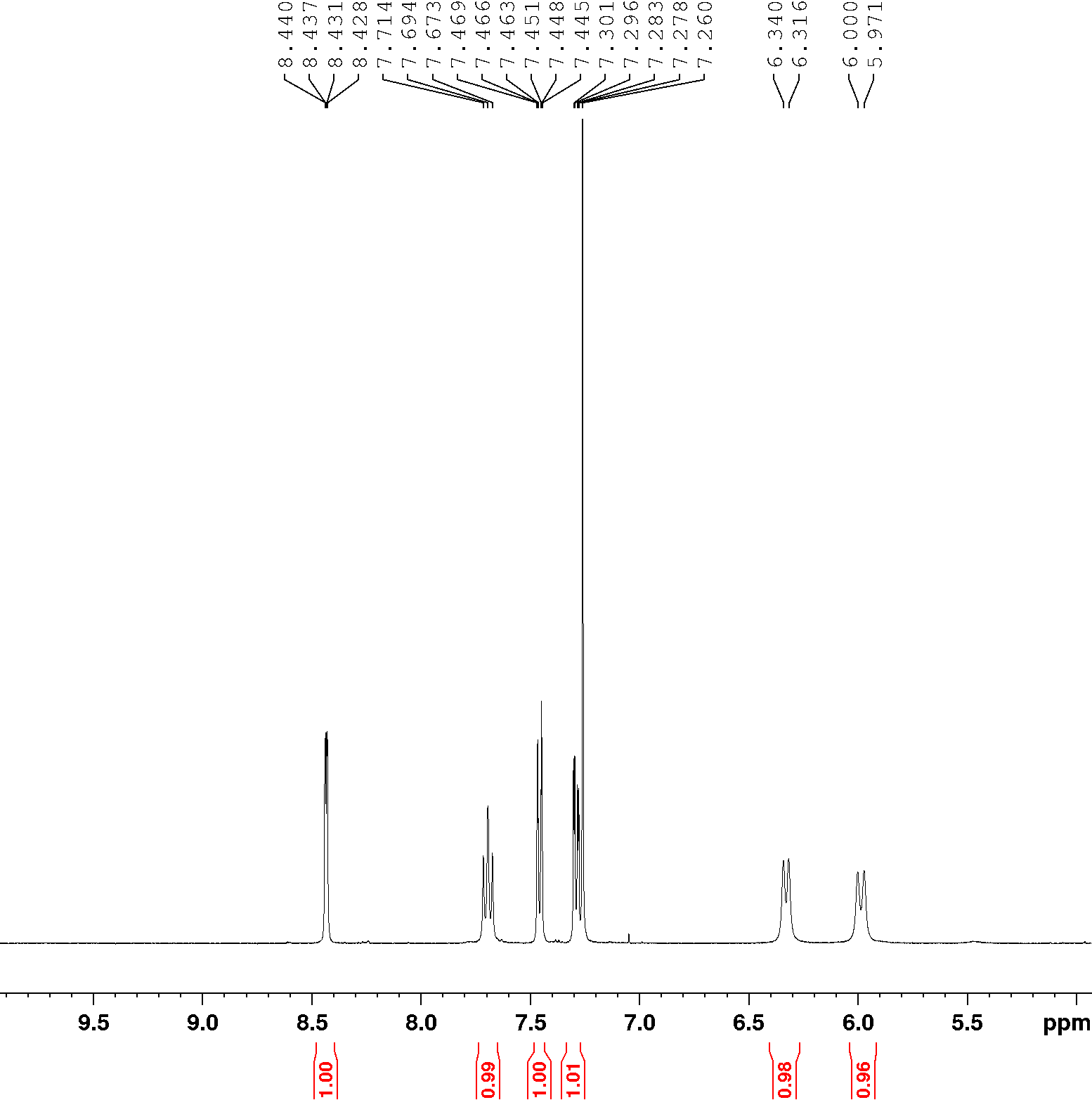


6

12

2

3

25

25


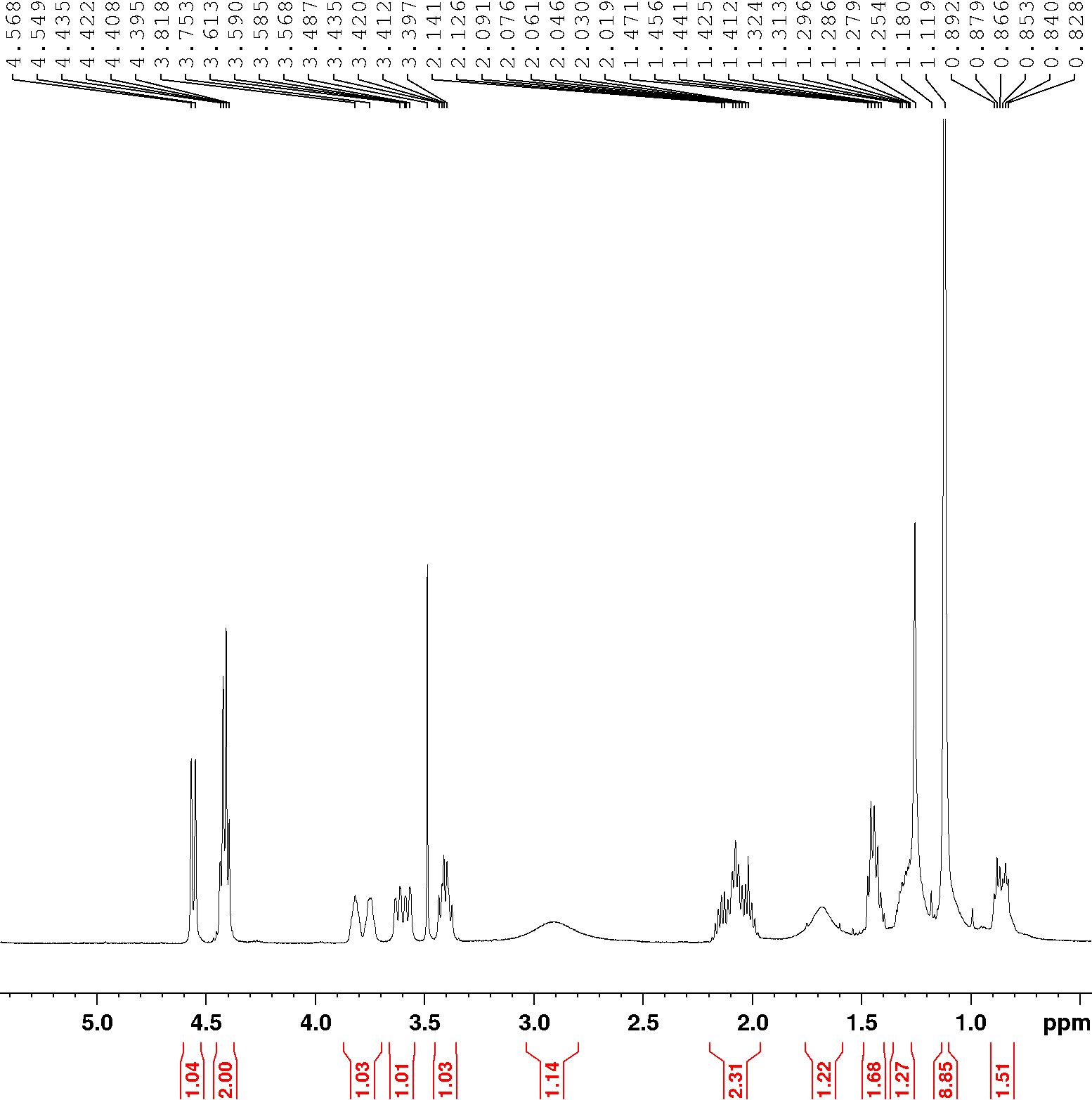


16

18

19

14

27

22, 23, 24

17

18

28

15

16


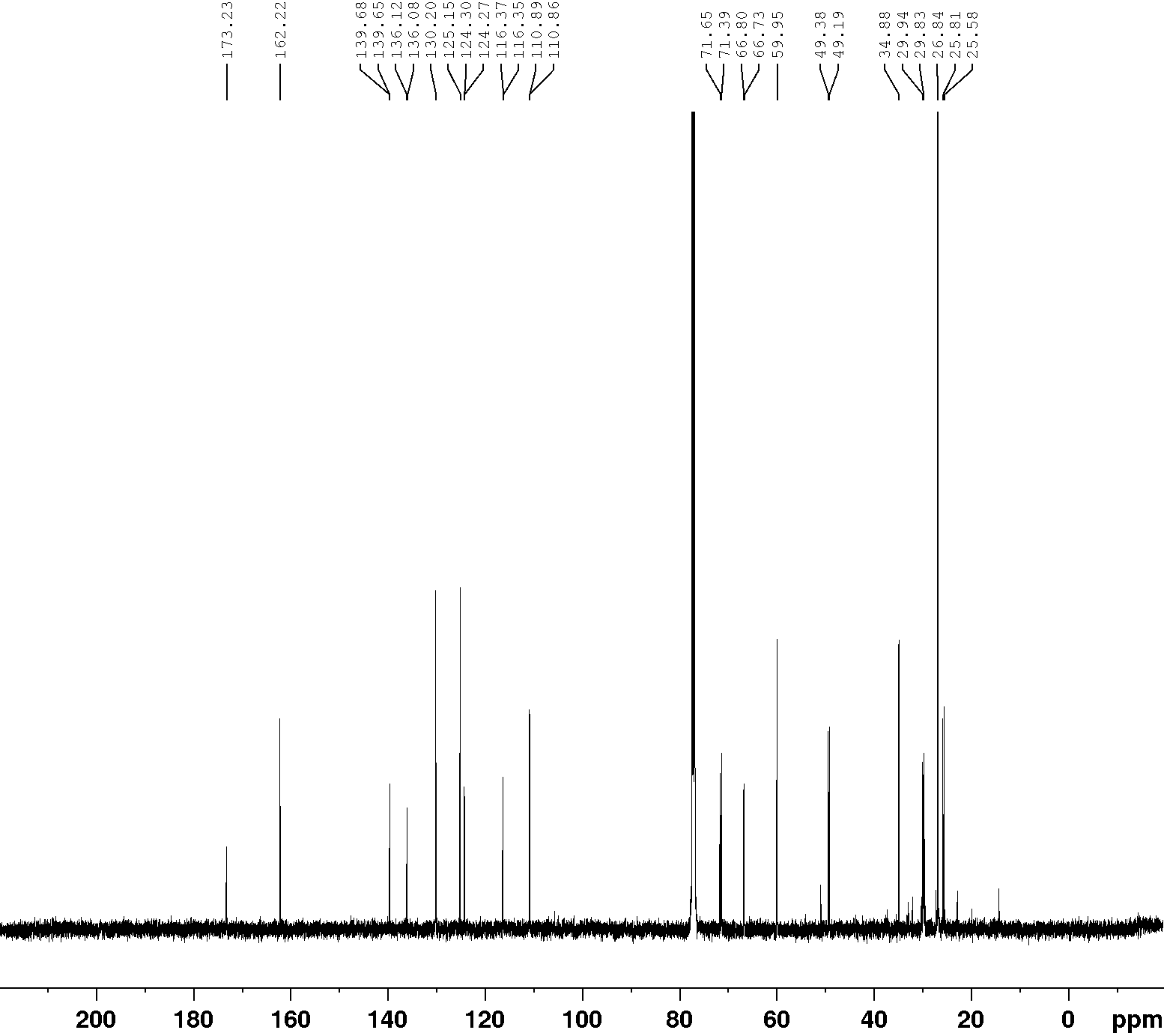


5-bromo-N-[(*1S*)-1-carbamoyl-2,2-dimethyl-propyl]-1-(5-hydroxypentyl)indazole-3-carboxamide (**18**)


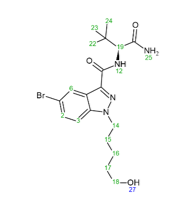


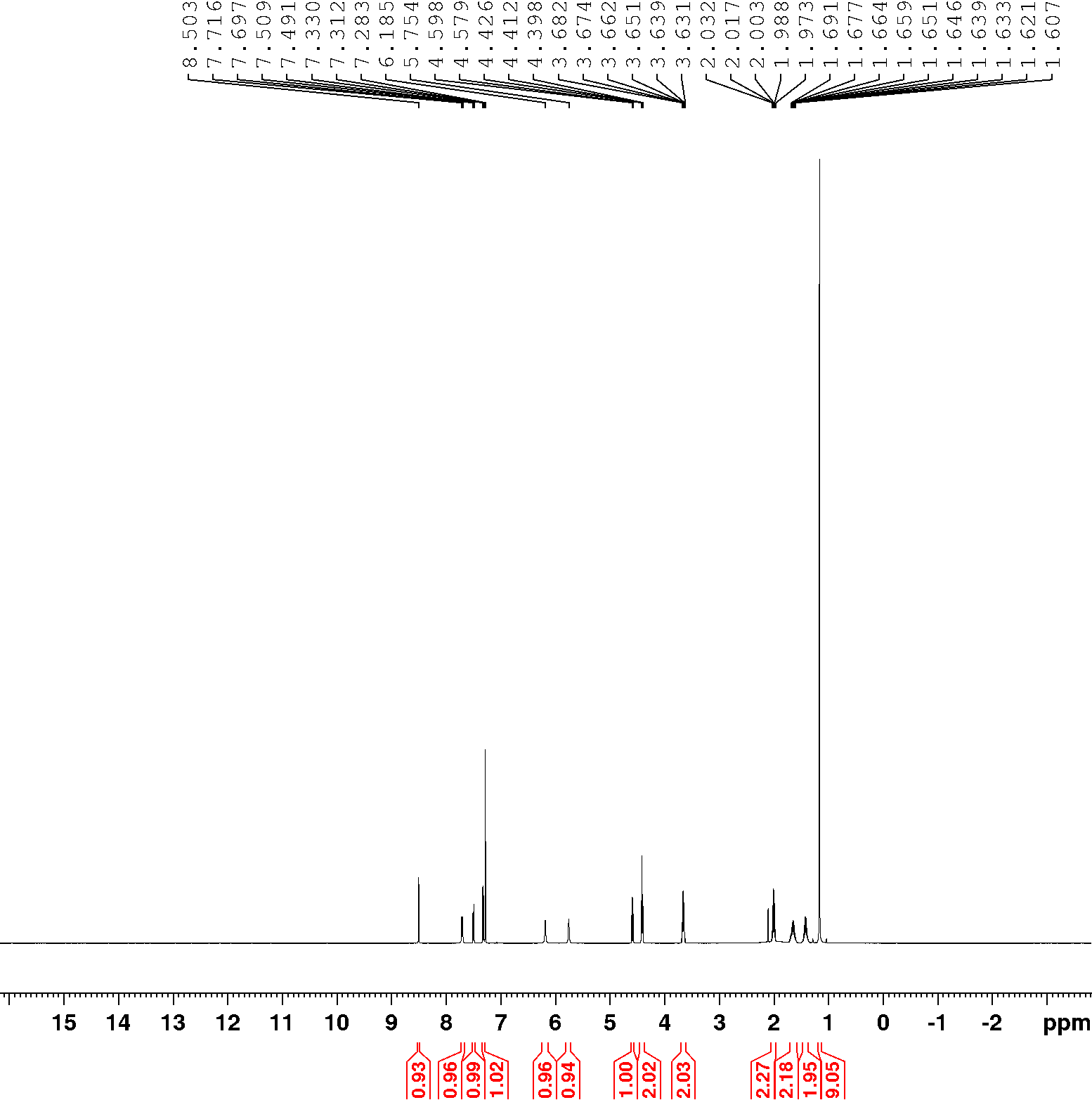


6


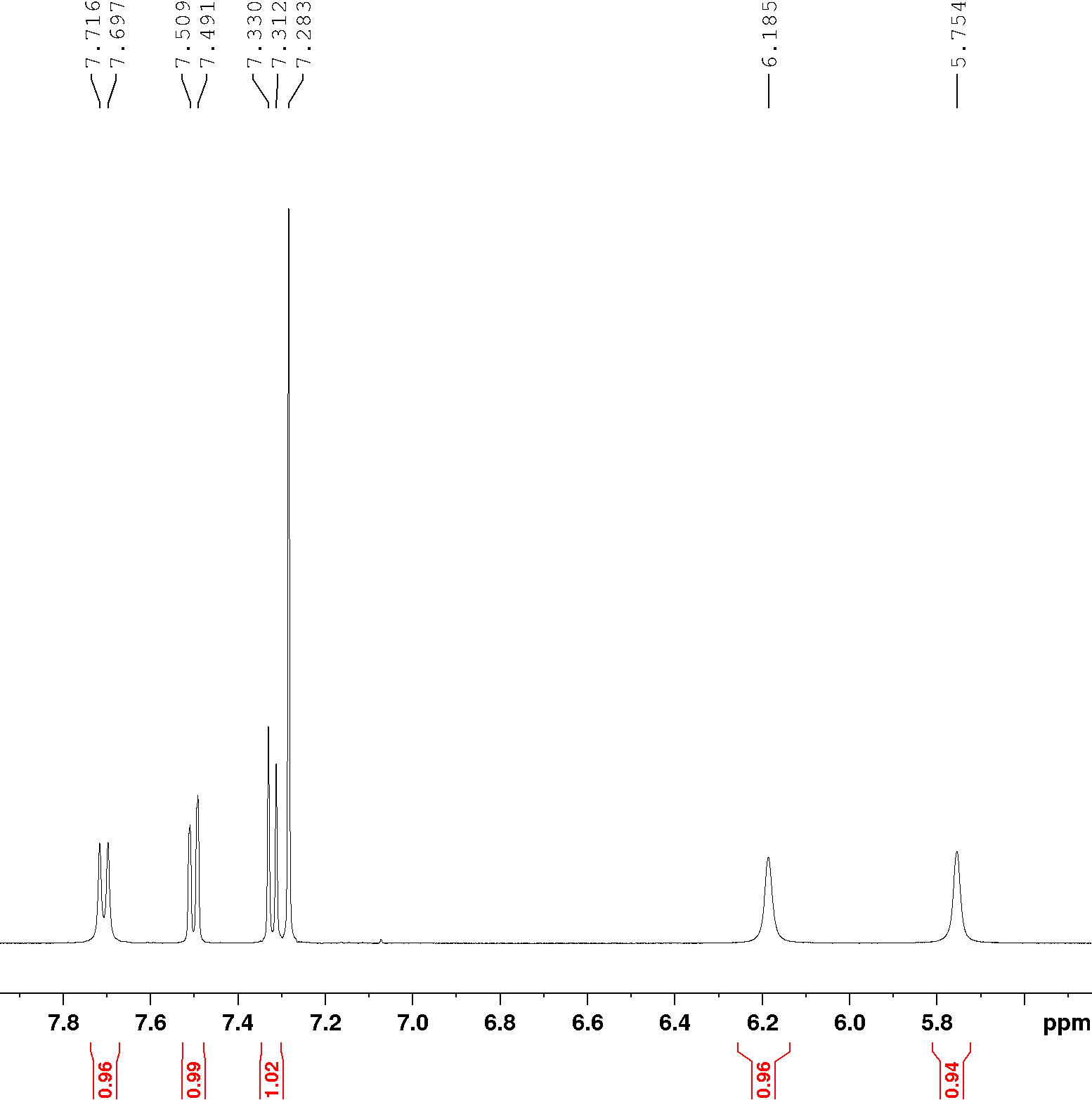


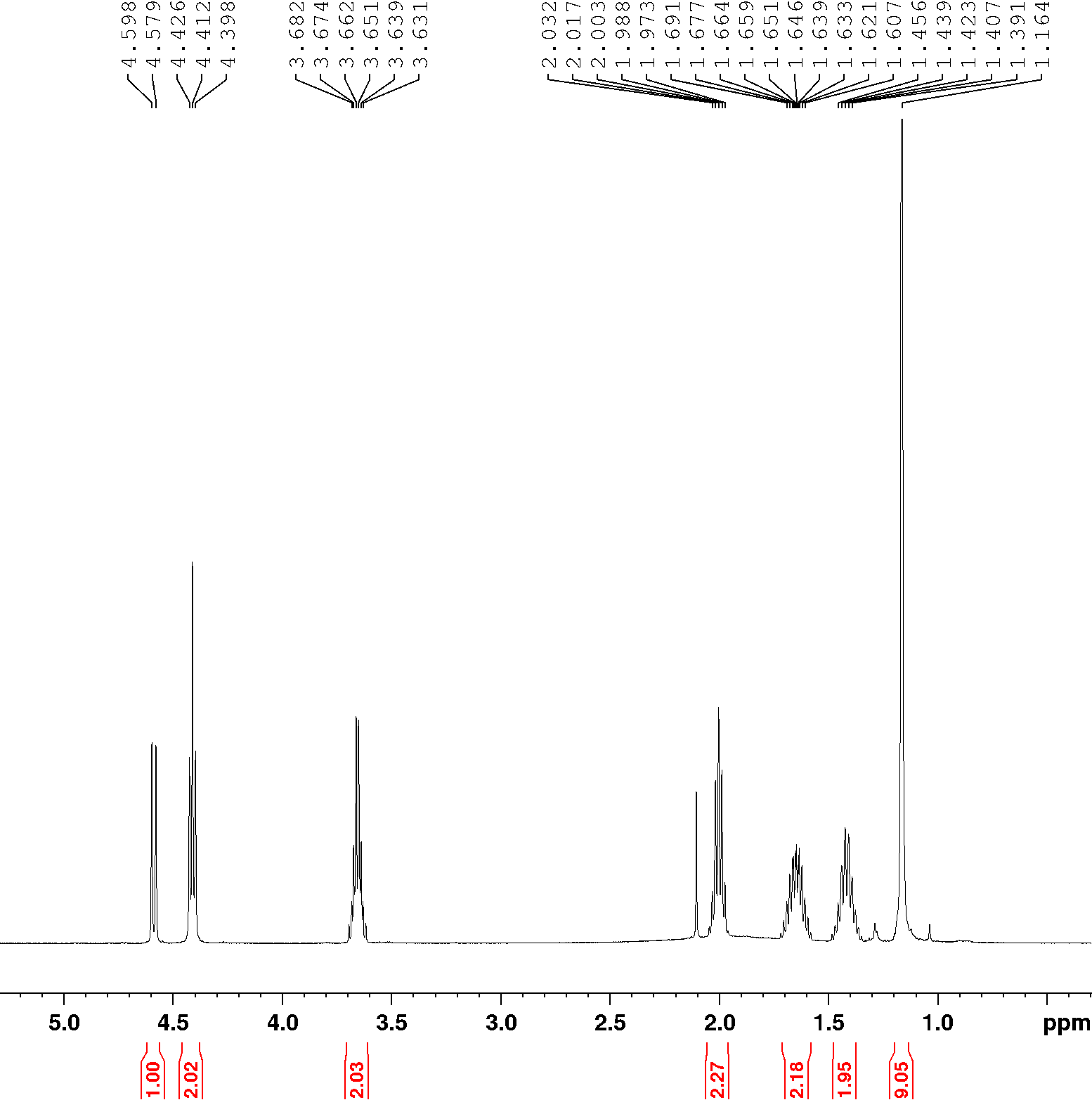


16

18

19

14

22, 23, 24

15

17


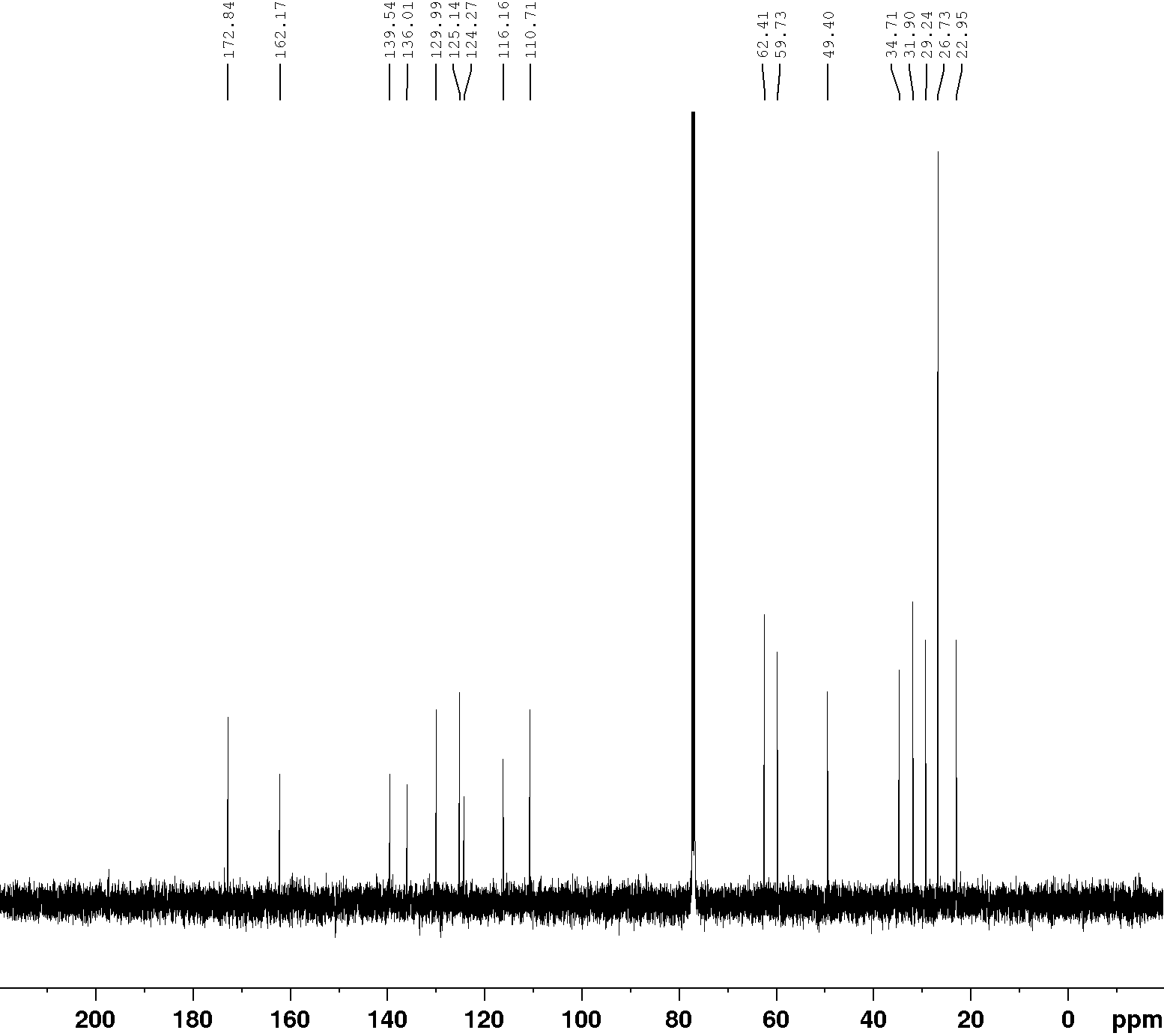


5-bromo-N-[(*1S*)-1-carbamoyl-2,2-dimethyl-propyl]-1-[3-(oxiran-2-yl)propyl]indazole-3-carboxamide (**19**)


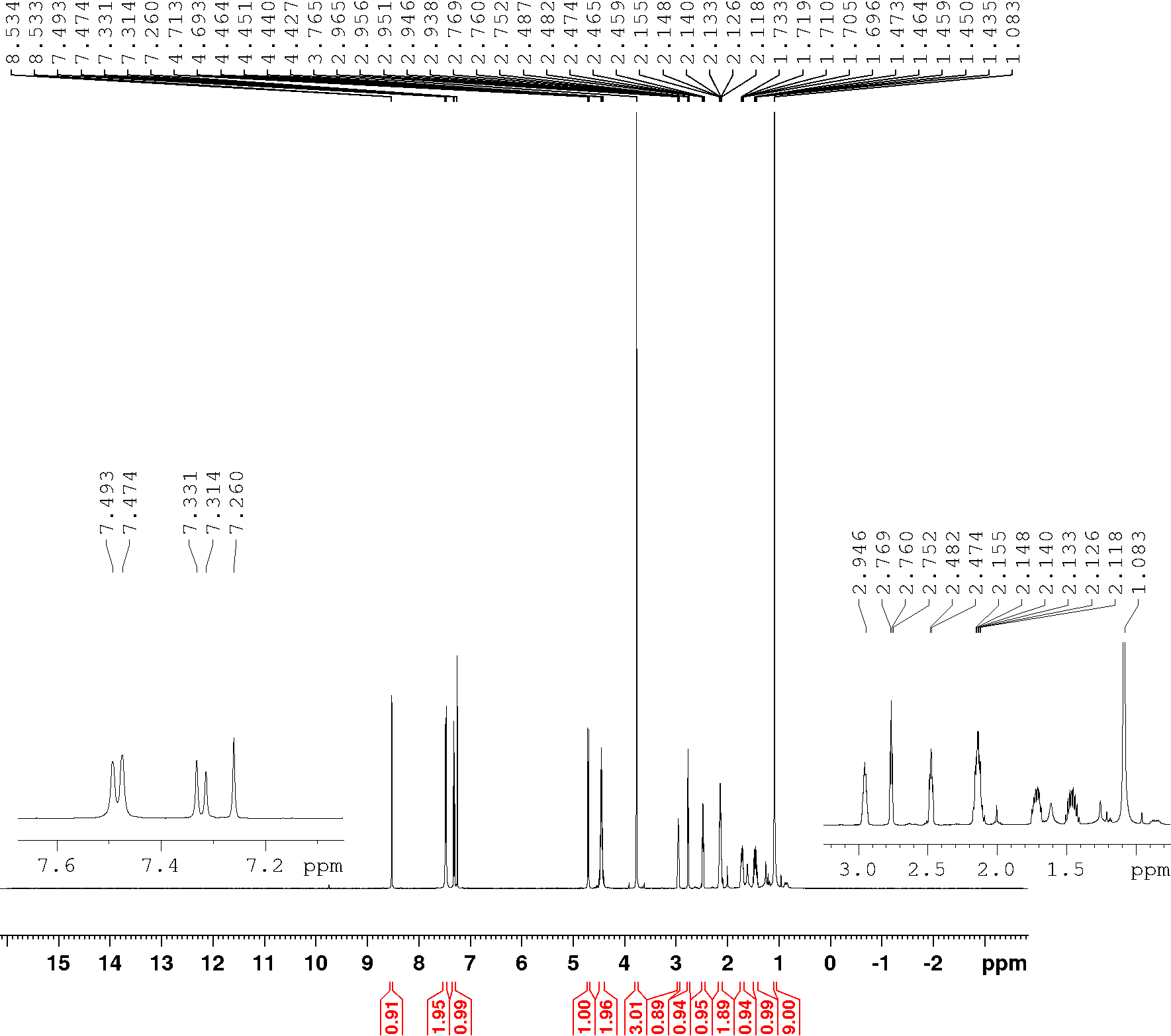


(*2S*)-2-[[5-bromo-1-(4,5-dihydroxypentyl)indazole-3-carbonyl]amino]-3,3-dimethyl-butanoic acid (**21**)


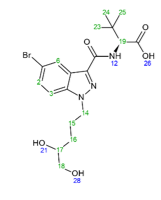

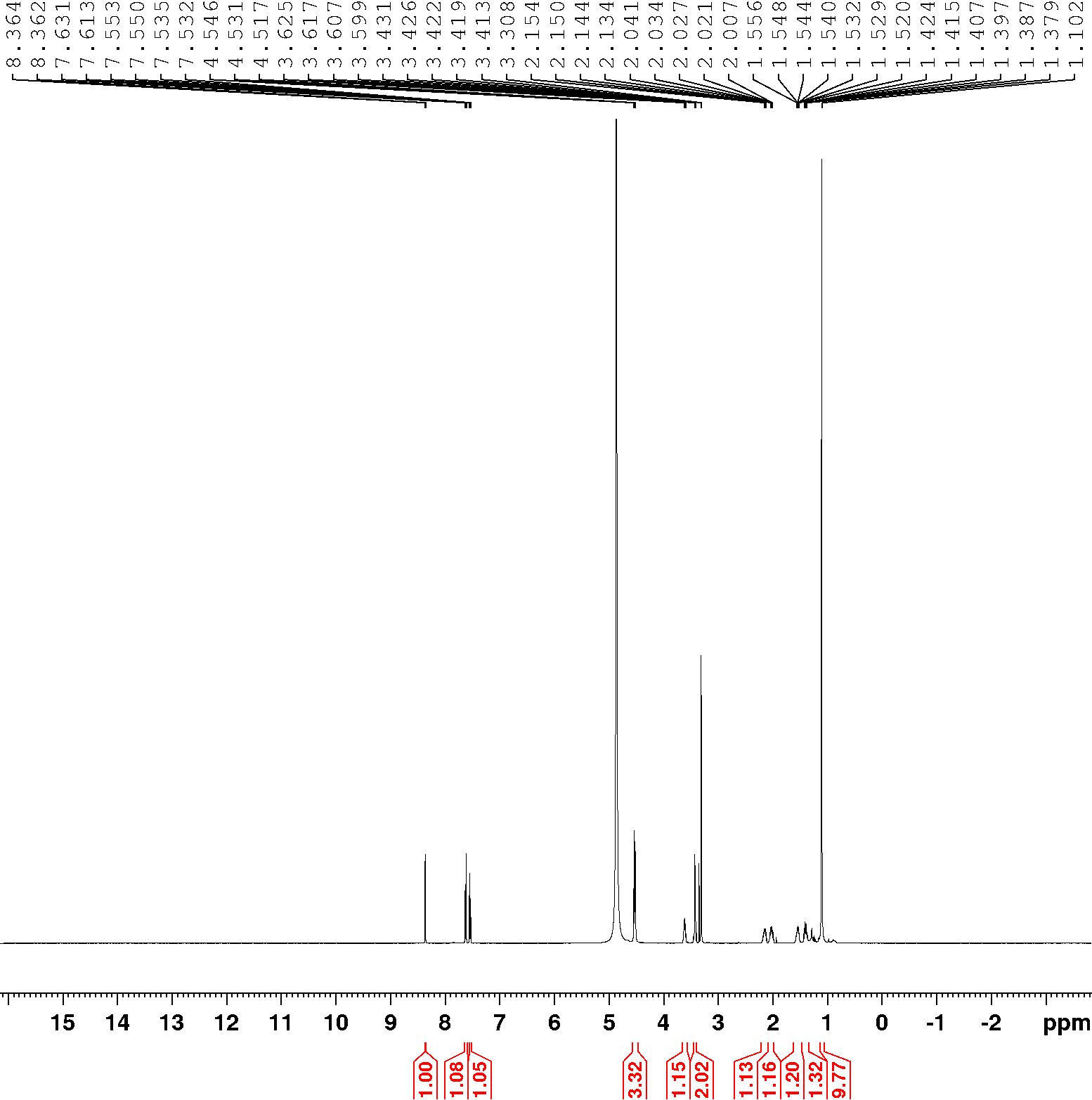


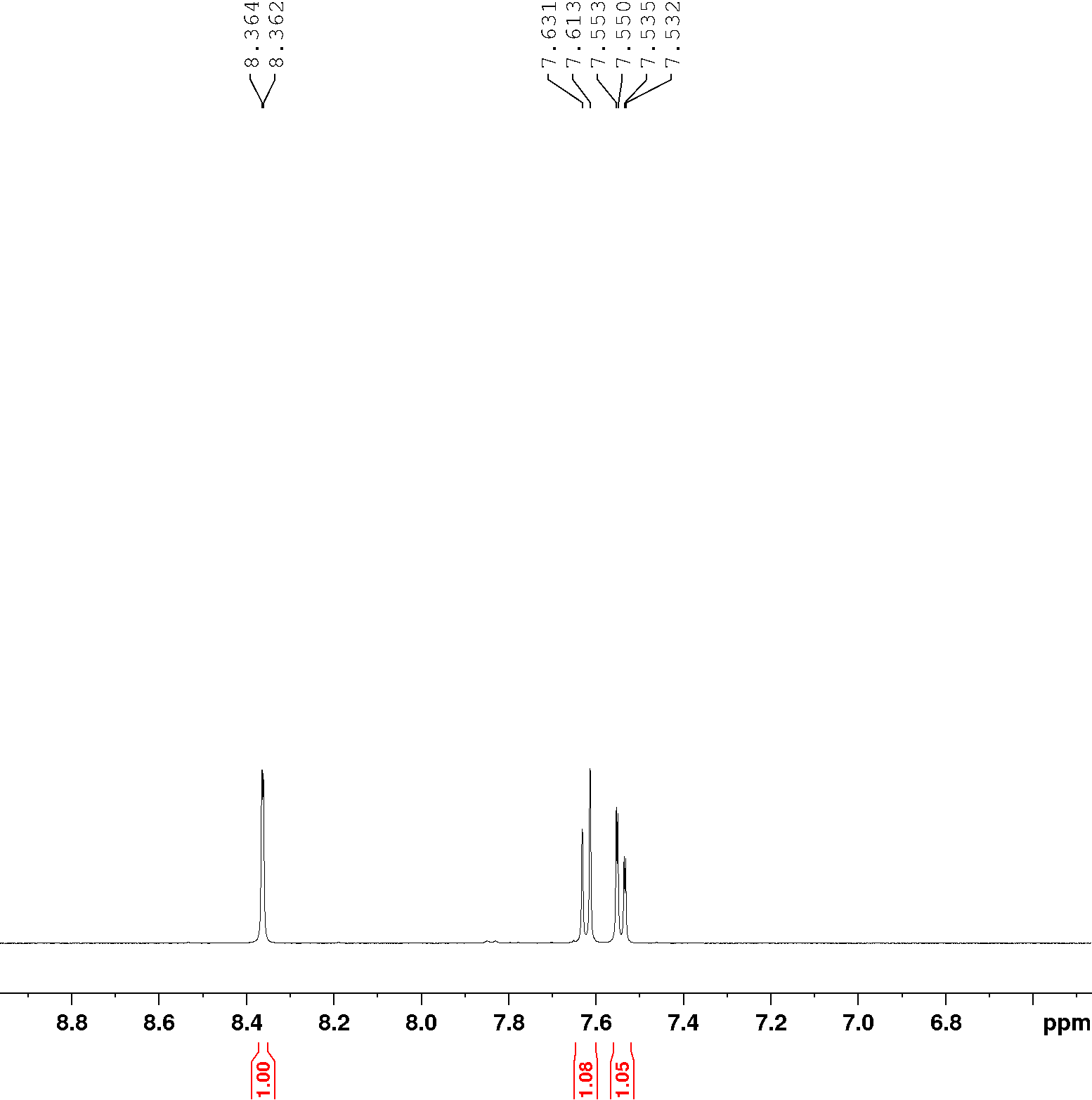


6

2

3


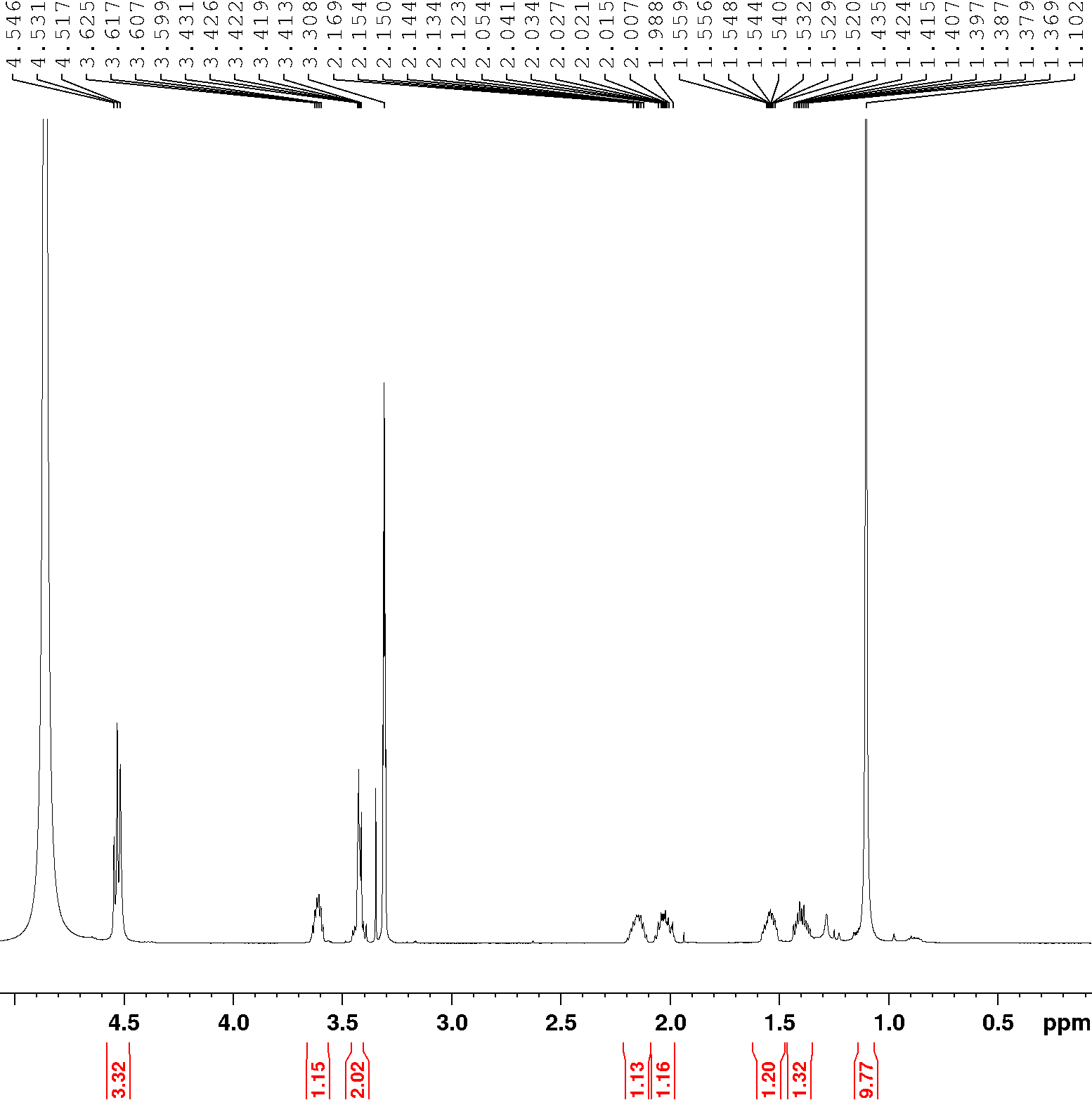


14, 19

17

18

15

16

23, 24, 25

**
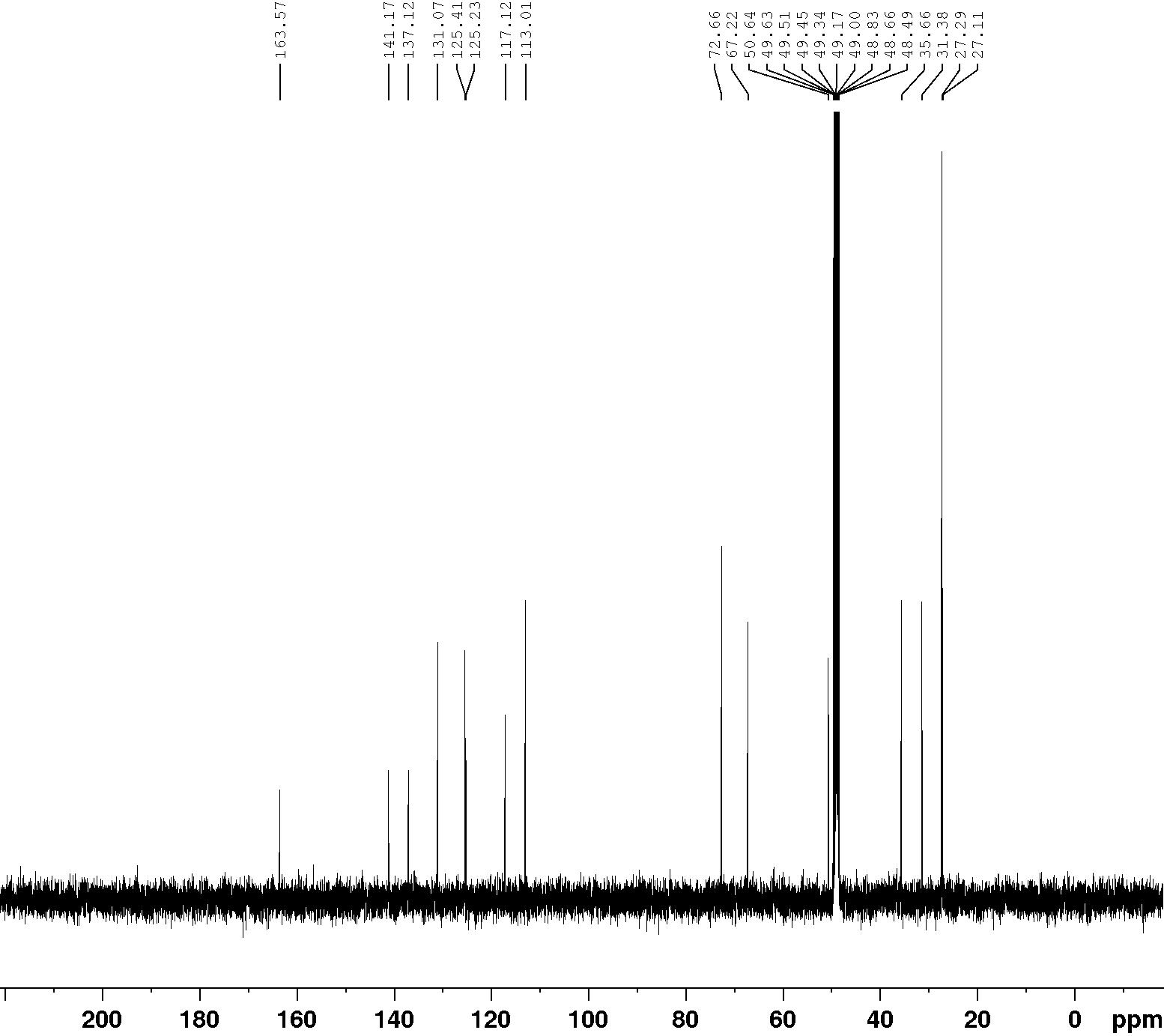
**

5-bromo-N-[(*1S*)-1-carbamoyl-2,2-dimethyl-propyl]-1-(4-oxopentyl)indazole-3-carboxamide (**22**)


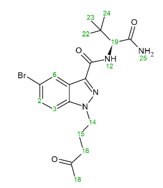


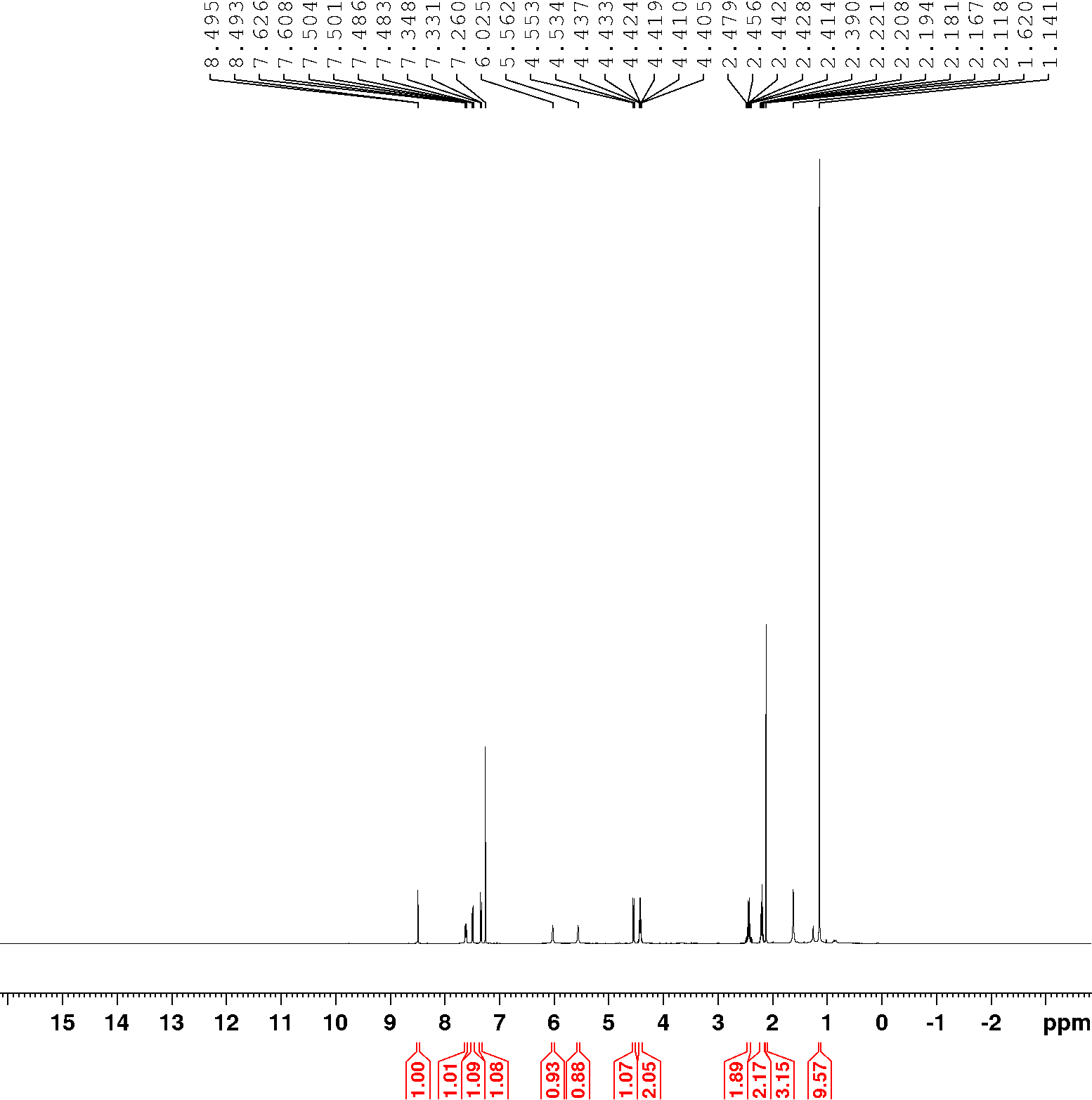


6

25

25


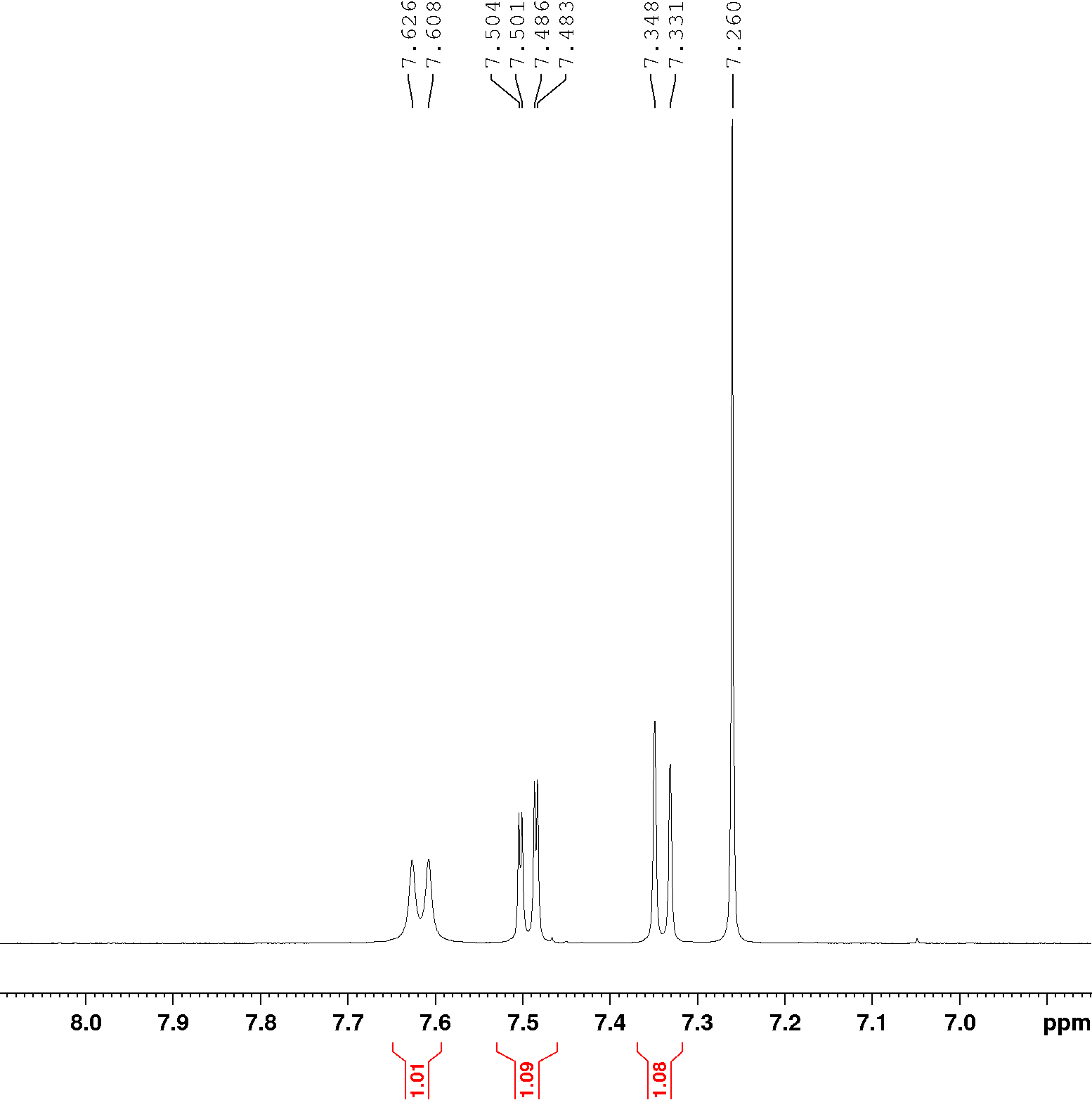


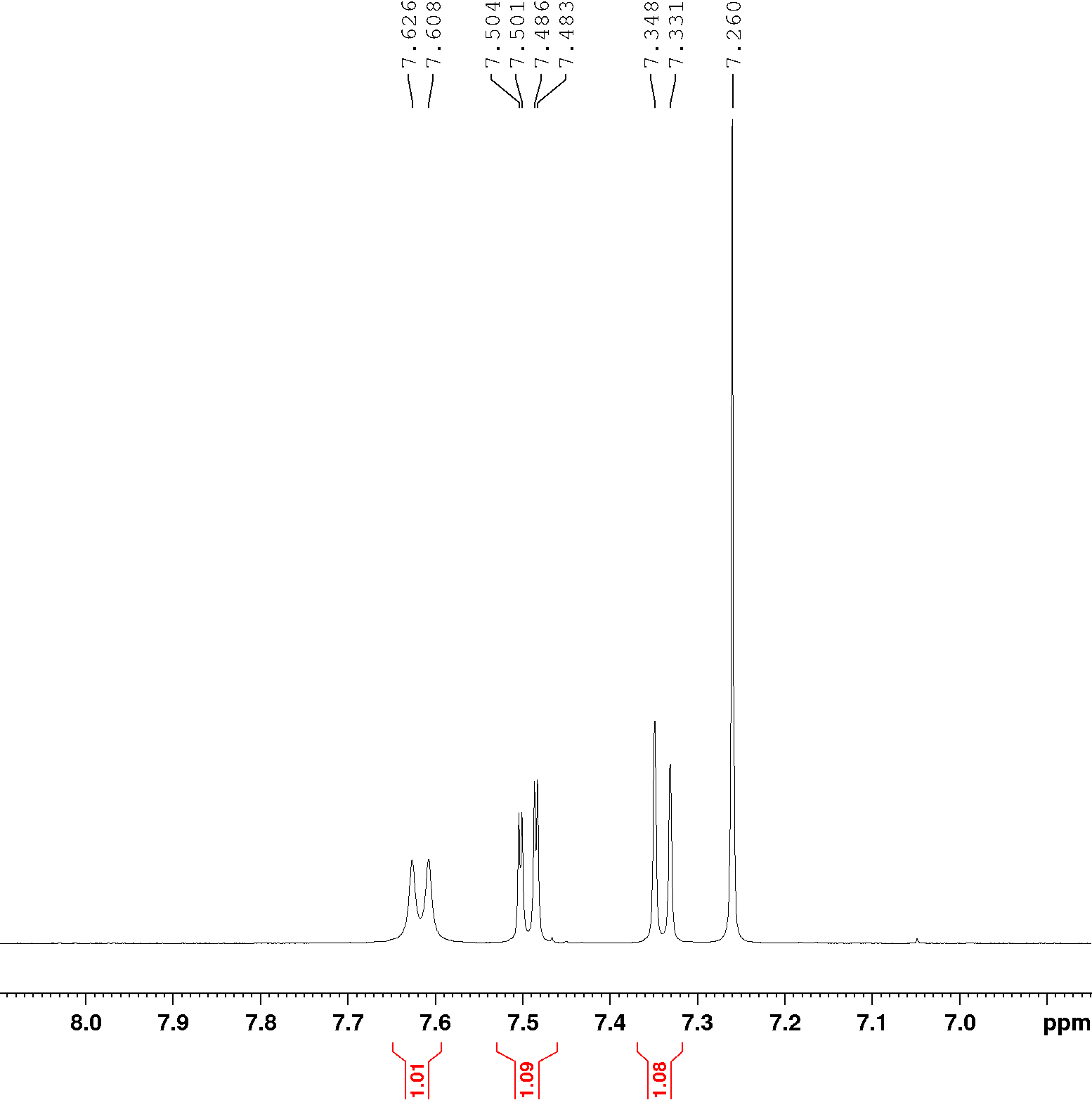


12

2

3


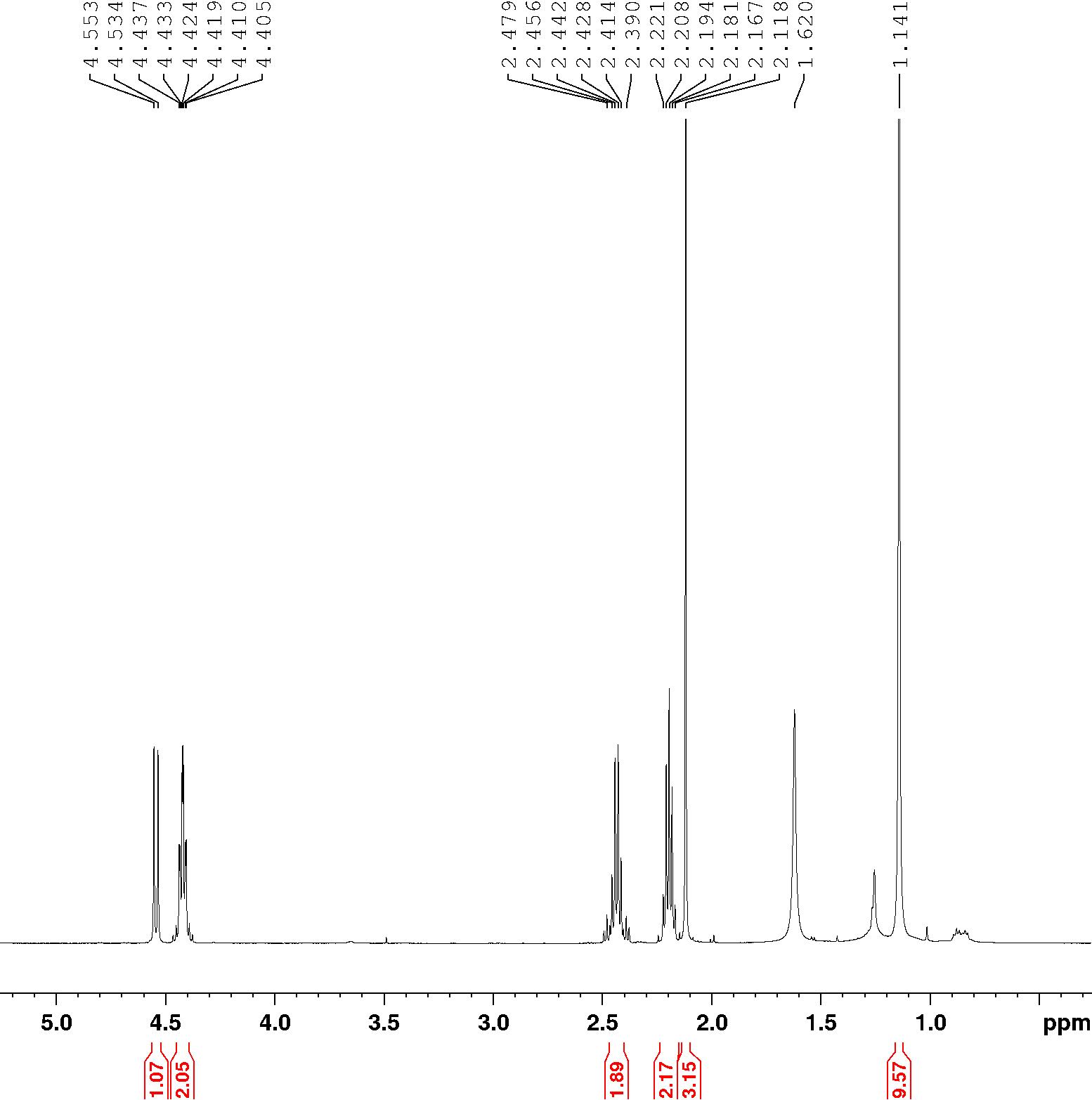


19

22, 23, 24

15

14

16

18


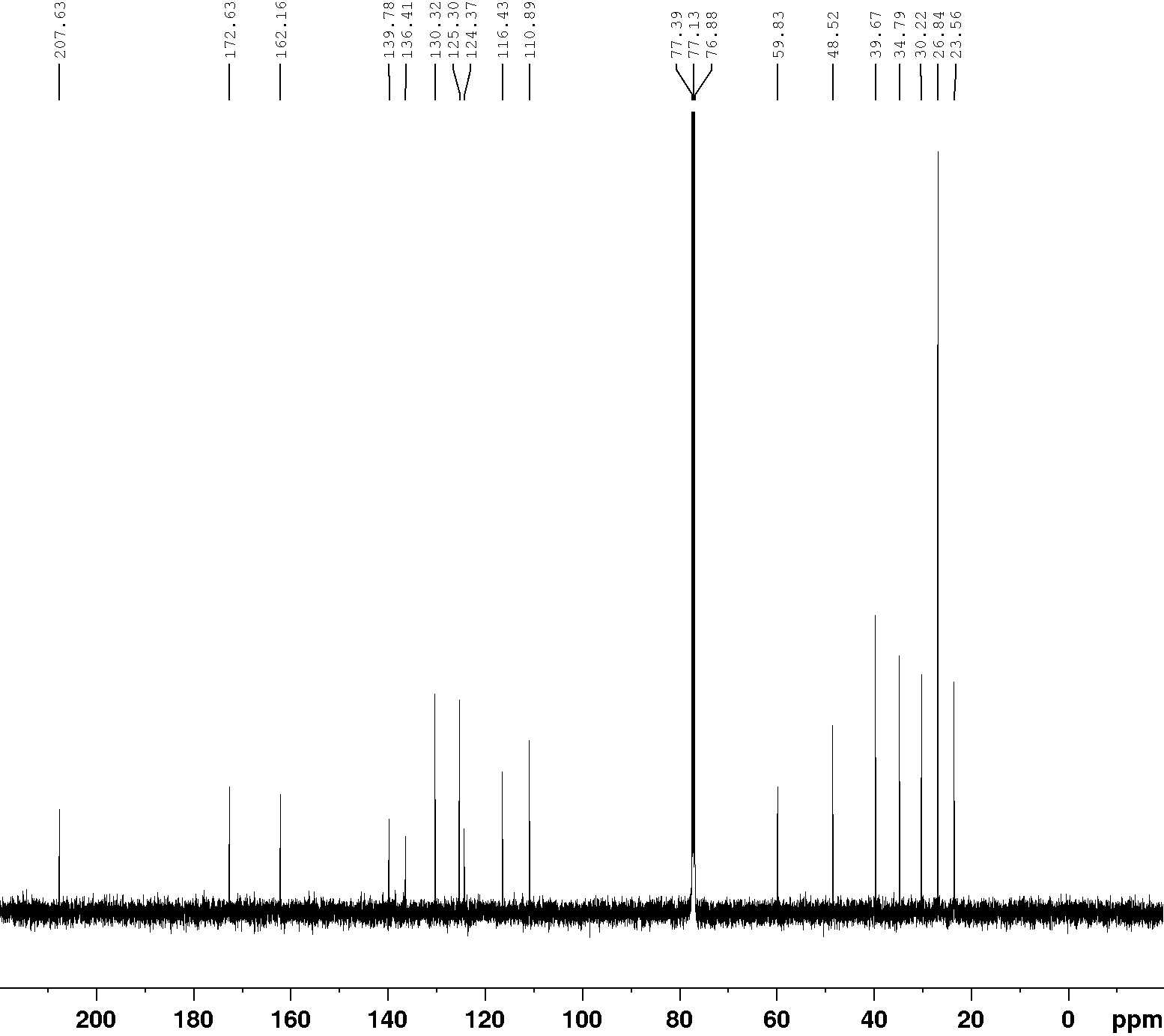


5-bromo-1-(3-hydroxypropyl)indazole-3-carboxylic acid (**33**)


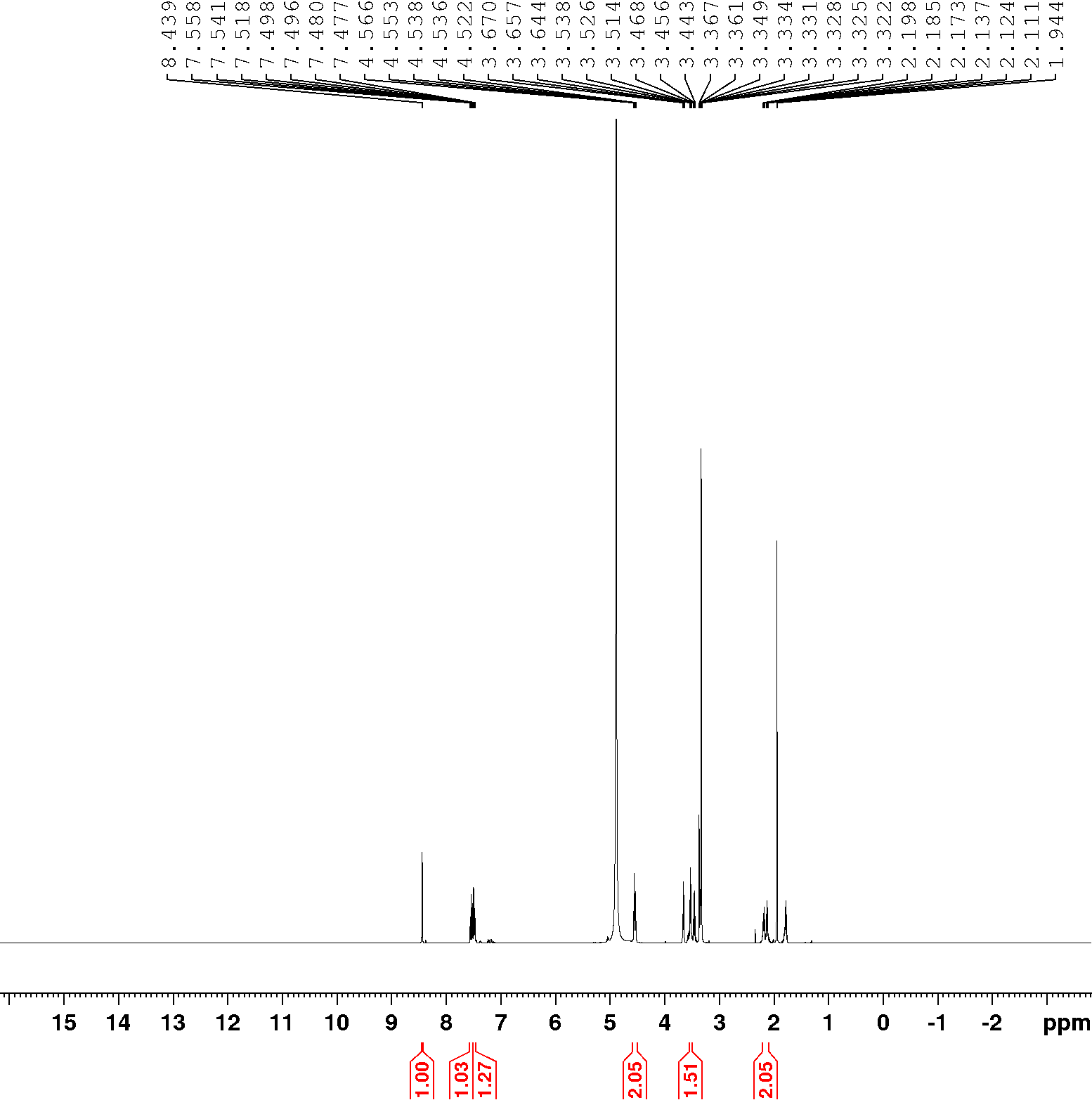


5-bromo-N-[(*1S*)-1-carbamoyl-2,2-dimethyl-propyl]-1-(3-hydroxypent-4-enyl)indazole-3-carboxamide (**38**)

**
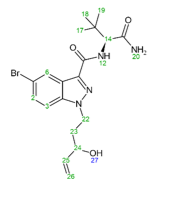
**
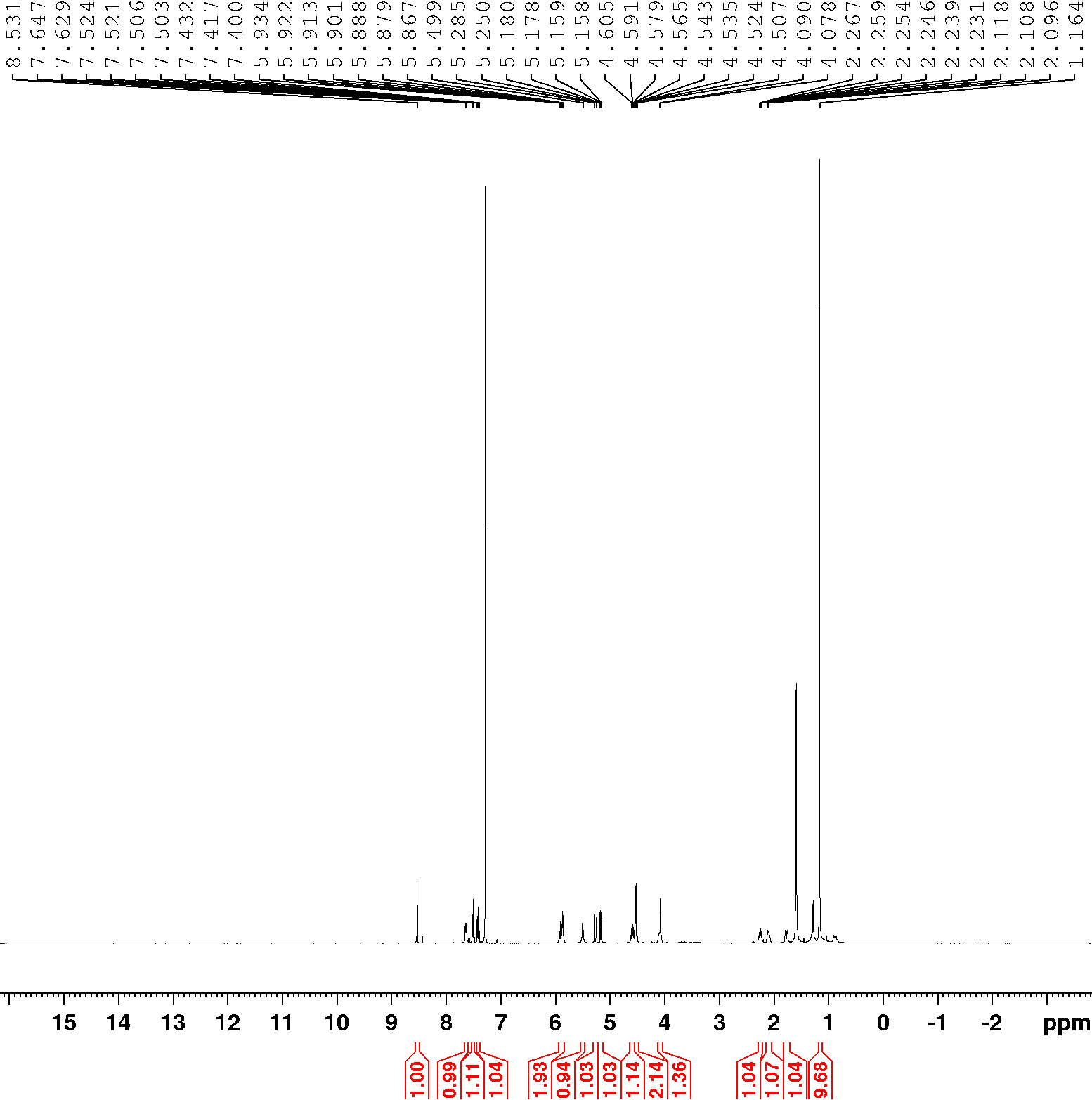


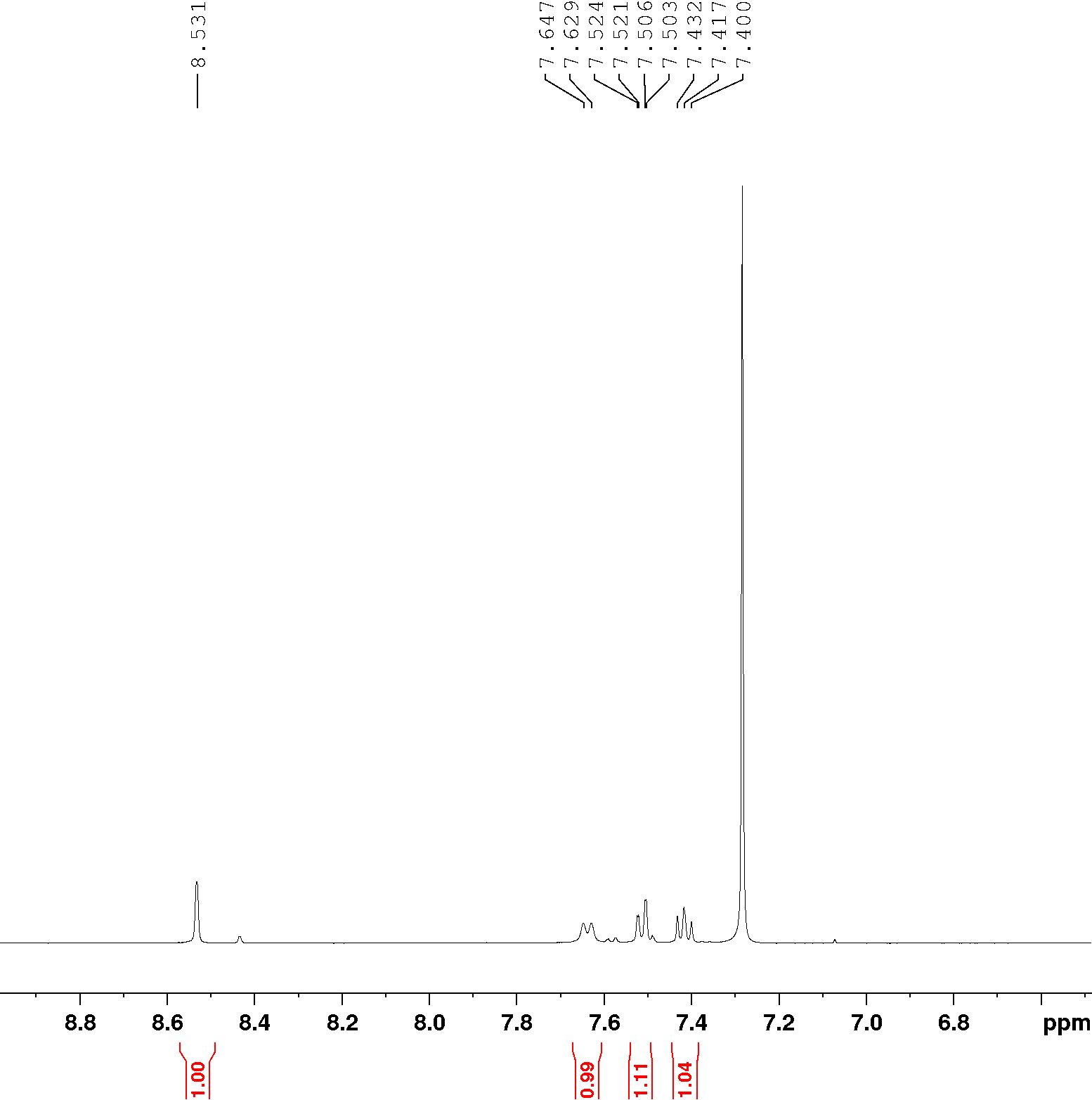


6

12

2

3


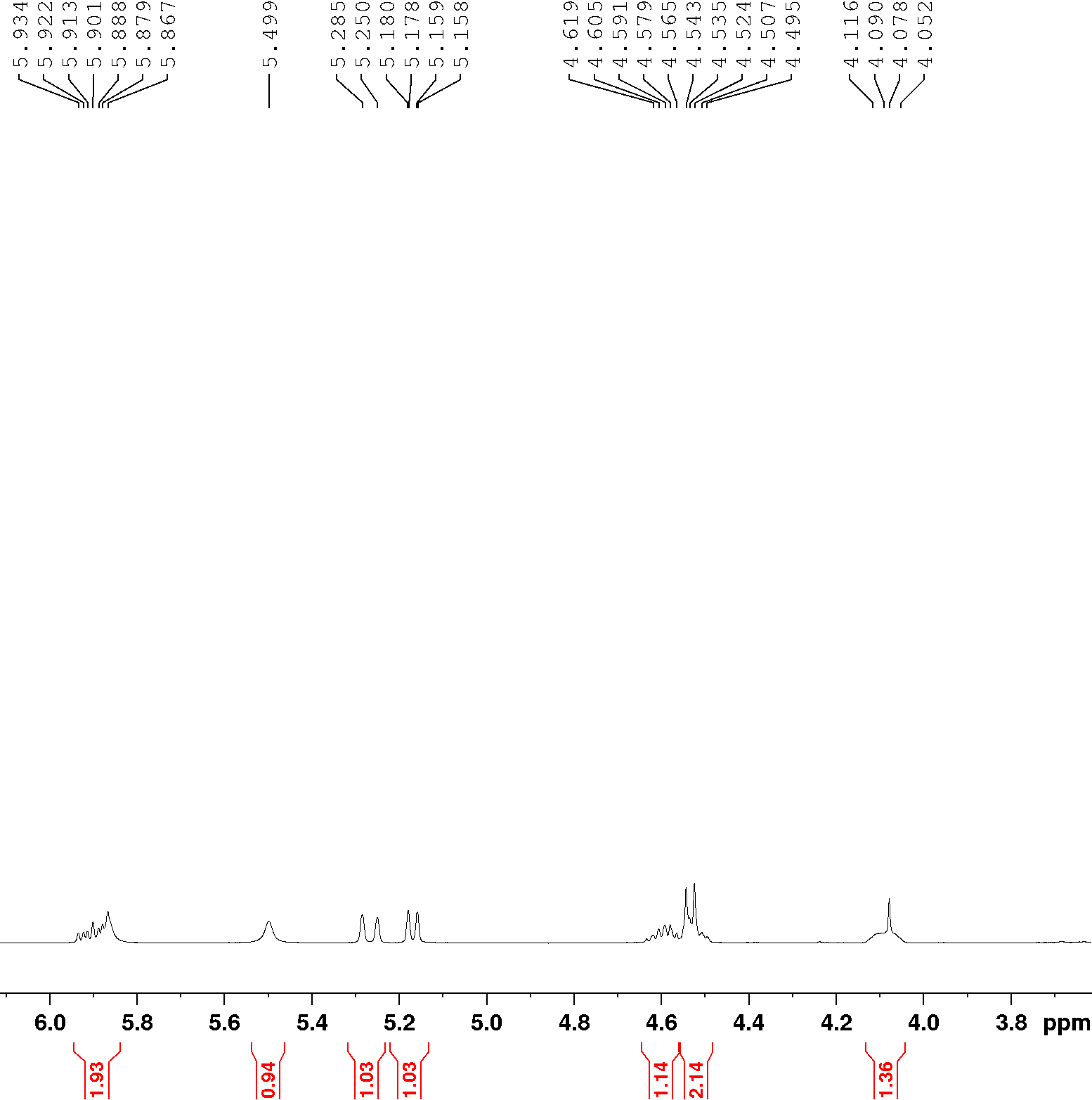


26

22

20

20

26

25

14

24


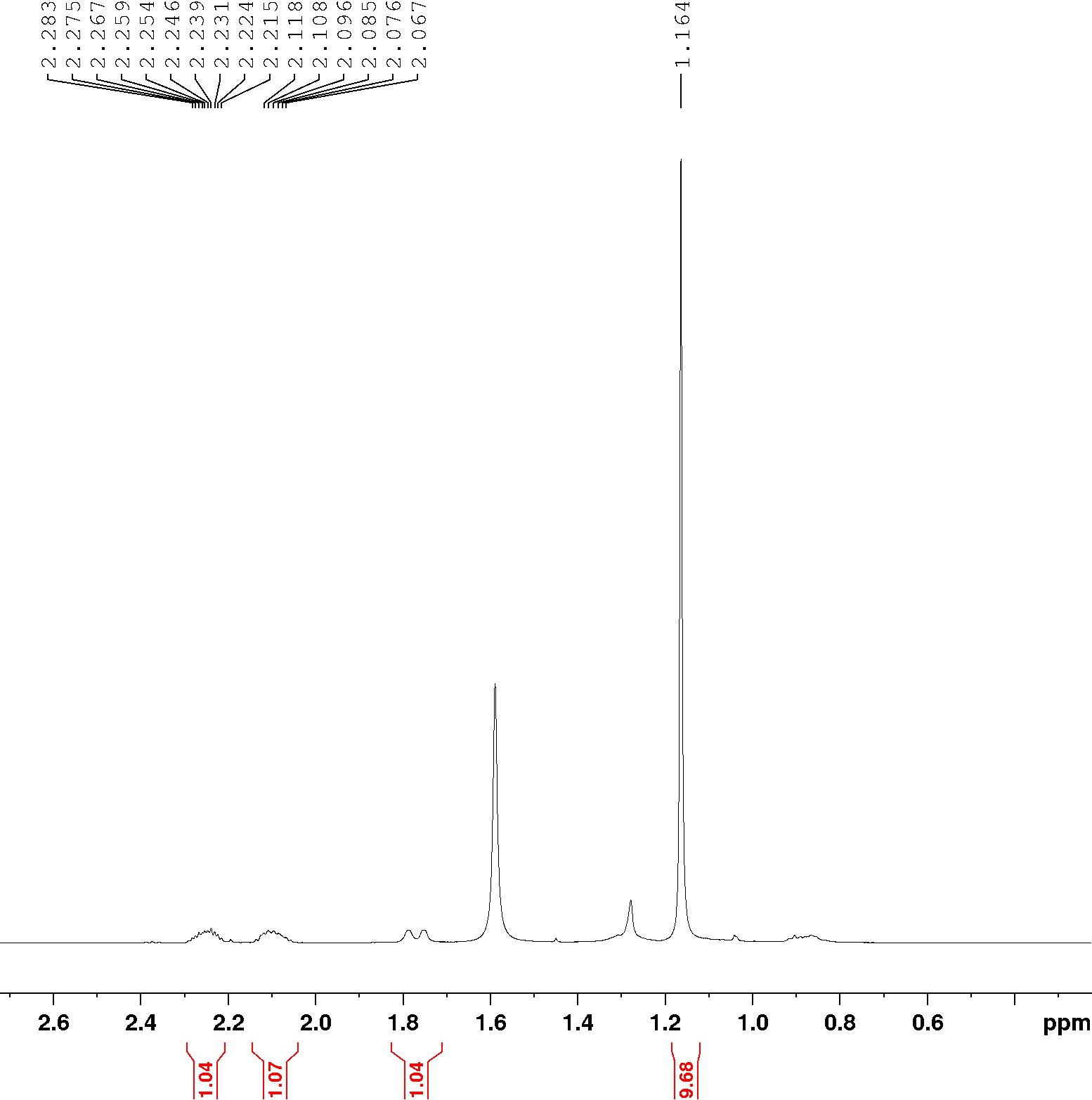


17, 18, 19

23

23


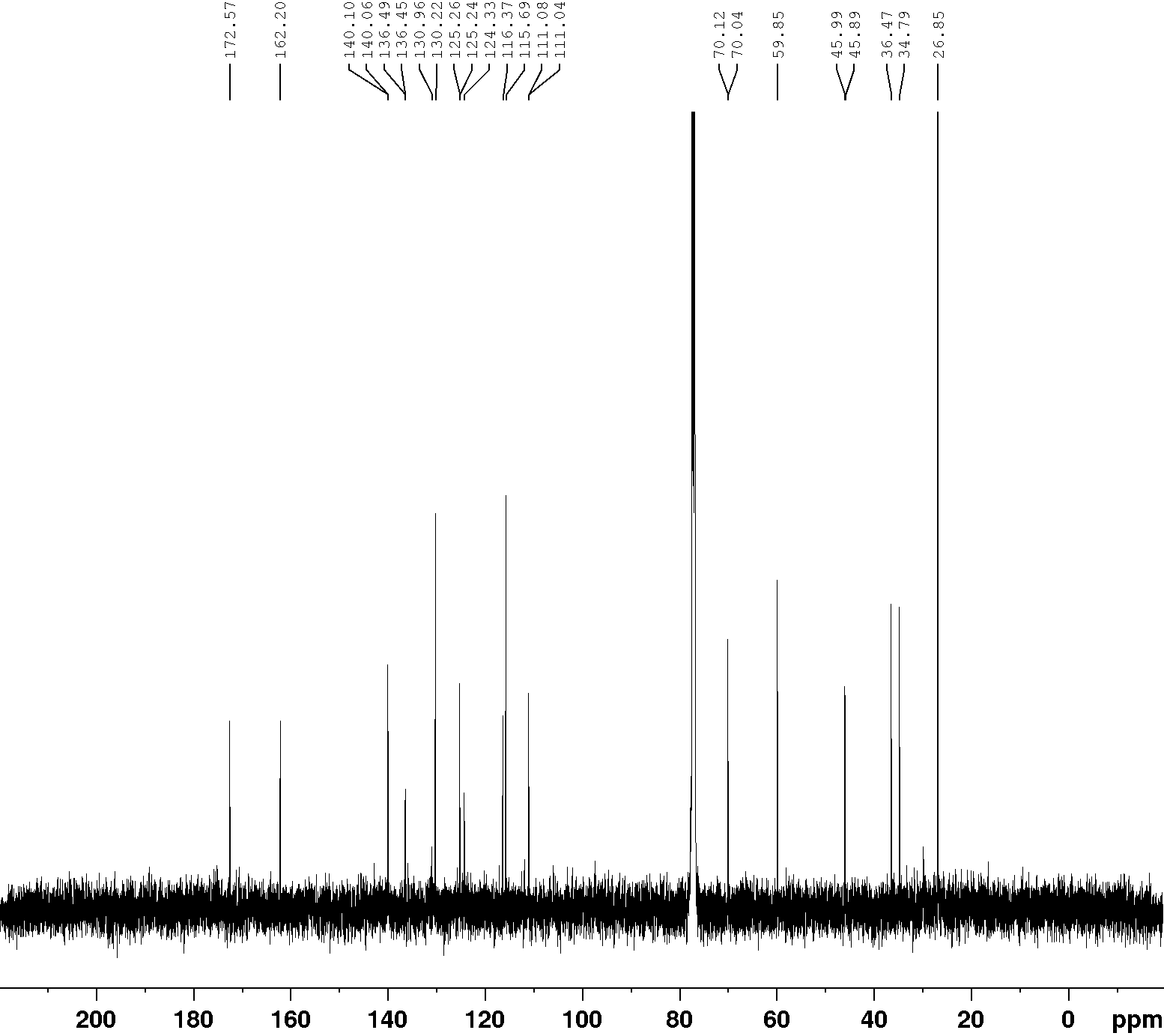

Supplement: Supplementary file 1 — Data S1. Supporting Information. [file DTA-17-701-s002.docx]
