# Supplementary material for: In vitro metabolism study of ADB‐P‐5Br‐INACA and ADB‐4en‐P‐5Br‐INACA using human hepatocytes, liver microsomes, and in‐house synthesized references
Source: Drug Test Anal. 2024 Jul 23;17(5):701–12. doi: 10.1002/dta.3773 (PMC12012409; doi:10.1002/dta.3773)

## Slide 1
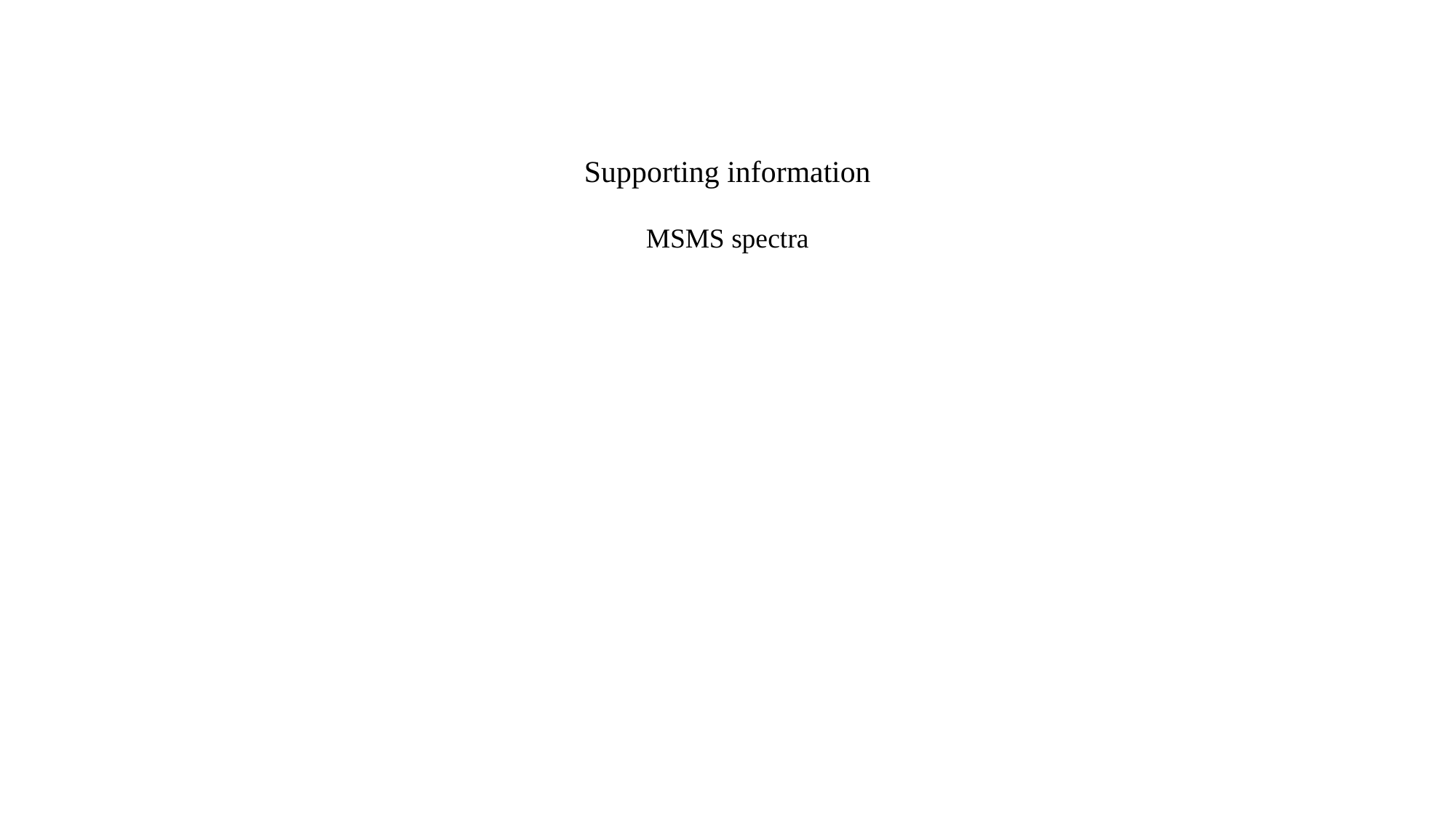

Supporting information
MSMS spectra

## Slide 2
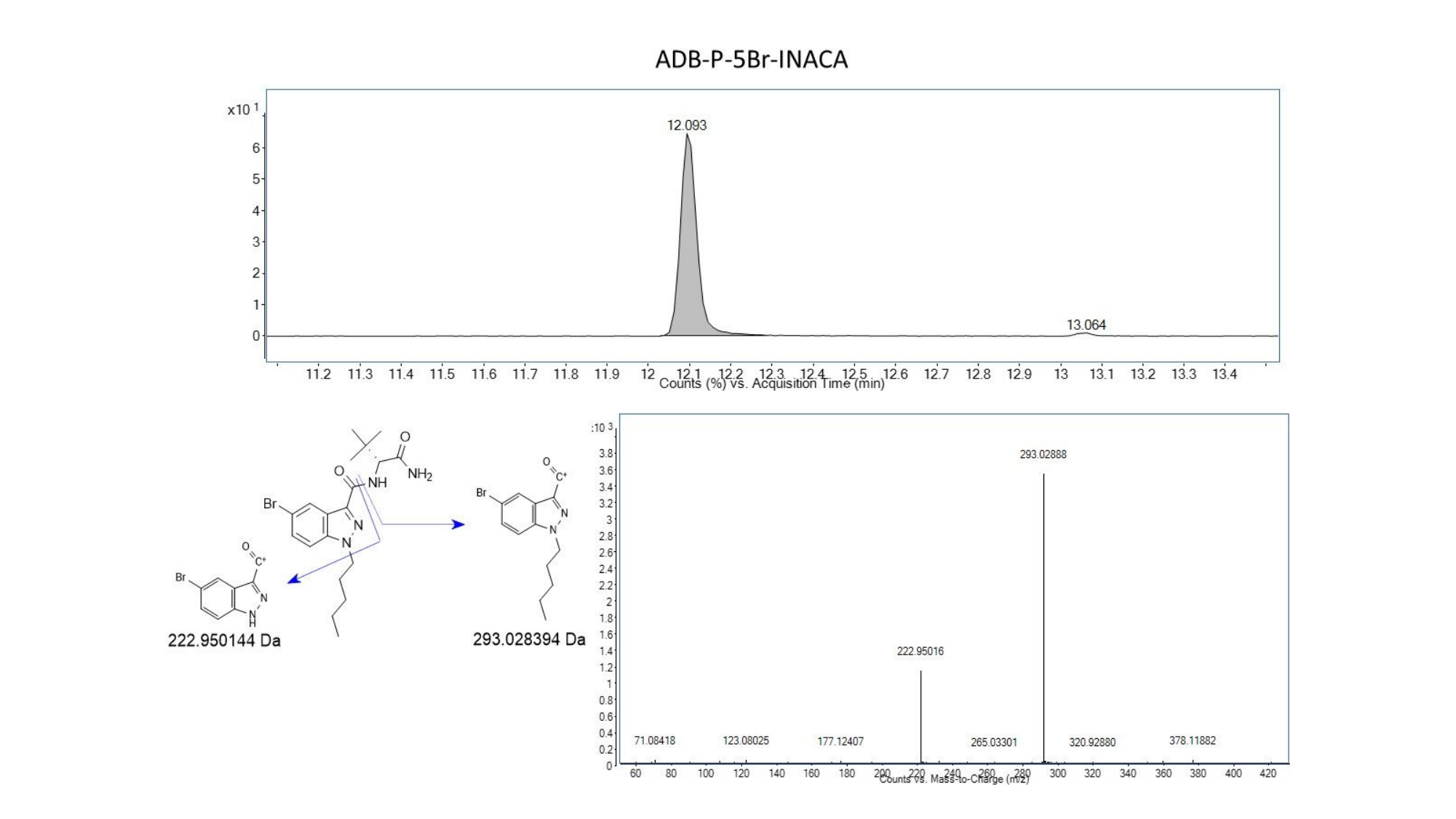

## Slide 3
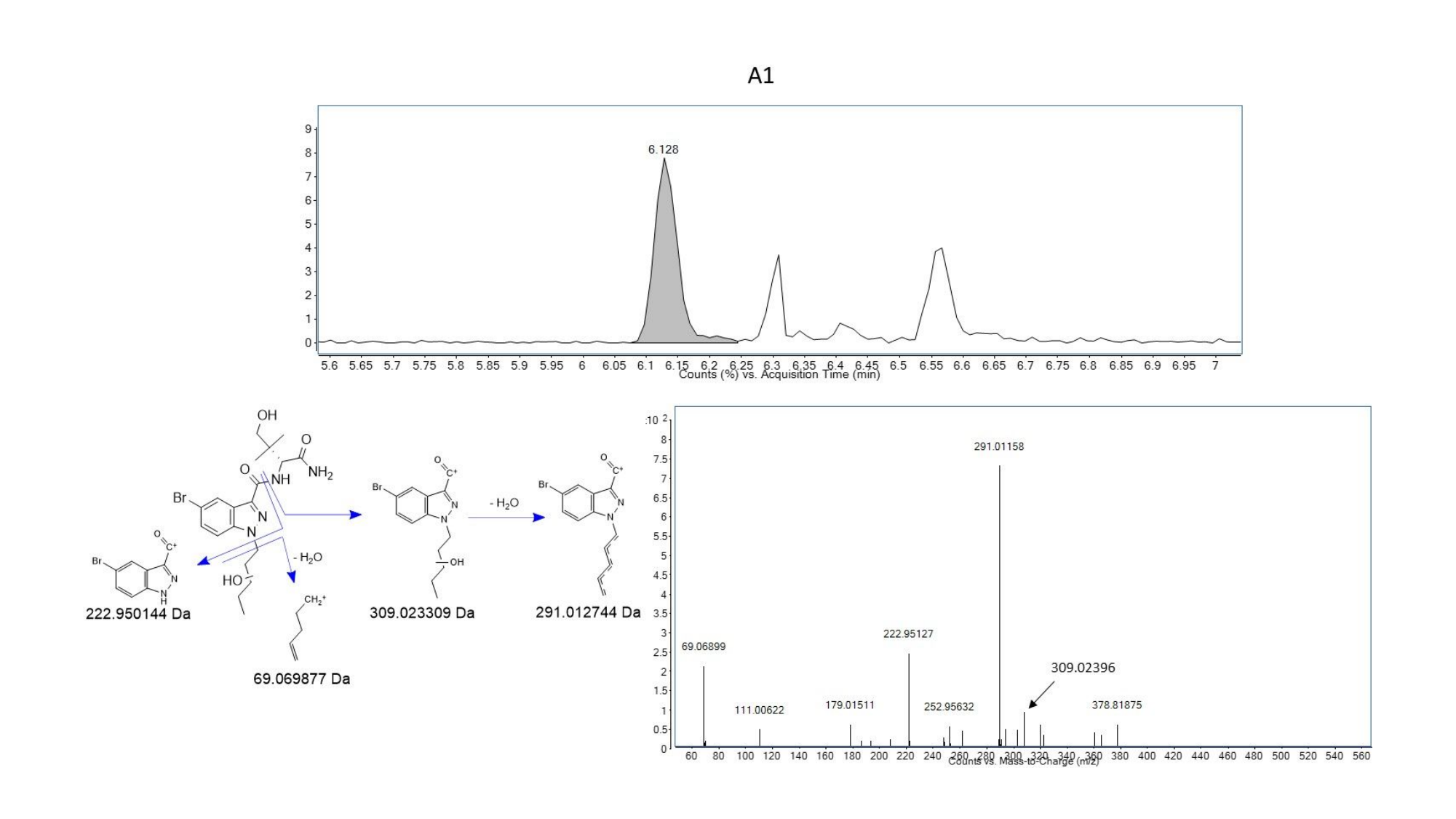

## Slide 4
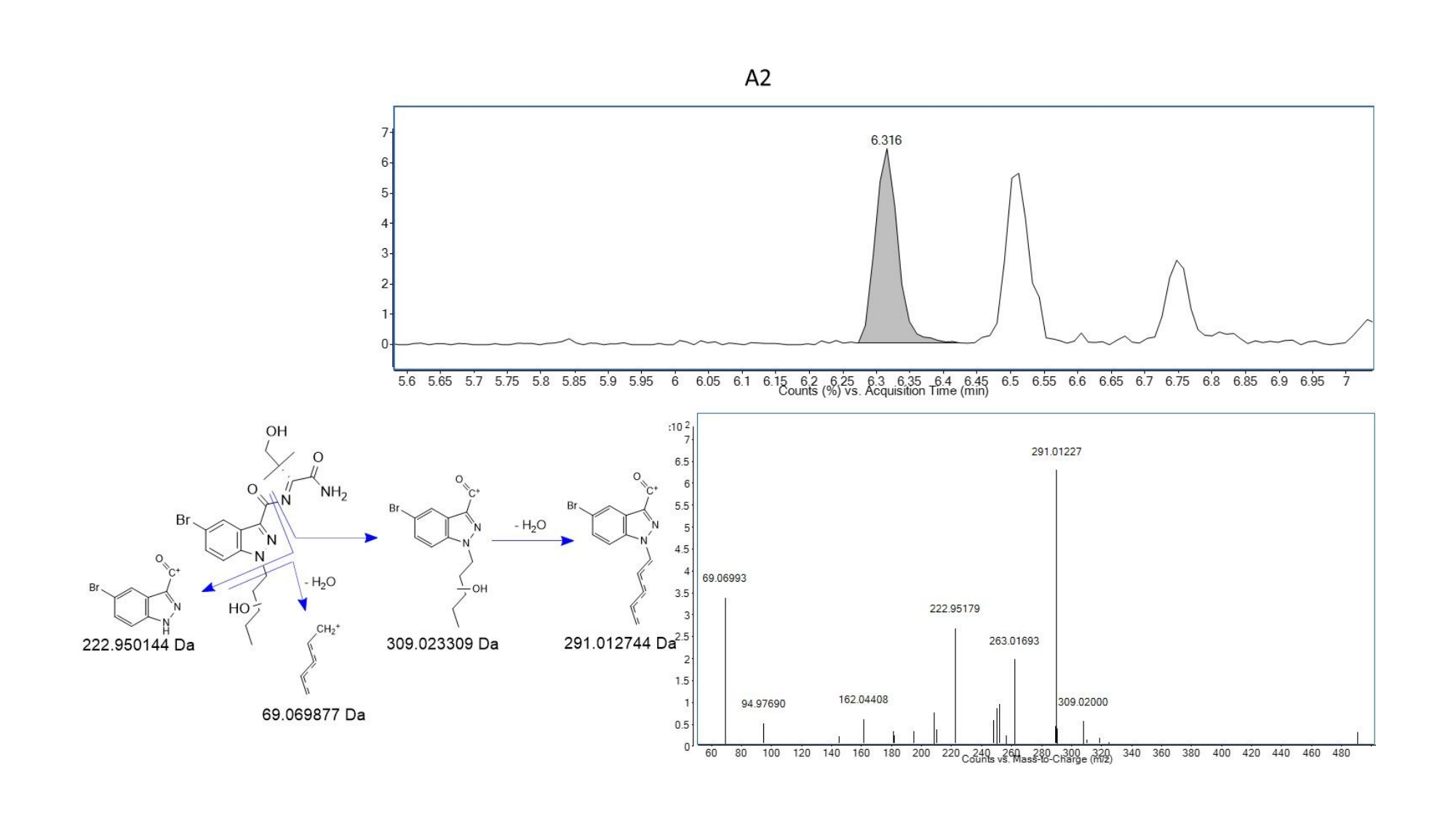

## Slide 5
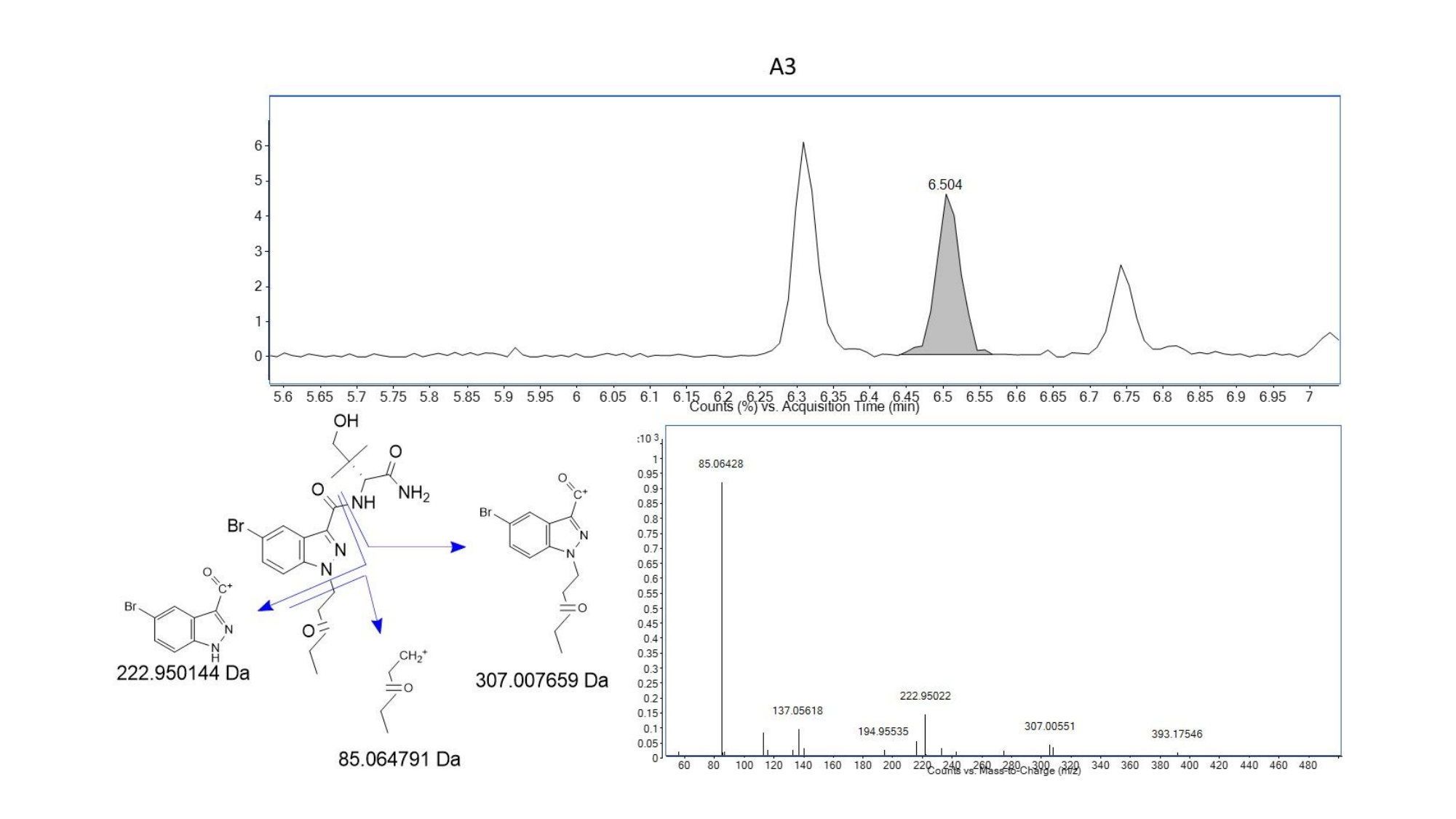

## Slide 6
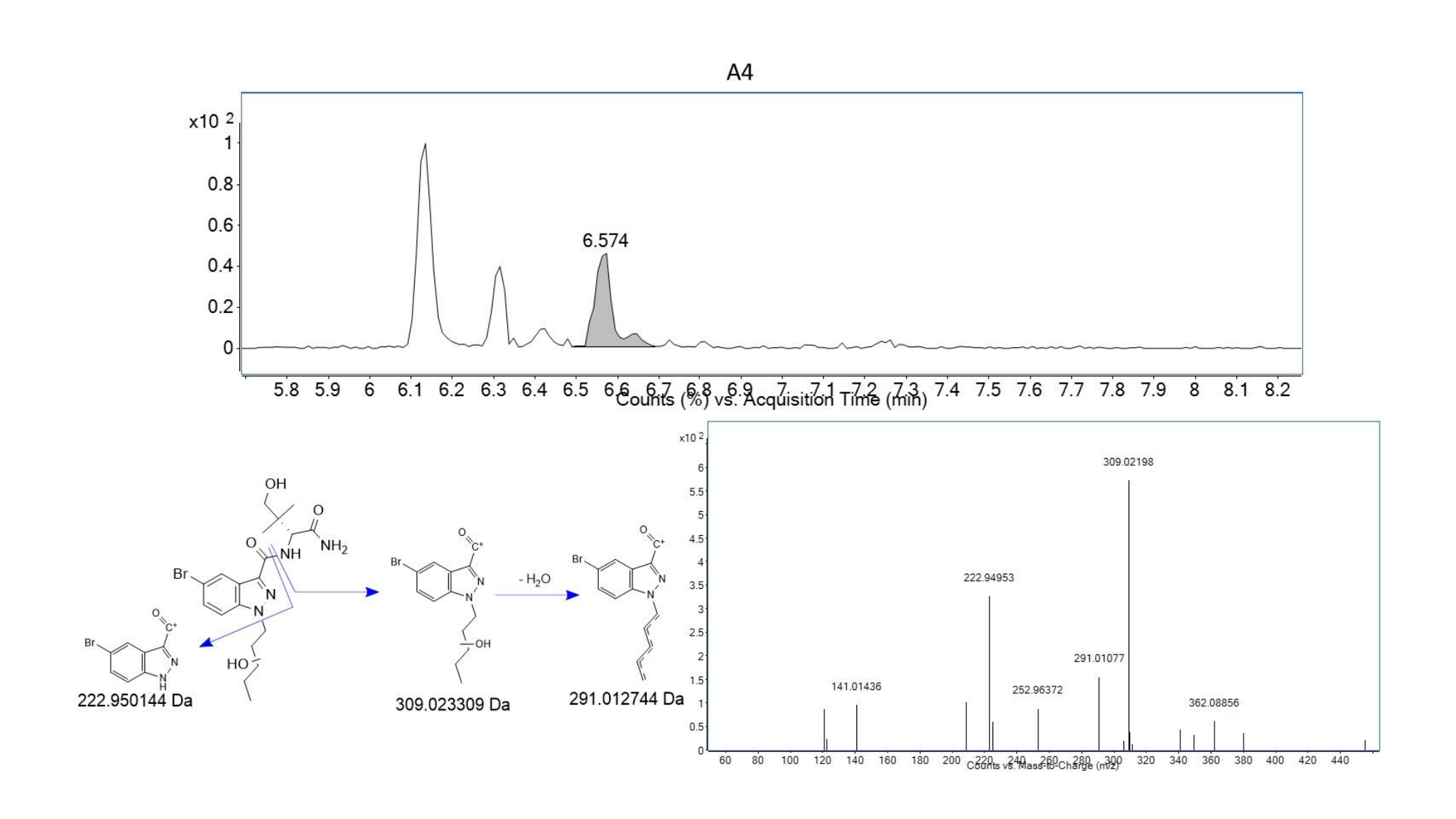

## Slide 7
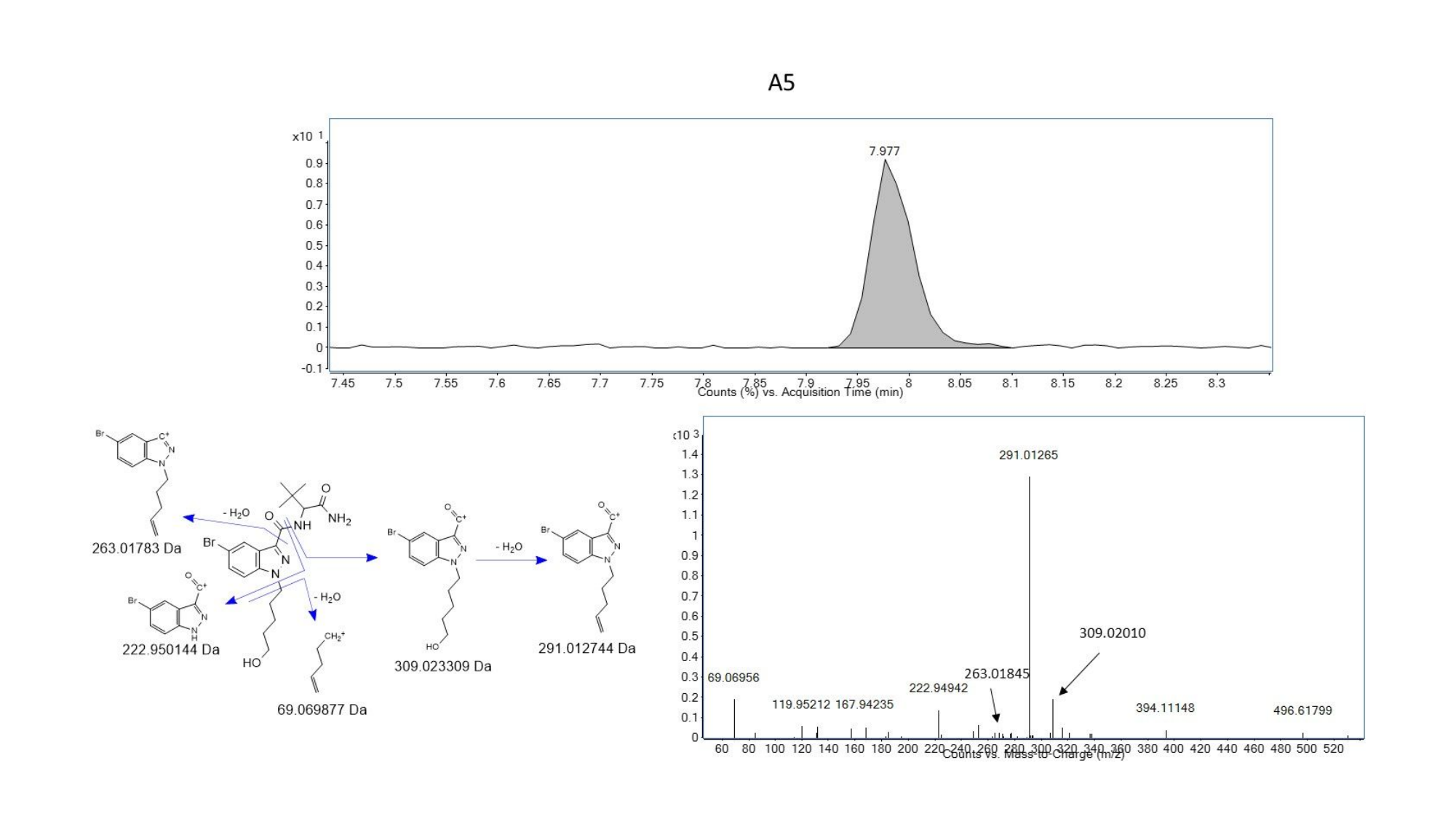

## Slide 8
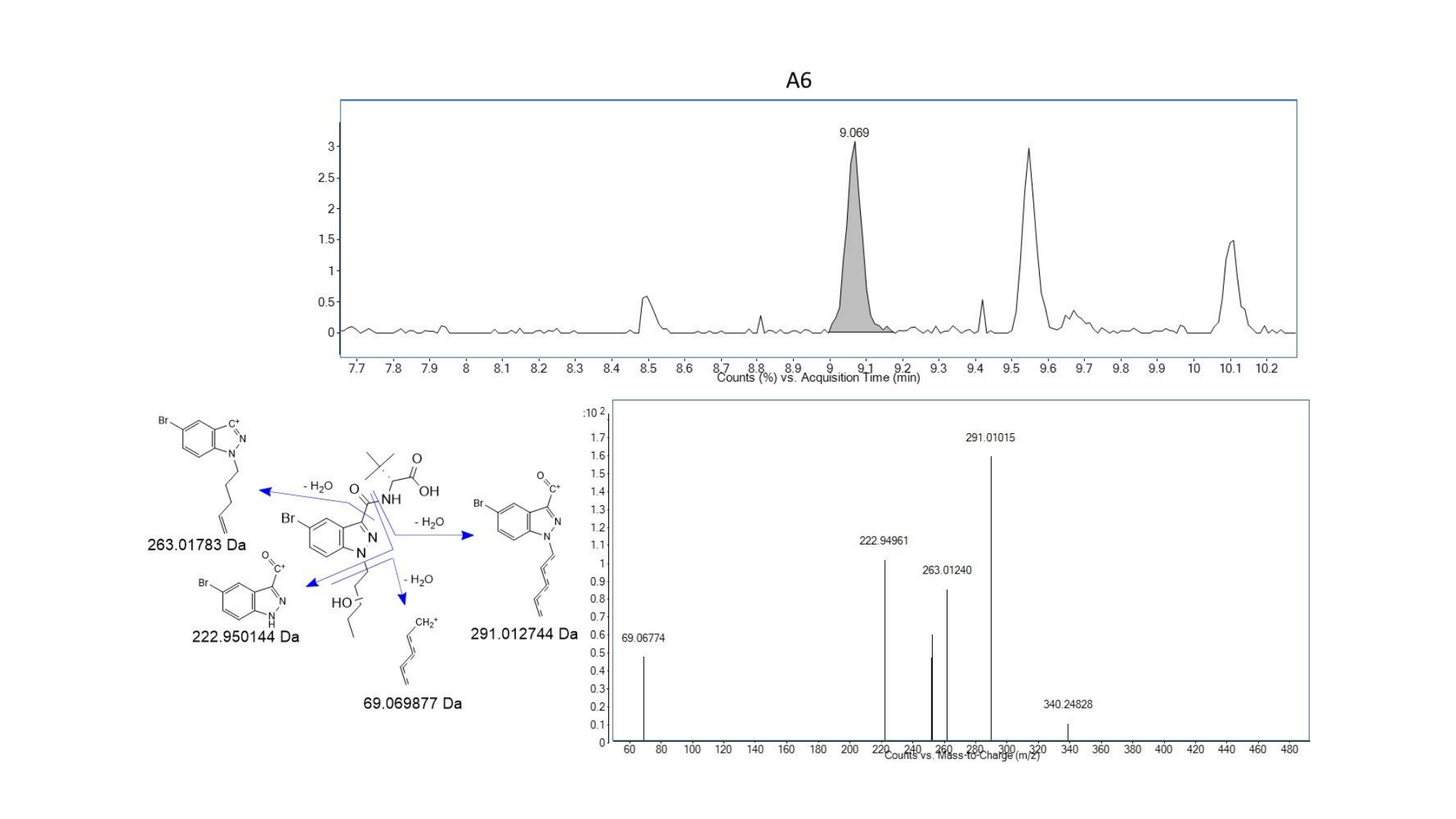

## Slide 9
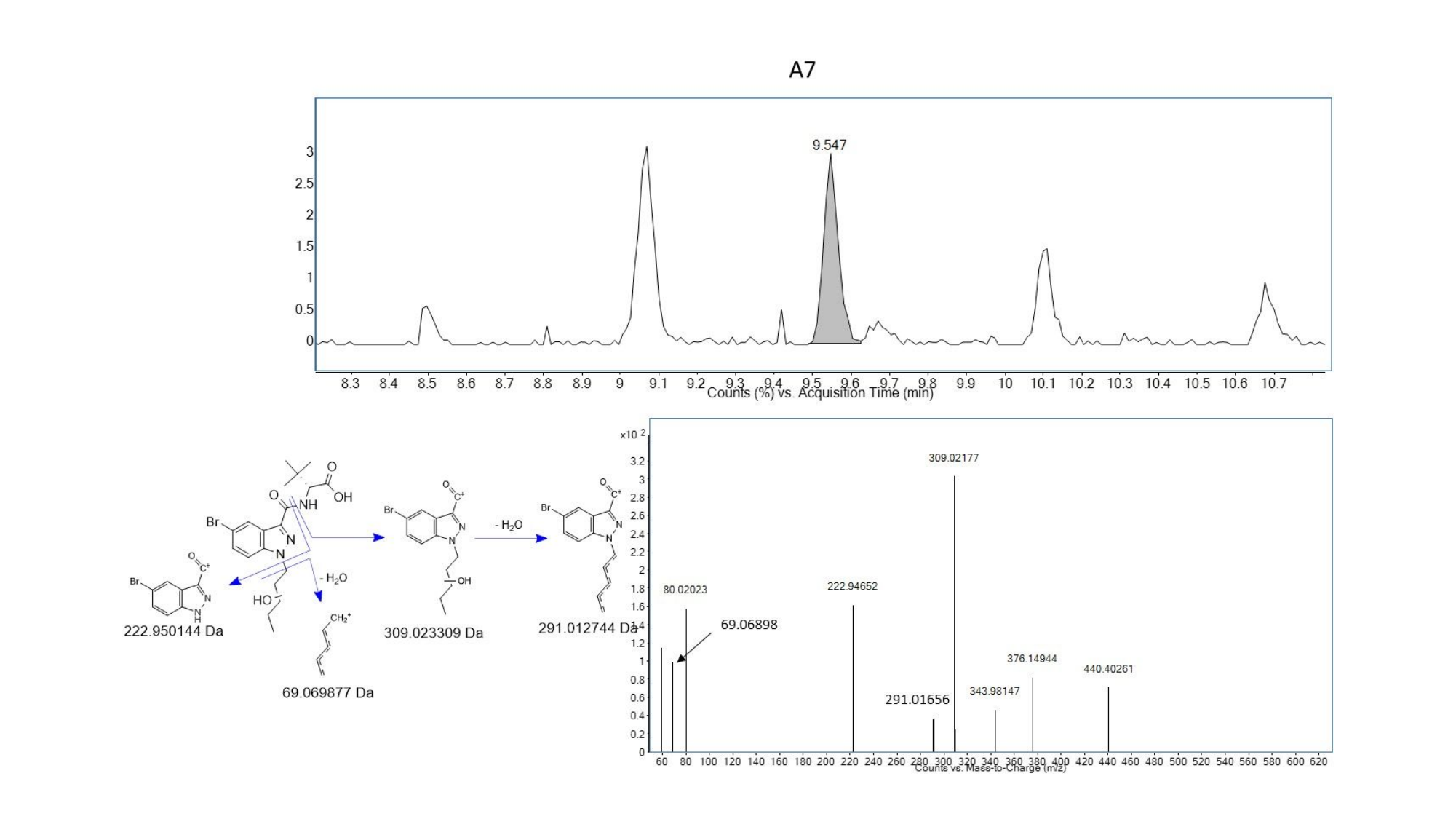

## Slide 10
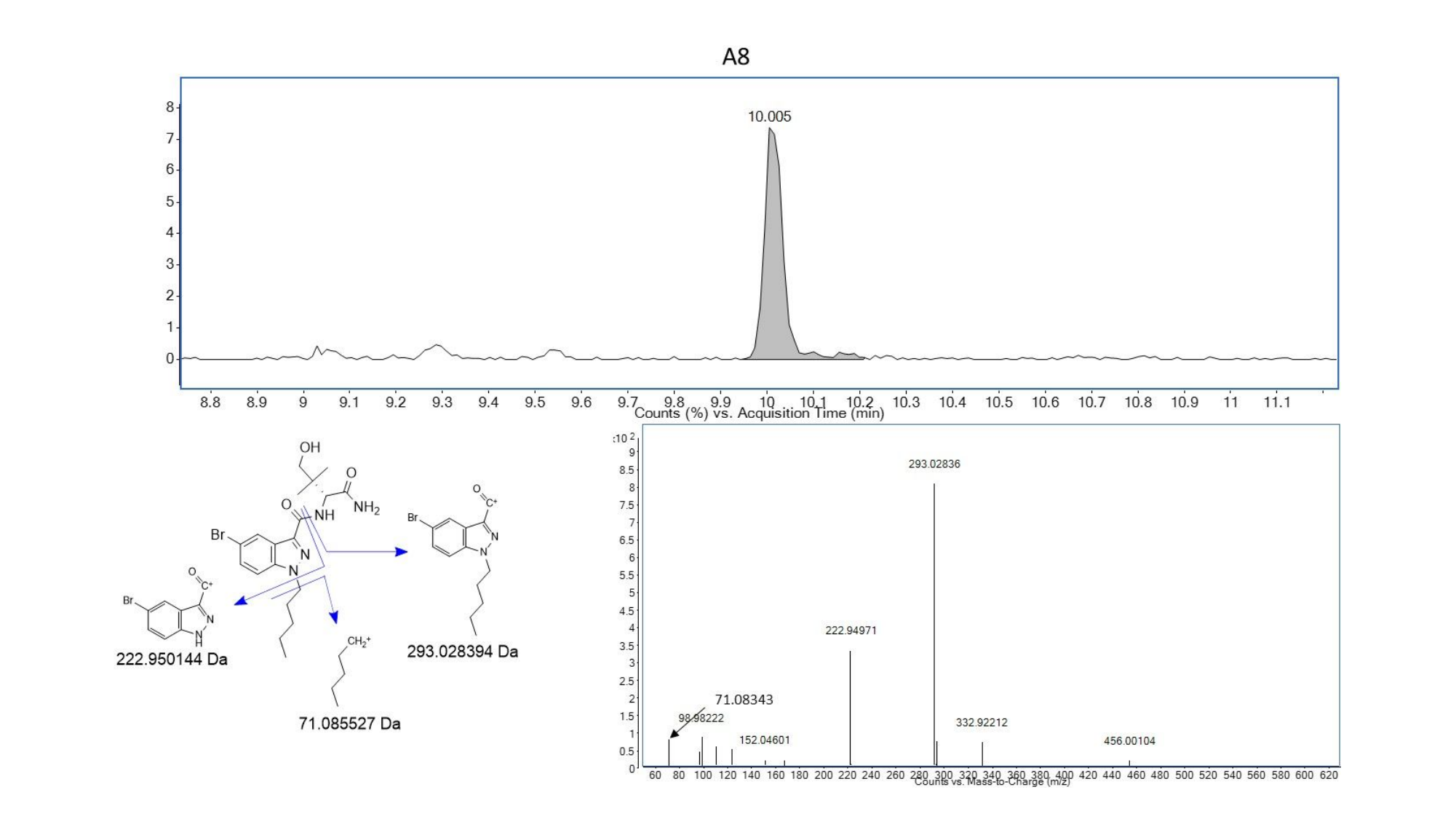

## Slide 11
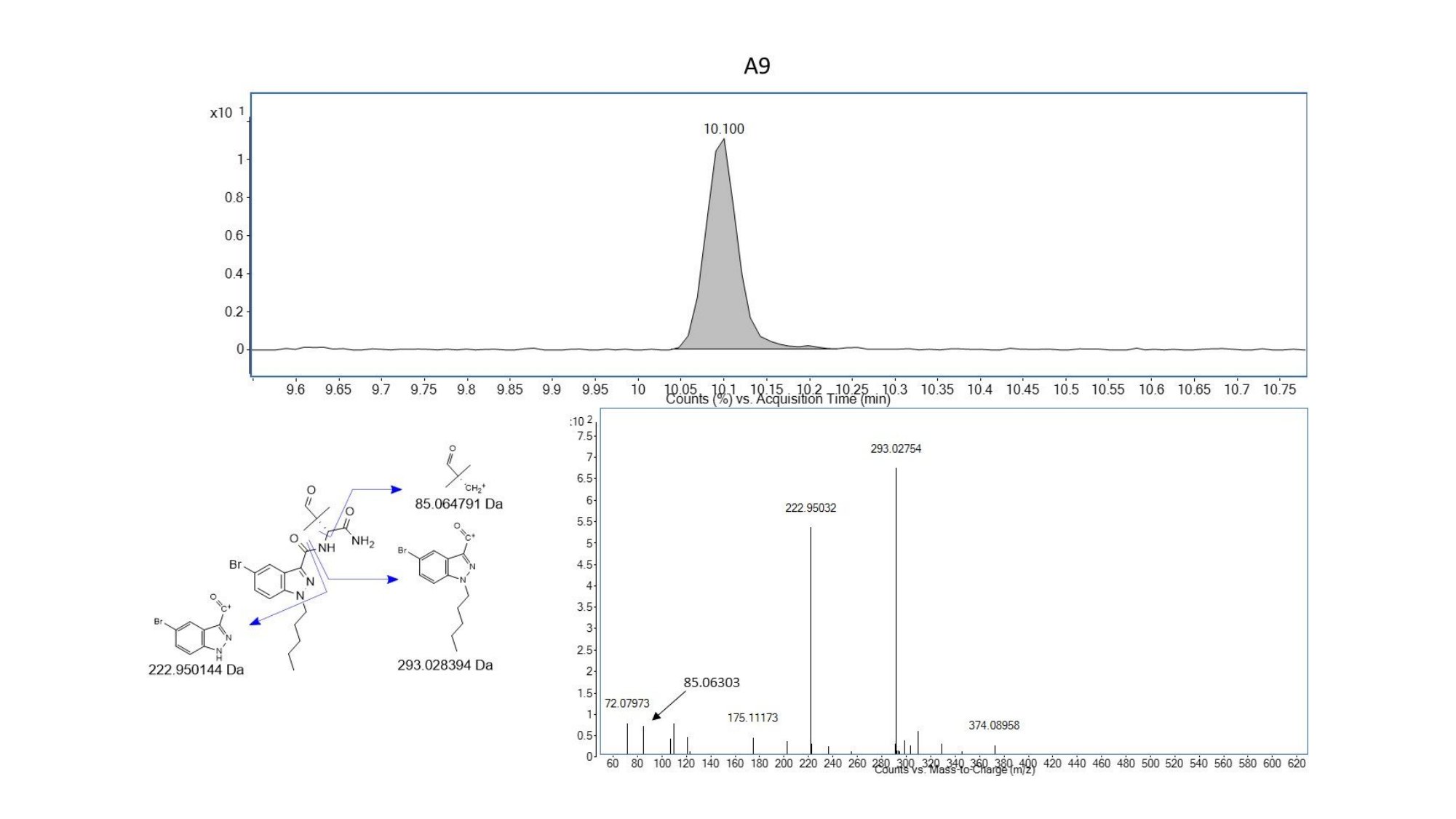

## Slide 12
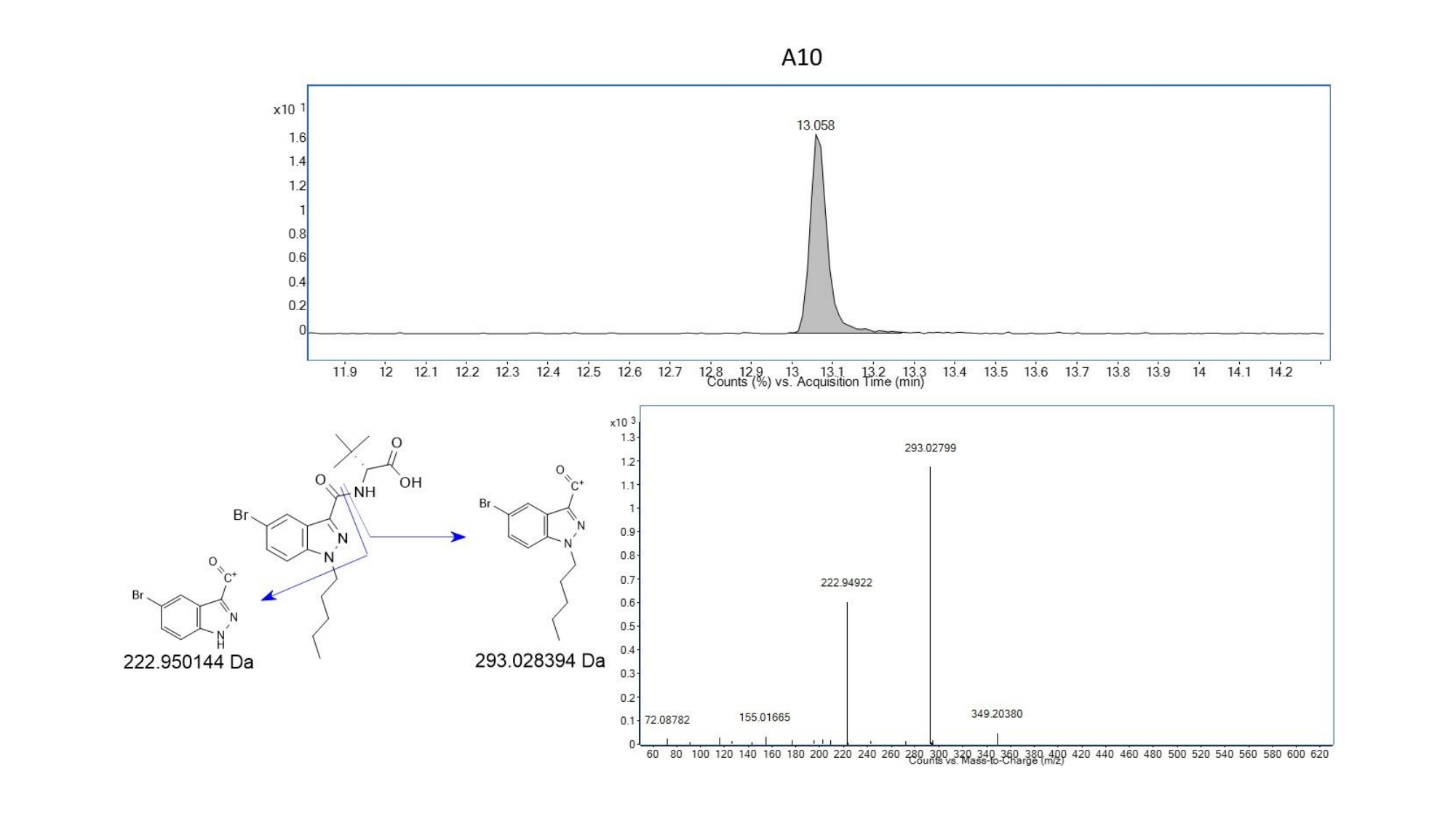

## Slide 13
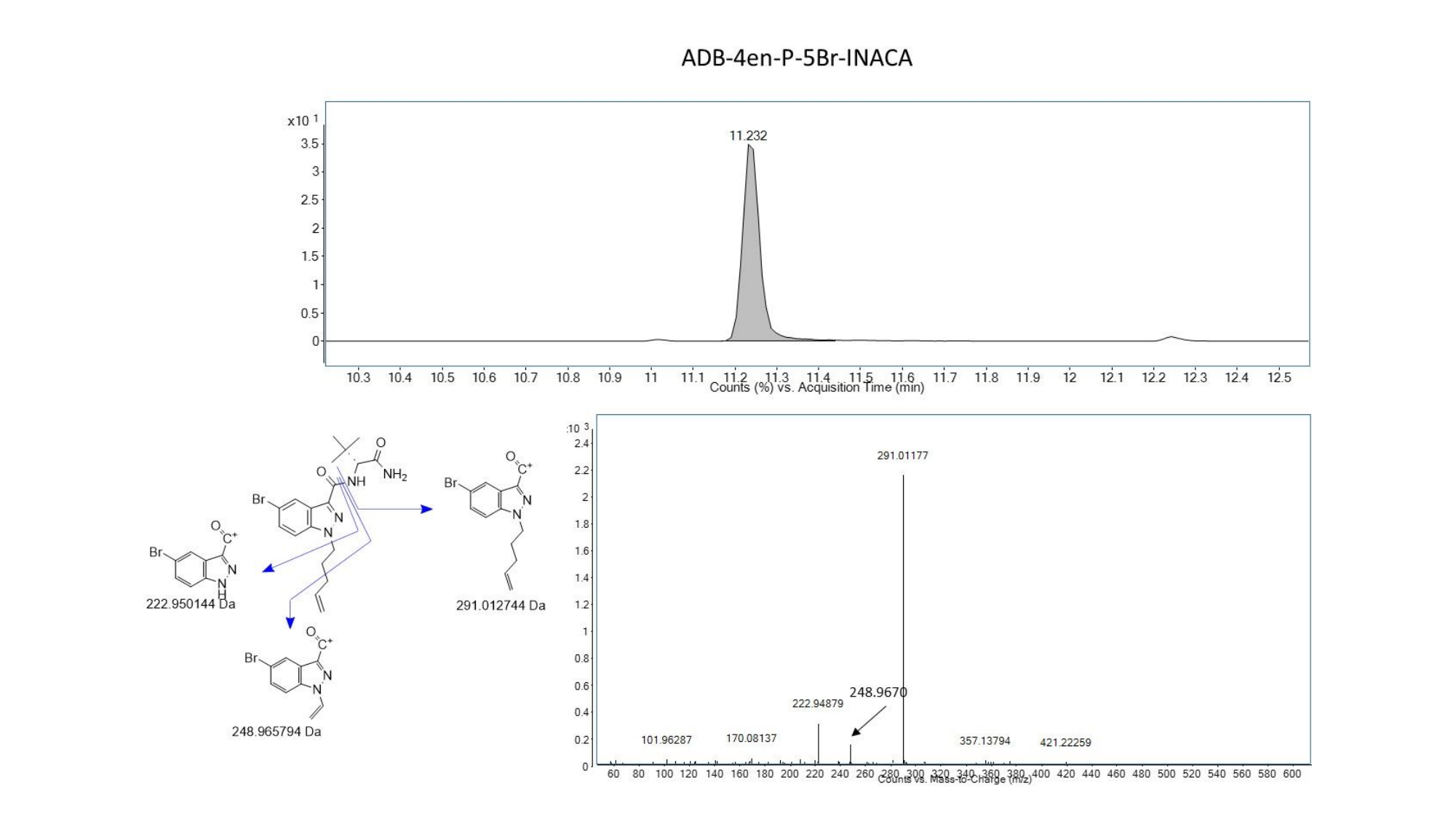

## Slide 14
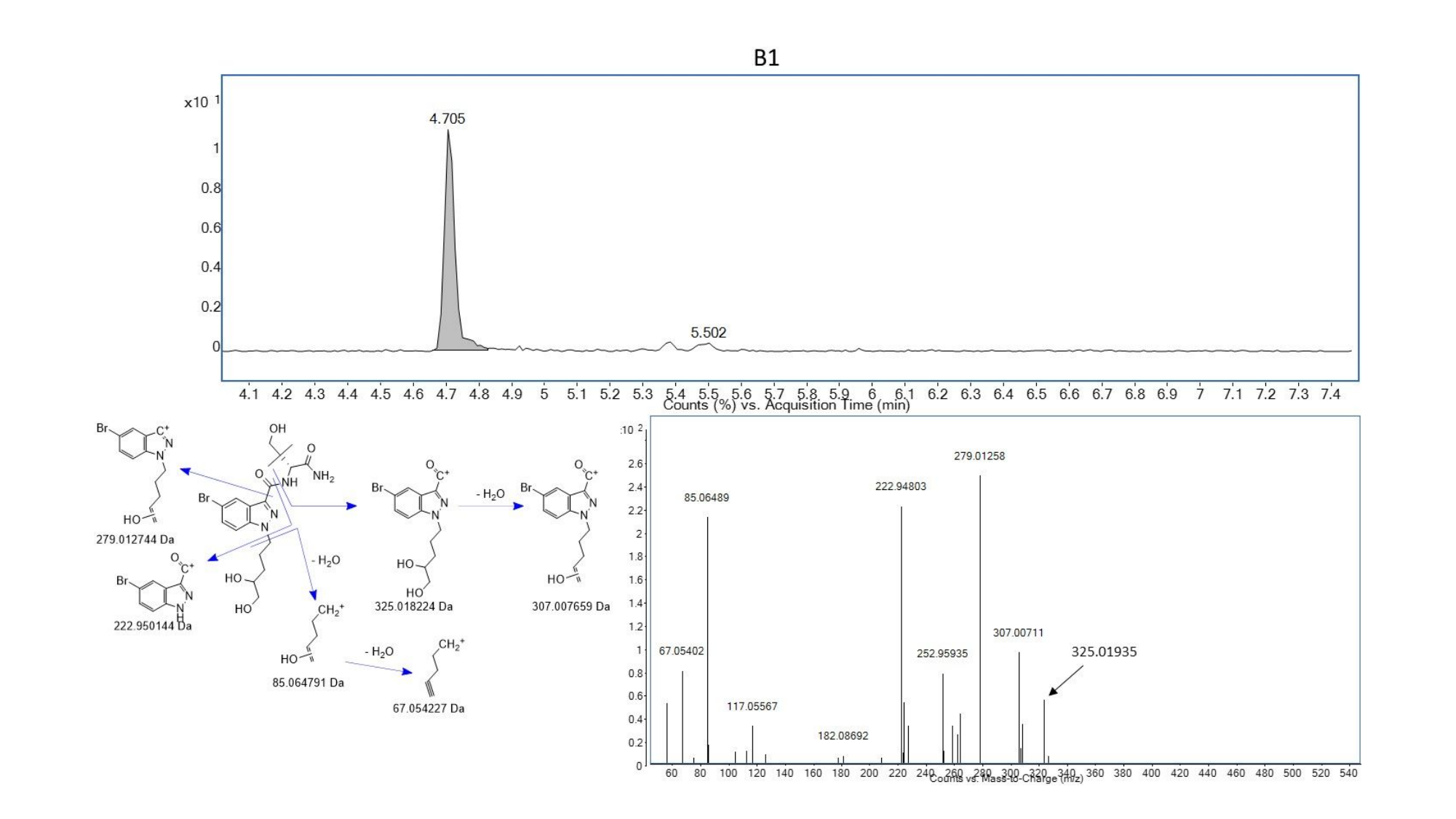

## Slide 15
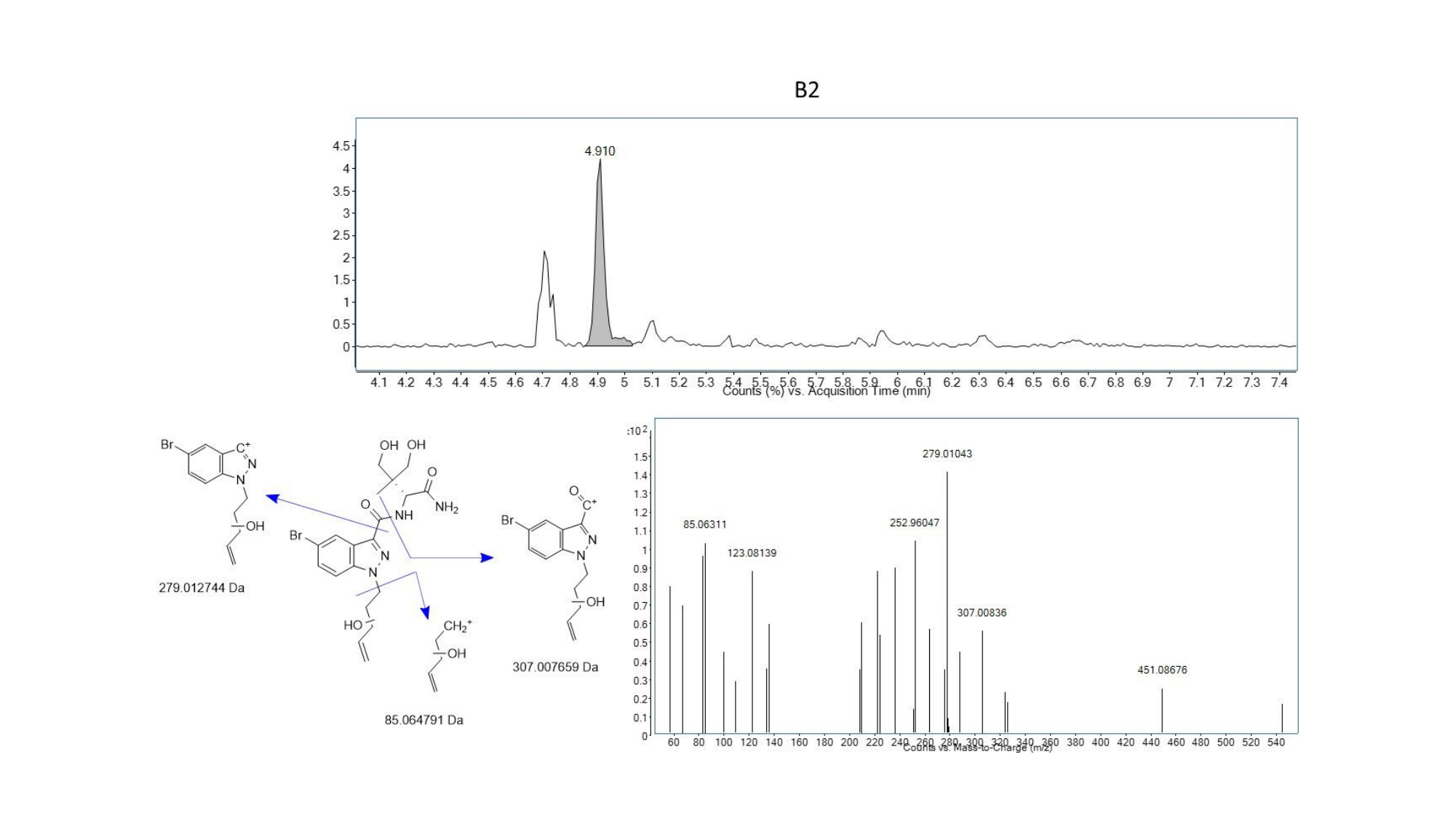

## Slide 16
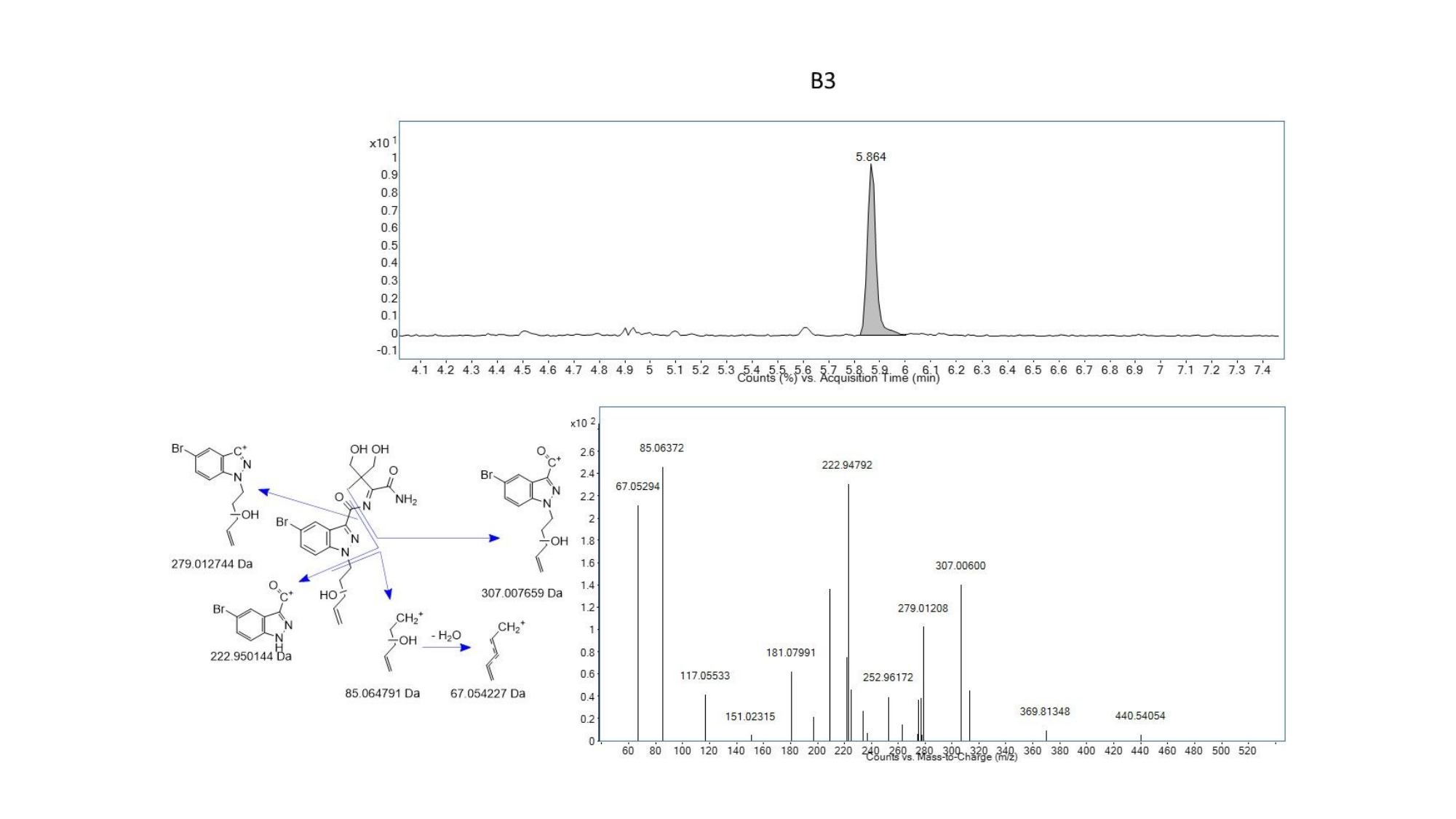

## Slide 17
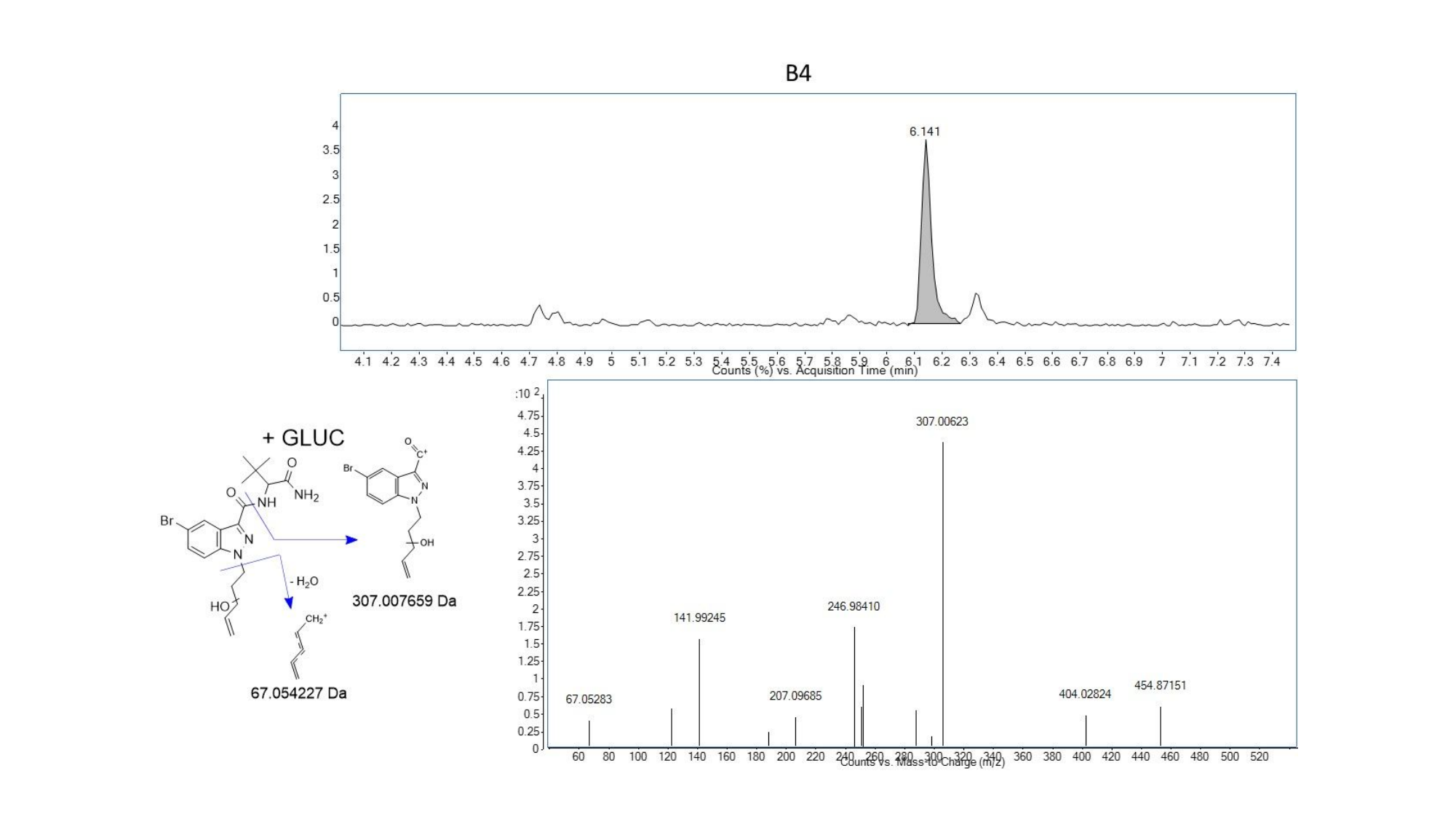

## Slide 18
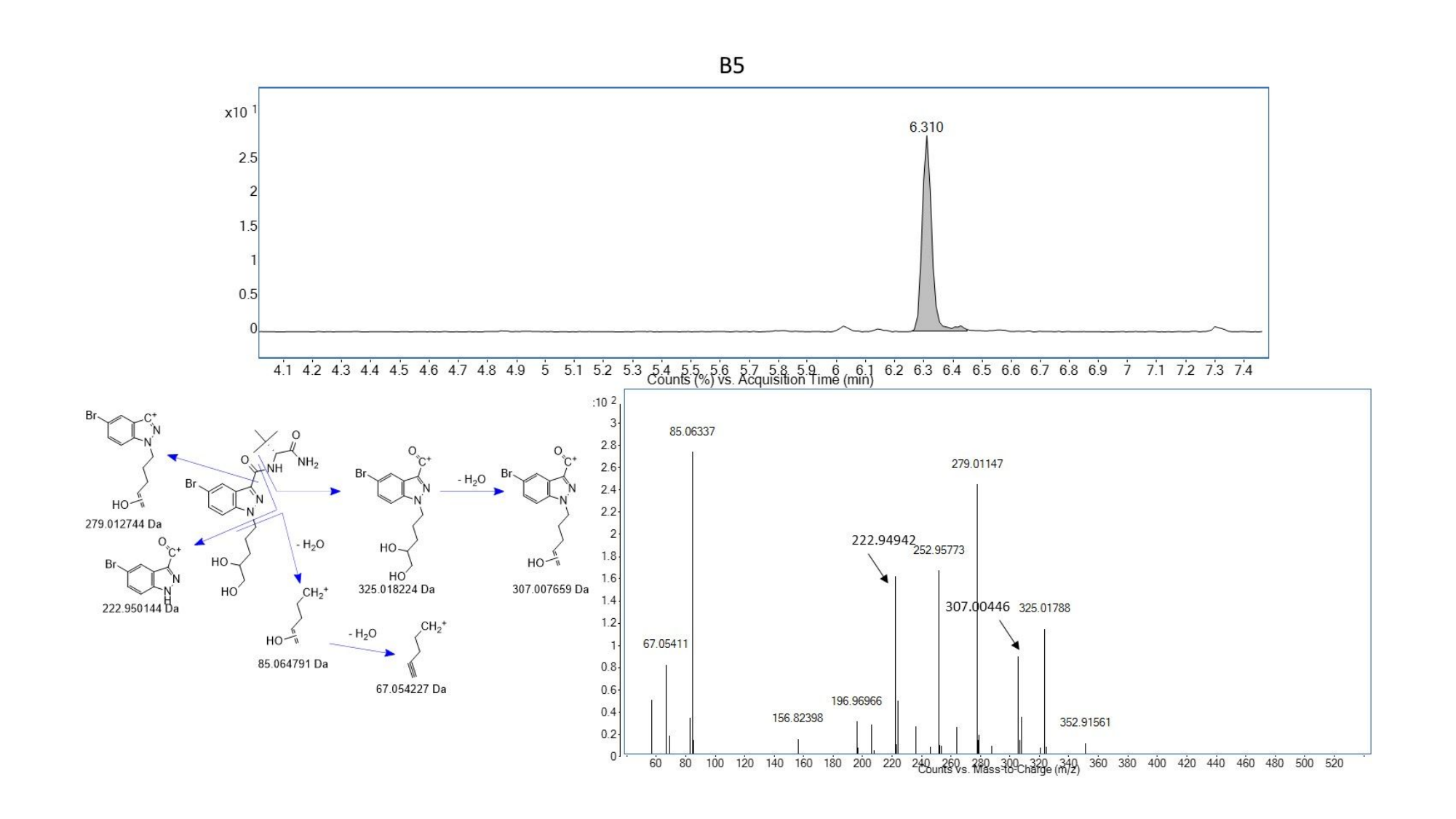

## Slide 19
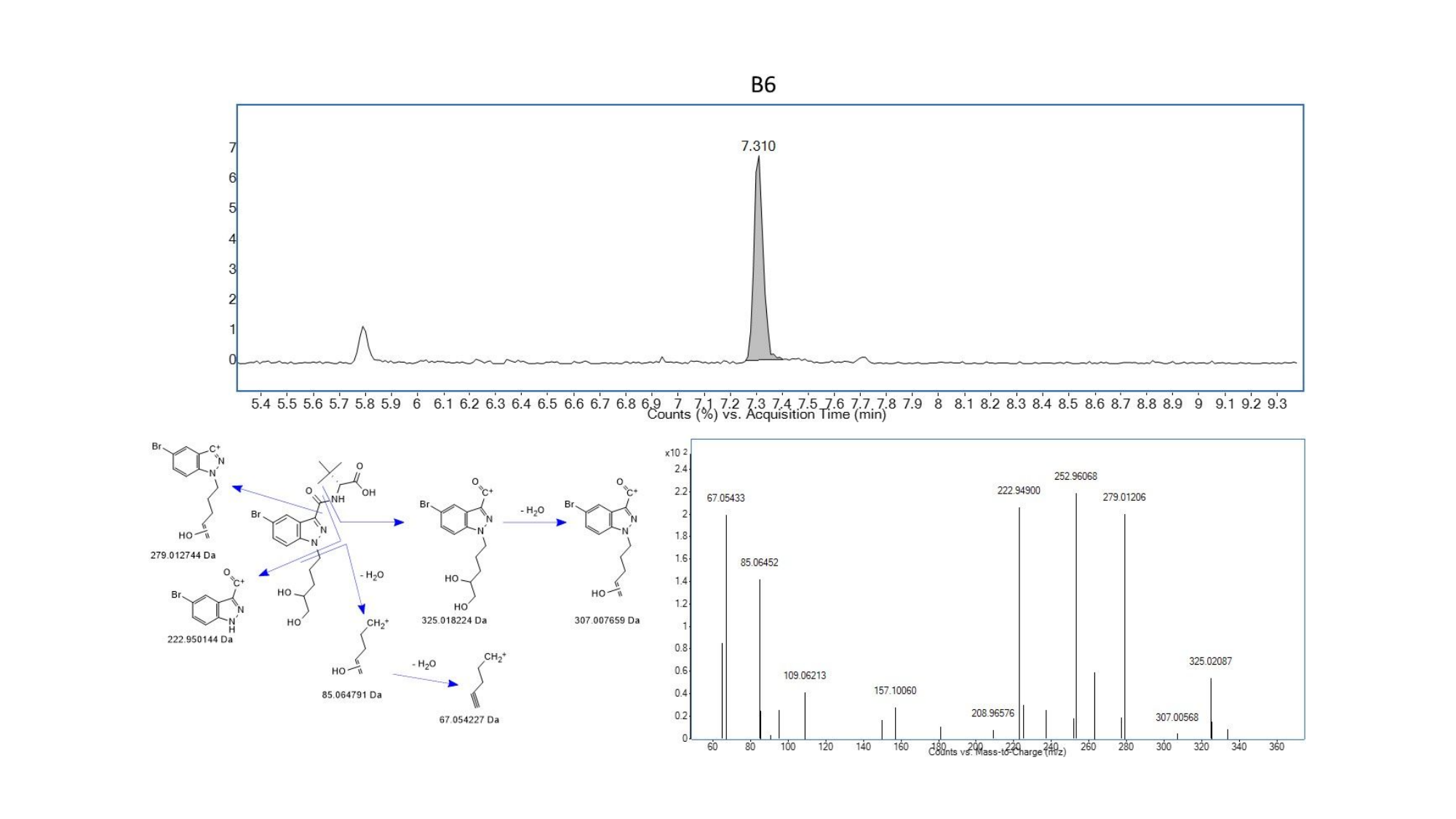

## Slide 20
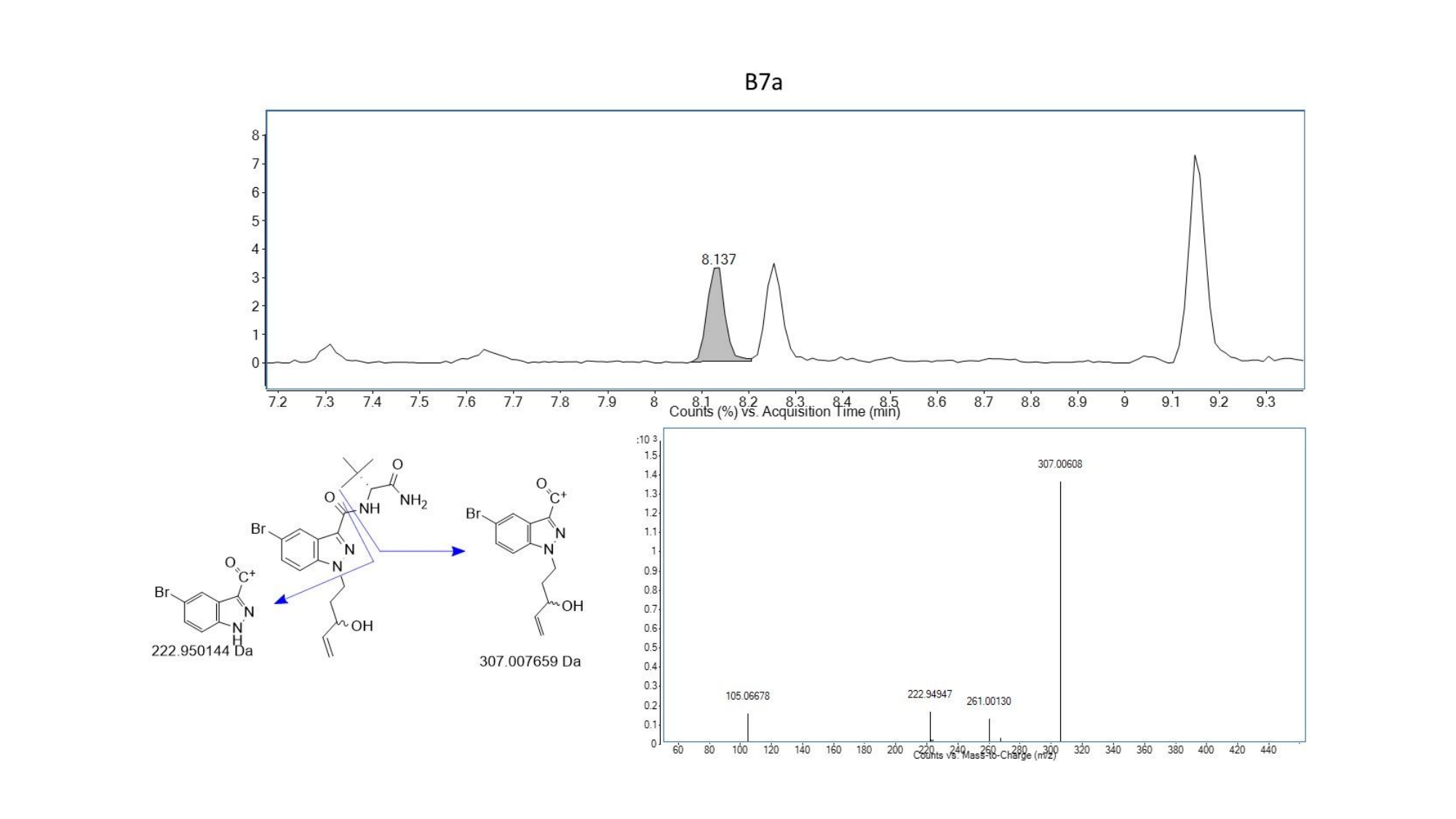

## Slide 21
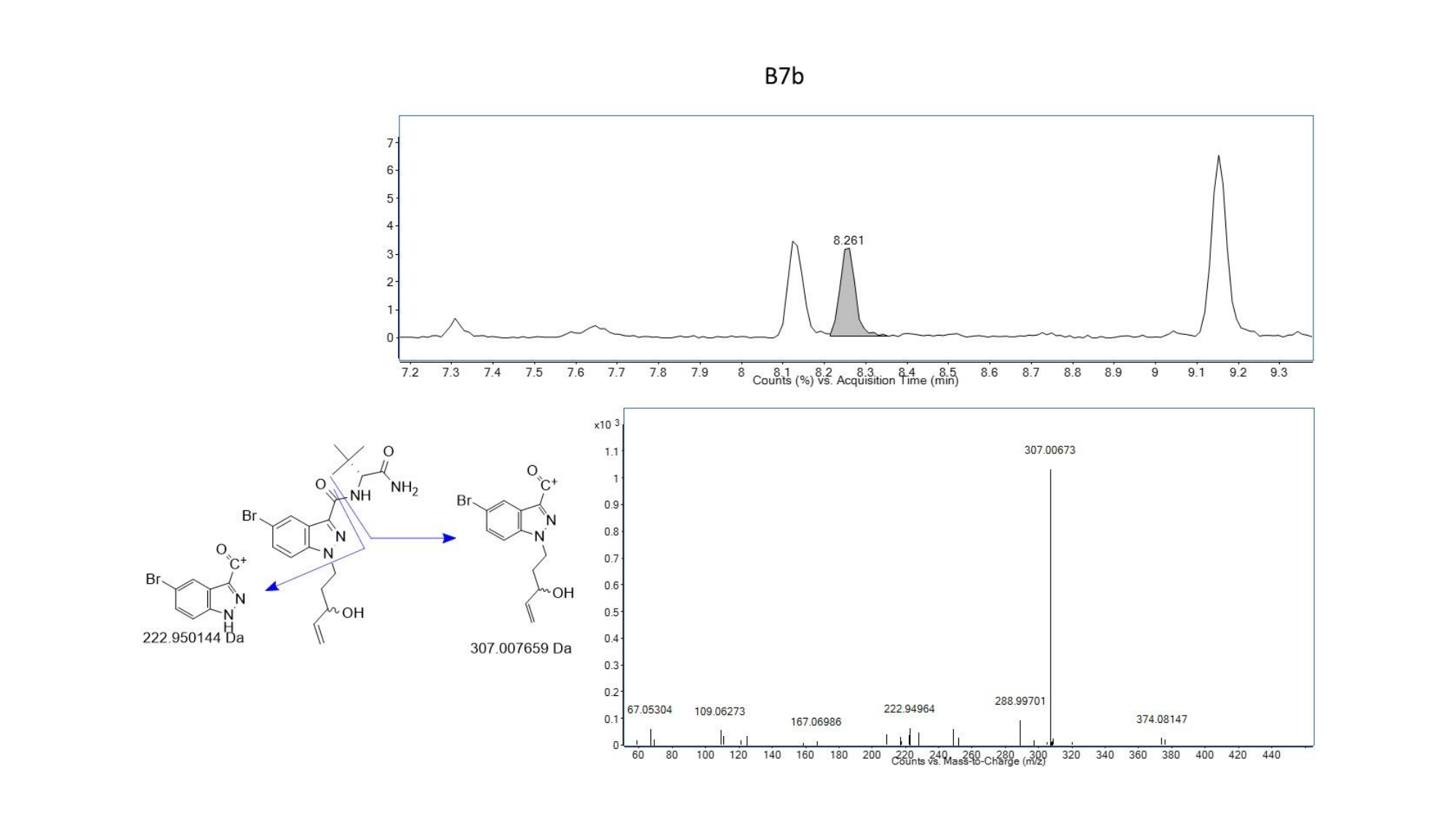

## Slide 22
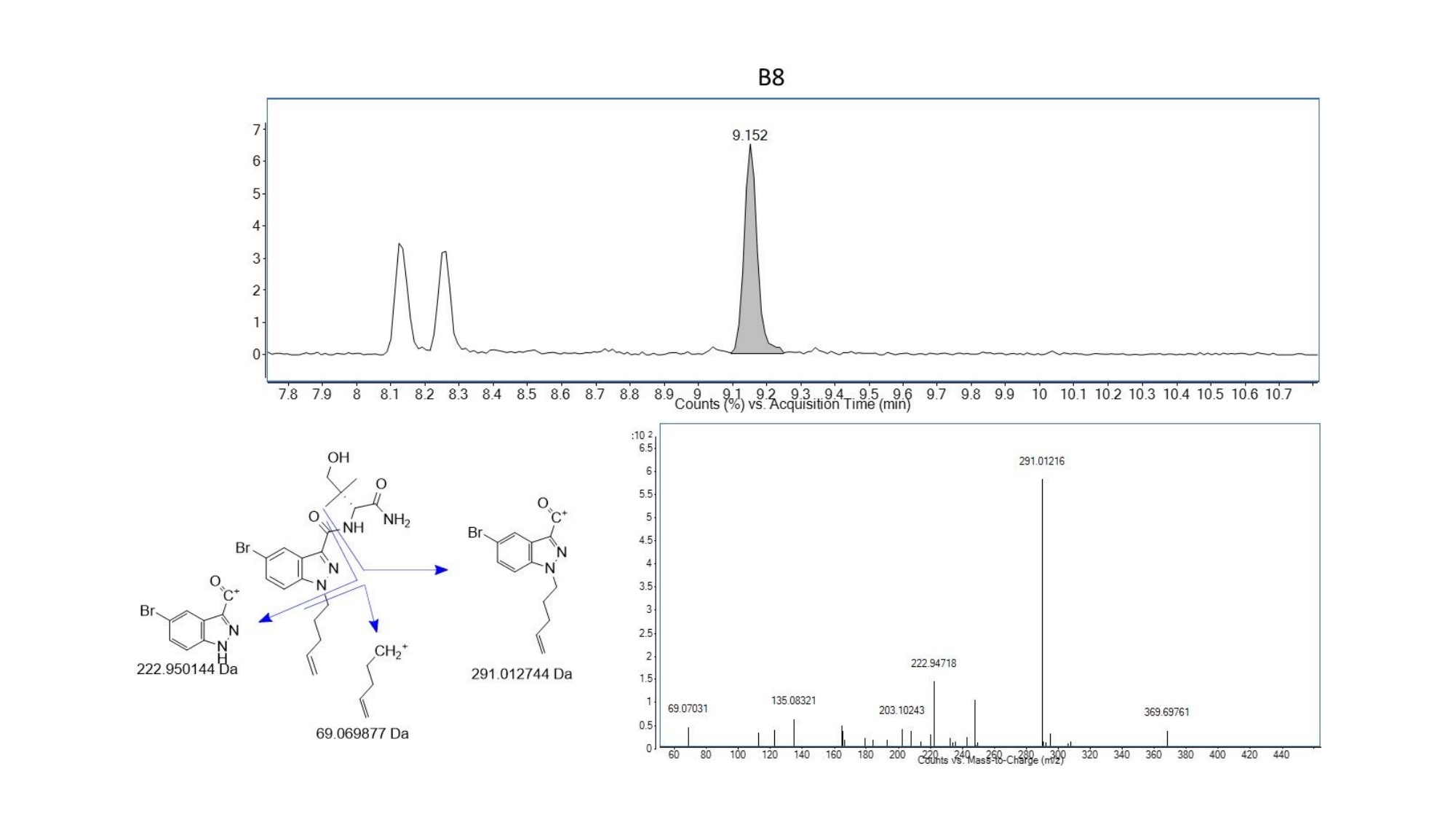

## Slide 23
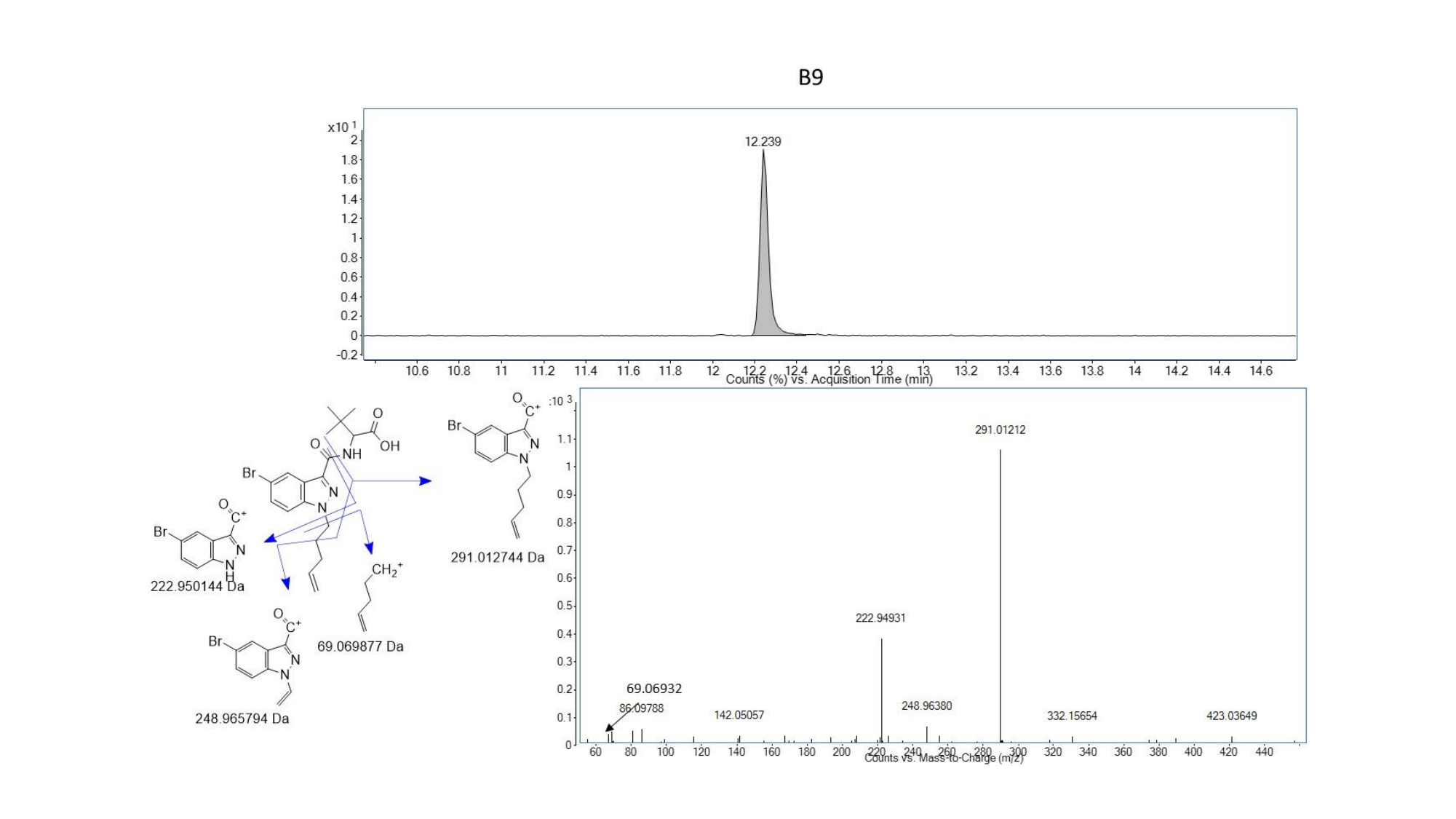

## Slide 24
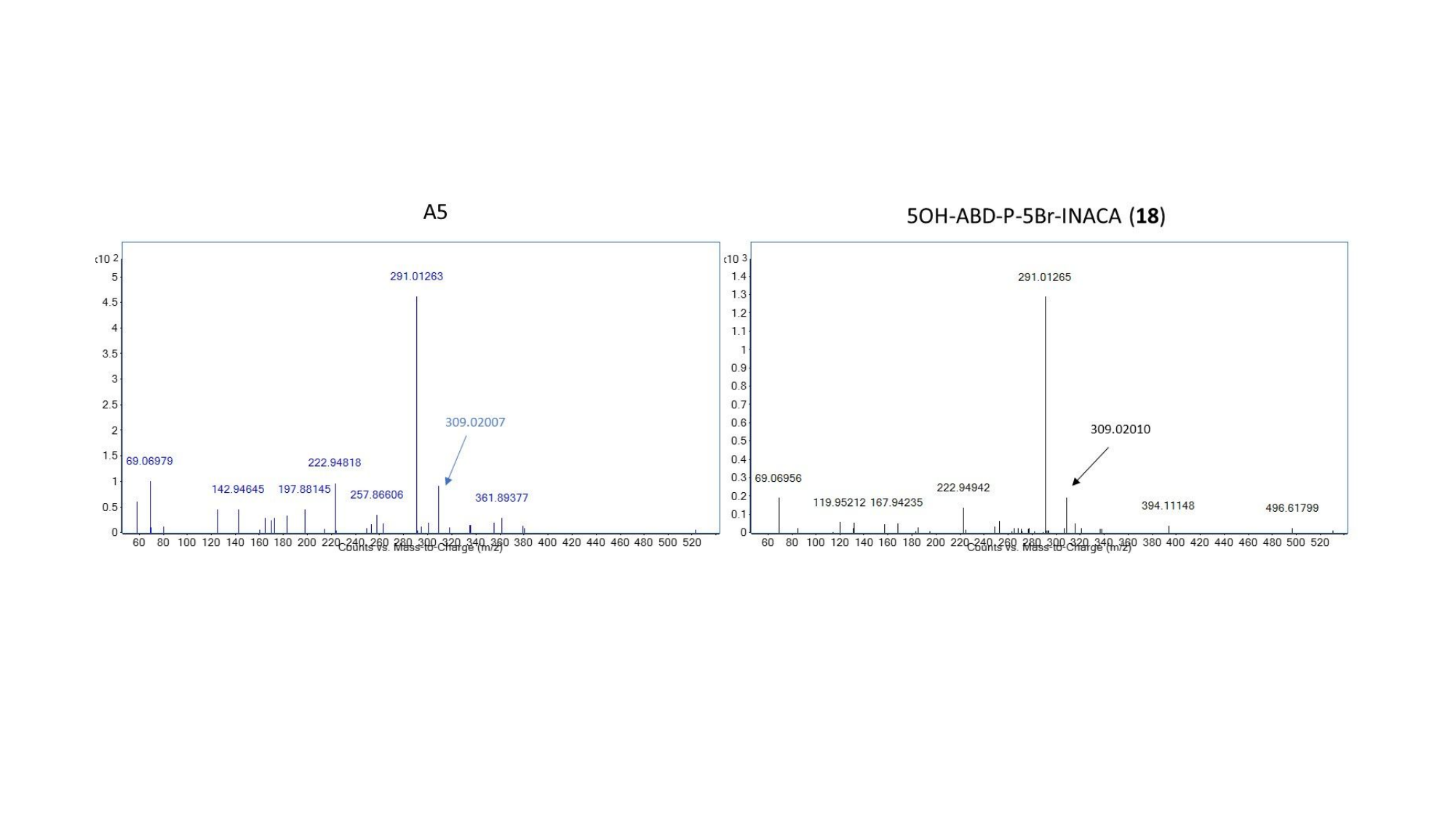

## Slide 25
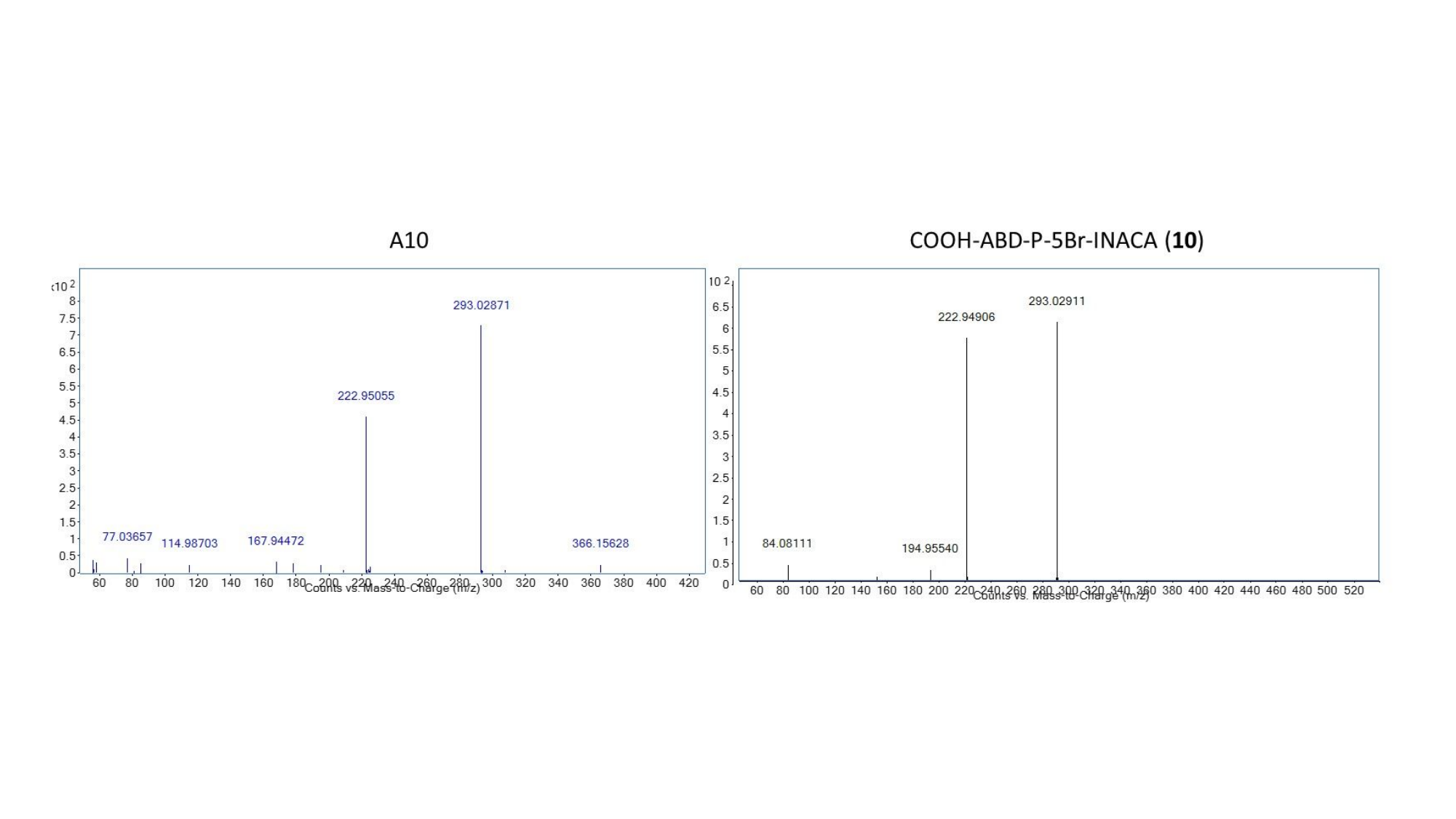

## Slide 26
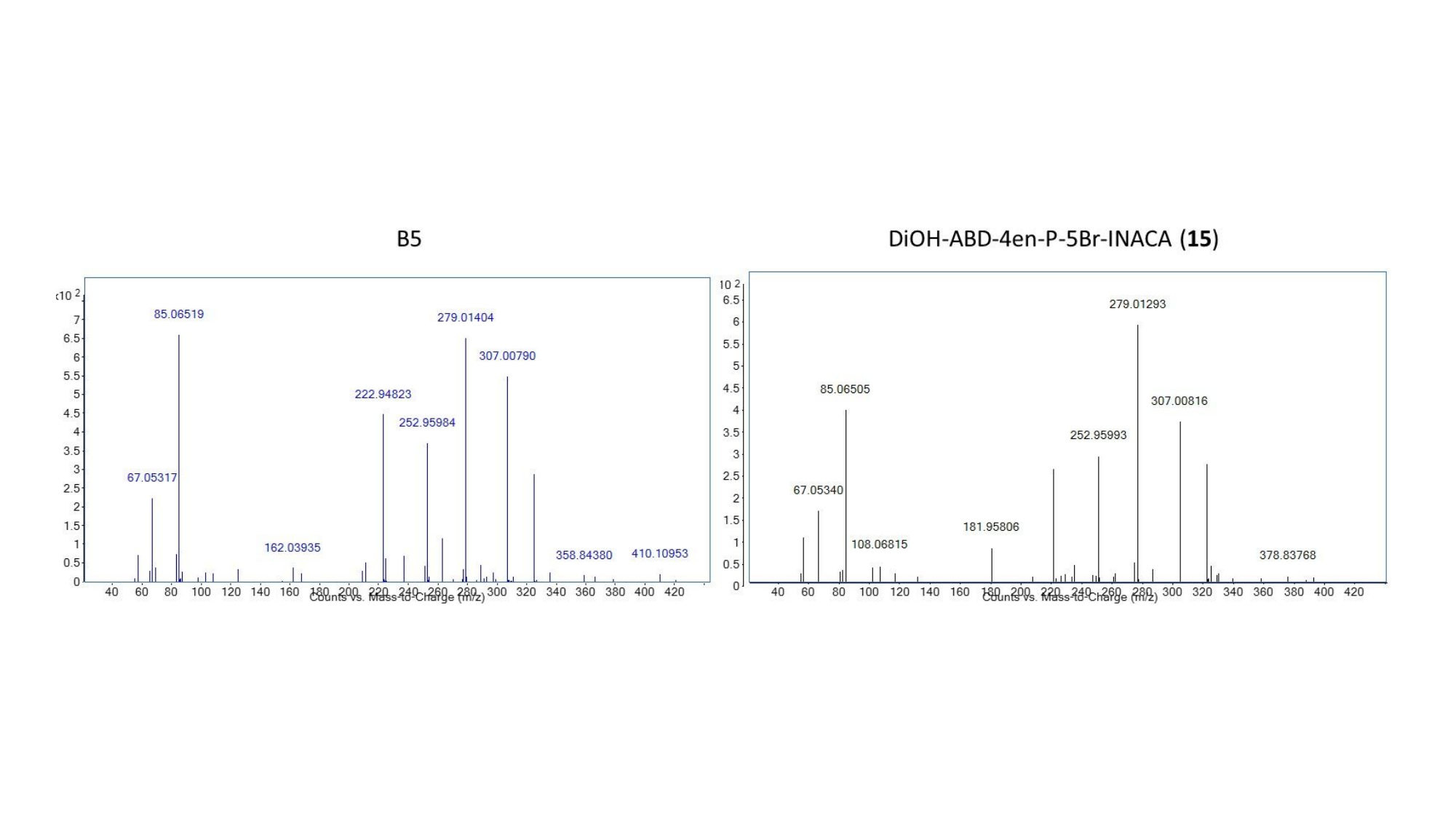

## Slide 27
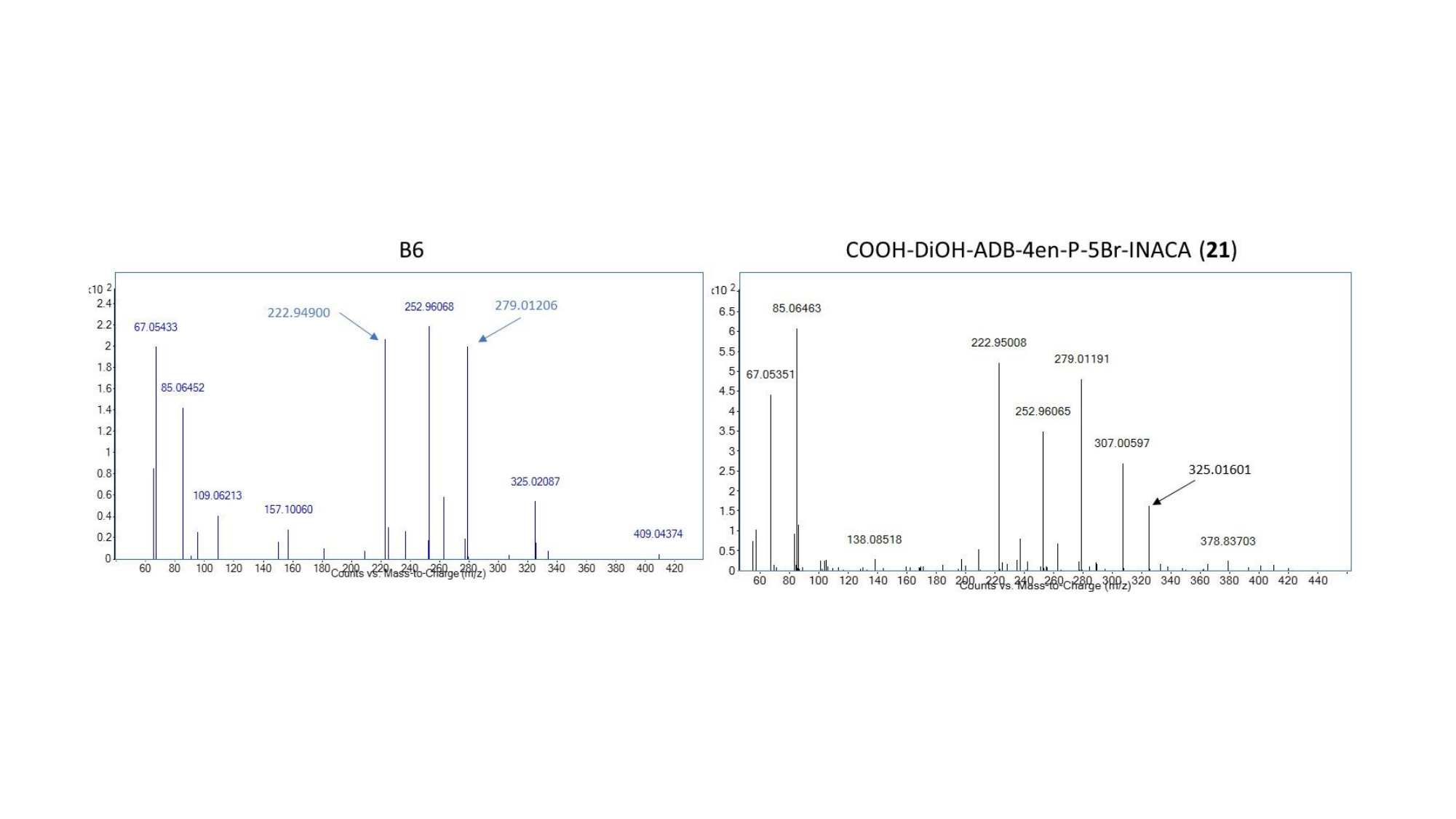

## Slide 28
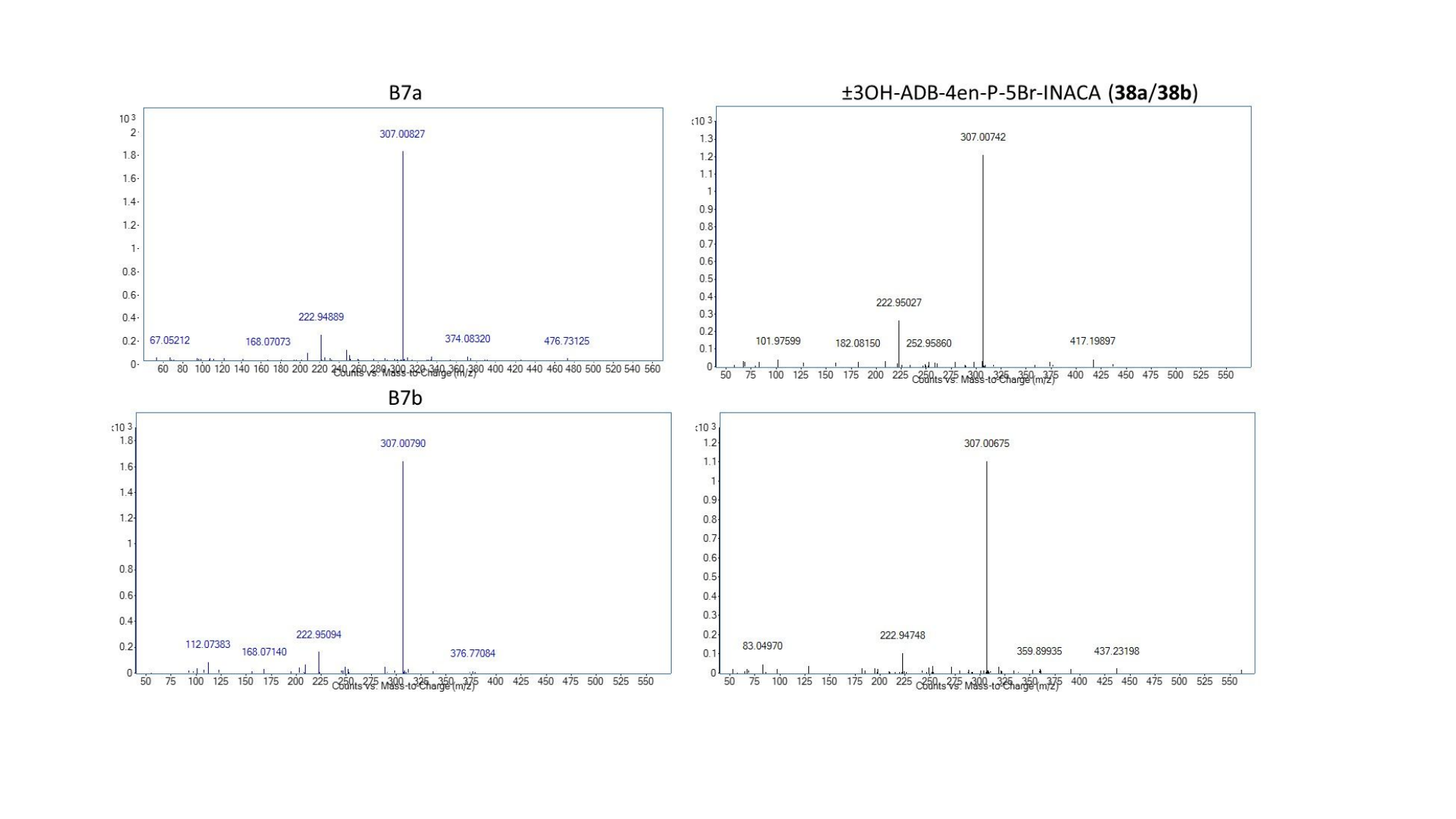

## Slide 29
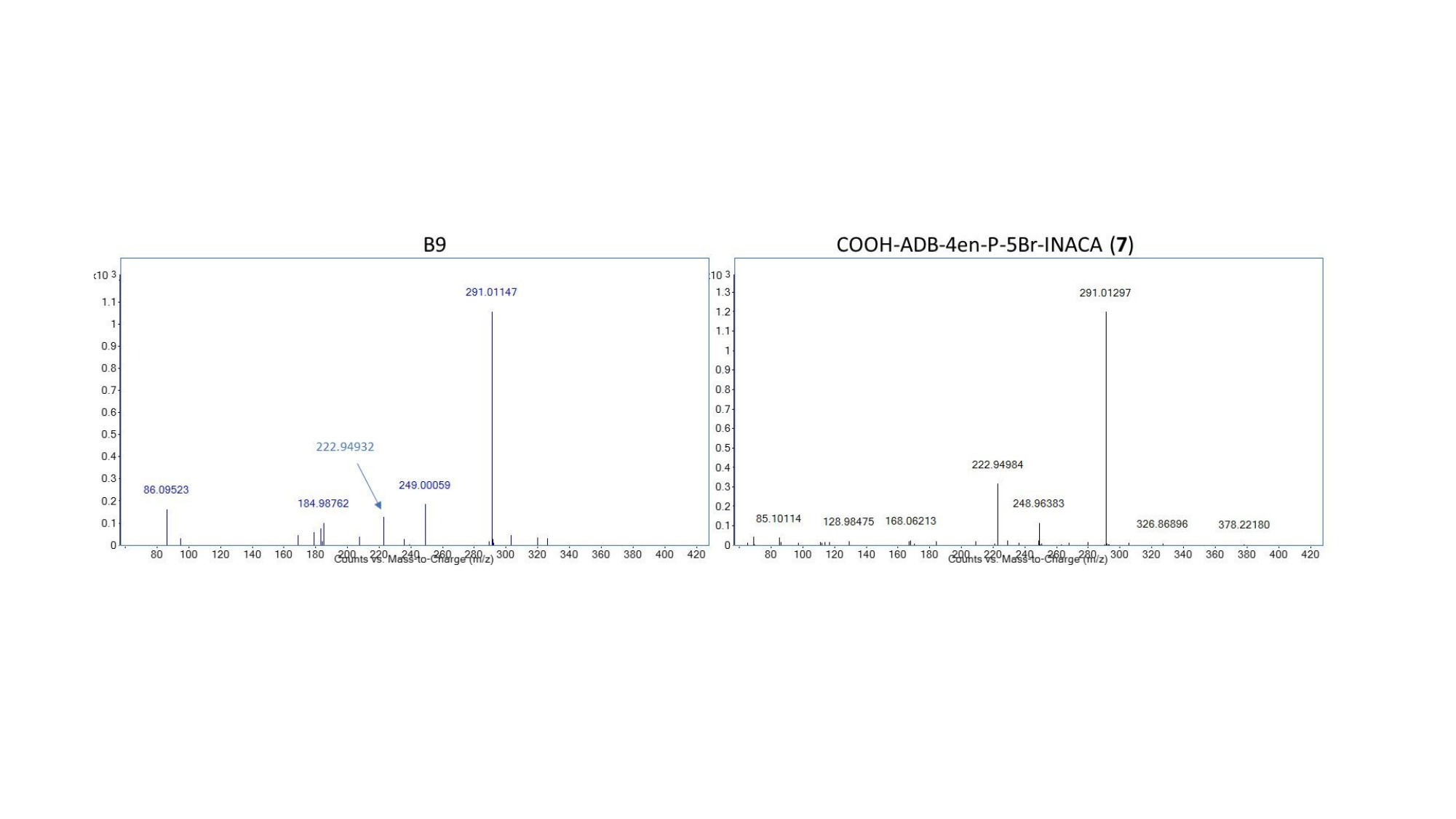

Supplement: Supplementary file 2 — Data S2. Supporting Information. [file DTA-17-701-s001.pptx]
